# Phylogenomic Analyses and Comparative Studies on Genomes of the *Bifidobacteriales*: Identification of Molecular Signatures Specific for the Order *Bifidobacteriales* and Its Different Subclades

**DOI:** 10.3389/fmicb.2016.00978

**Published:** 2016-06-27

**Authors:** Grace Zhang, Beile Gao, Mobolaji Adeolu, Bijendra Khadka, Radhey S. Gupta

**Affiliations:** ^1^Department of Biochemistry and Biomedical Sciences, McMaster UniversityHamilton, ON, Canada; ^2^CAS Key Laboratory of Tropical Marine Bio-resources and Ecology, Guangdong Key Laboratory of Marine Materia Medica, South China Sea Institute of Oceanology, Chinese Academy of SciencesGuangzhou, China

**Keywords:** molecular signatures for bifidobacteria, phylogeny, taxonomy, conserved signature indels, conserved signature proteins, *Bifidobacterium asteroides*-clade, *Scardovia*-clade

## Abstract

The order *Bifidobacteriales* comprises a diverse variety of species found in the gastrointestinal tract of humans and other animals, some of which are opportunistic pathogens, whereas a number of others exhibit health-promoting effects. However, currently very few biochemical or molecular characteristics are known which are specific for the order *Bifidobacteriales*, or specific clades within this order, which distinguish them from other bacteria. This study reports the results of detailed comparative genomic and phylogenetic studies on 62 genome-sequenced species/strains from the order *Bifidobacteriales*. In a robust phylogenetic tree for the *Bifidobacteriales* constructed based on 614 core proteins, a number of well-resolved clades were observed including a clade separating the *Scarodvia*-related genera (*Scardovia* clade) from the genera *Bifidobacterium* and *Gardnerella*, as well as a number of previously reported clusters of *Bifidobacterium* spp. In parallel, our comparative analyses of protein sequences from the *Bifidobacteriales* genomes have identified numerous molecular markers that are specific for this group of bacteria. Of these markers, 32 conserved signature indels (CSIs) in widely distributed proteins and 10 signature proteins are distinctive characteristics of all sequenced *Bifidobacteriales* species and provide novel and highly specific means for distinguishing these bacteria. In addition, multiple other molecular signatures are specific for the following clades of *Bifidobacteriales*: (i) 5 CSIs specific for a clade comprising of the *Scardovia*-related genera; (ii) 3 CSIs and 2 CSPs specific for a clade consisting of the *Bifidobacterium* and *Gardnerella* spp.; (iii) multiple other signatures demarcating a number of clusters of the *B. asteroides*-and *B. longum*- related species. The described molecular markers provide novel and reliable means for distinguishing the *Bifidobacteriales* and a number of their clades in molecular terms and for the classification of these bacteria. The *Bifidobacteriales*-specific CSIs, found in important proteins, are predicted to play important roles in modifying the cellular functions of the affected proteins. Hence, biochemical studies on the cellular functions of these CSIs could lead to discovery of novel characteristics of either all *Bifidobacteriales*, or specific groups of bacteria within this order. Some of the functions affected/modified by these genetic changes could also be important for the probiotic/pathogenic activities of the bifidobacteria.

## Introduction

The order *Bifidobacteriales* contains a large collection of bacterial species, many of which are significant constituents of the gastrointestinal tract of humans, other mammals, birds and honey bees (Biavati et al., 2000; Biavati and Mattarelli, 2006; Turroni et al., 2009, 2011; Biavati, 2012; Milani et al., 2014). In addition to widely recognized health-promoting effects of bifidobacterial species (Leahy et al., 2005; Ventura et al., 2009a; Cronin et al., 2011), some members of the group found in human and animal oral cavities are implicated in the development of dental caries (Huys et al., 2007; Mantzourani et al., 2009; Ventura et al., 2009b). Additionally, *Gardnerella vaginalis* is indicated to play an important role in the pathogenesis of bacterial vaginosis and urinary tract infections (Smith et al., 1992; Bradshaw et al., 2006; Alves et al., 2014; Kenyon and Osbak, 2014). The order *Bifidobacteriales* is part of the phylum Actinobacteria (Ventura et al., 2007b; Zhi et al., 2009; Gao and Gupta, 2012) and it harbors a single family, *Bifidobacteriaceae*, containing >50 recognized species (Biavati, 2012; Lugli et al., 2014; Milani et al., 2014; Parte, 2014) that are grouped into eight genera: *Aeriscardovia, Alloscardovia, Bifidobacterium, Gardnerella, Pseudoscardovia, Neoscardovia, Parascardovia*, and *Scardovia* (Jian and Dong, 2002; Simpson et al., 2004; Huys et al., 2007; Biavati and Mattarelli, 2012; García-Aljaro et al., 2012; Storms and Vandamme, 2012; Killer et al., 2013). Of these, the genus *Bifidobacterium*, encompassing 39 species and 9 subspecies, forms the largest group and accounts for more than 75% of the described taxa within the order *Bifidobacteriales* (Biavati and Mattarelli, 2012; Milani et al., 2014).

Phylogenetic analyses based on 16S rRNA, as well as sequences for a number of housekeeping genes/proteins, are the main approaches used in the past to distinguish among the *Bifidobacteriales* species and genera (Miyake et al., 1998; Ventura and Zink, 2003; Ventura et al., 2004, 2006, 2007a; Biavati and Mattarelli, 2006; Yarza et al., 2008; Bottacini et al., 2010; Turroni et al., 2011; Mattarelli et al., 2014). In recent years, complete or draft genome sequences have become available for all currently recognized *Bifidobacterium* species and subspecies (Ventura et al., 2009b; Milani et al., 2014). Based on these sequences, a panel of multiplex PCR primers has been developed enabling rapid and specific identification of different *Bifidobacterium* species and subspecies (Ferrario et al., 2015). Based on genome sequences, two recent studies have also examined the evolutionary relationships among *Bifidobacterium* species employing large datasets of sequences comprising the core proteins of this genus (Lugli et al., 2014; Sun et al., 2015). The robust phylogenetic trees obtained in these studies provide important insights concerning the evolutionary relationships among the *Bifidobacterium* species and they strongly support the existence of 6-7 distinct clusters within this genus. These clusters are referred to as the *B. asteroides, B. pseudolongum, B. longum, B. bifidum, B. adolescentis, B. pullorum*, and *B. boum* groups (Lugli et al., 2014; Sun et al., 2015). Similar clusters are also observed in phylogenetic trees based on the 16S and 23S rRNA genes as well trees based on other gene/protein sequences. Comparative analyses of the *Bifidobacterales* genomes are also providing useful insights concerning species-specific characteristics that are suggested to play important roles in the adaptation of particular species to either human or insect gut environment (Ventura et al., 2009b; Bottacini et al., 2010, 2012; Turroni et al., 2010).

Due to the health-promoting effects of bifidobacteria, it is of much interest to identify genetic and biochemical characteristics that are specific for the *Bifidobacteriales* or particular groups/clusters within this order of bacteria. Currently, very few such characteristics are known. One important class of genome sequence-based molecular markers, which have proven very useful for evolutionary, taxonomic and functional studies are conserved signature insertions or deletions (CSIs) that are uniquely present in the genes/proteins homologs from a defined group of organisms (Gao and Gupta, 2005, 2012; Gupta, 2010, 2014). Conserved signature proteins (CSPs), which are genes/proteins that are uniquely found within a monophyletic group of organisms, provide another class of useful molecular makers for evolutionary and functional studies (Gao et al., 2006; Ventura et al., 2007a; Gao and Gupta, 2012; Gupta, 2016a,b). Both these types of markers constitute highly reliable characteristics of specific groups of organisms and they have been extensively utilized for the identification/demarcation of prokaryotic taxa of different ranks in molecular terms (Gao and Gupta, 2012; Gupta et al., 2013a,b, 2016).

In the present work, we report detailed phylogenetic and comparative analyses on protein sequences from the sequenced members of the order *Bifidobacteriales* in order to identify CSIs and CSPs that are specific for different groups within this order. These studies have led to identification of 32 CSIs in widely distributed proteins and 10 CSPs that are uniquely found in all or most of the genome sequenced *Bifidobacteriales* species providing novel molecular markers that distinguish this order from all other bacteria. In addition, our work has also identified multiple other CSIs and CSPs that distinguish a number of clades of *Bifidobacteriales*, including a clade consisting of the *Bifidobacterium* and *Gardnerella* species, another clade consisting of the *Scardovia*-related genera, and multiple signatures that are specific for different clusters of *B. asteroides* or *B. longum* related species. These signatures provide novel means for the identification and demarcation of the members of the described clades in molecular terms and for functional studies that could lead to discovery of novel biochemical and/or other novel properties of these bacteria.

## Methods

### Phylogenetic analysis

A phylogenetic tree for 62 genome-sequenced members from the order *Bifidobacteriales* was constructed based on concatenated sequences of 614 proteins. The protein families used in this phylogeny were identified using the UCLUST algorithm (Edgar, 2010) to identify proteins families present in at least 80% of the input genomes which shared at least 50% sequence identity and 50% sequence length. Each identified protein family was individually aligned using Clustal Omega (Sievers et al., 2011) and trimmed using Gblocks 0.91b (Castresana, 2000) with relaxed parameters (Talavera and Castresana, 2007). The concatenated dataset of the trimmed sequence alignments contained 197, 777 aligned amino acid residues. A maximum-likelihood tree based on this alignment was constructed using FastTree 2 (Price et al., 2010) employing the Whelan and Goldman model of protein sequence evolution (Whelan et al., 2001) and RAxML 8 (Stamatakis, 2014) using the Le and Gascuel model of protein sequence evolution (Le and Gascuel, 2008). SH-like statistical support values (Guindon et al., 2010) for each branch node in the final phylogenetic tree were calculated using RAxML 8 (Stamatakis, 2014). This process was completed using an internally developed software pipeline.

In parallel, a phylogenetic tree based on the 16S rRNA gene sequences of type strains covering all described species within the order *Bifidobacteriales* was also constructed. The 16S rRNA sequences were retrieved from Ribosomal Database Project (Cole et al., 2014) and aligned using the SINA aligner (Pruesse et al., 2012) to form a multiple sequence alignment that was 1604 aligned nucleotides long with common gaps removed. A maximum-likelihood phylogenetic tree based on this multiple sequence alignment was created using MEGA 6 employing the General Time-Reversible model of sequence evolution with branch support based on 1000 bootstrap replicates (Tamura et al., 2013).

### Identification of conserved signature indels

Conserved signature indels (CSIs) were identified by the procedures described in detail recently (Gupta, 2014). Briefly, BLASTp (Altschul et al., 1997) searches were performed on each protein in the genome of *Bifidobacterium adolescentis* ATCC 15703 (Accession number AP009256.1) against all available sequences in the GenBank non-redundant database. Multiple sequence alignments were then created using ClustalX (Jeanmougin et al., 1998) for proteins that returned high scoring matches from *Bifidobacteriales* and other prokaryotes. The alignments were then visually inspected for the presence of insertions or deletions that were flanked on both sides by at least 5-6 conserved amino acid residues in the neighboring 30–40 amino acids. Detailed BLASTp searches were then carried out on short sequence segments containing the indel and the flanking conserved regions (60-100 amino acids long) to determine the specificity of the indels. SIG_CREATE and SIG_STYLE (available on Gleans.net) were then used to create Signature files for CSIs that were specific for the *Bifidobacteriales* order or its subgroups as described in earlier work (Gupta et al., 2013a; Gupta, 2014). Due to space limitations, sequence information for all *Bifidobacterium* species, particularly for different subspecies of *B. longum, B. animalis, B. pseudolongum*, and *B. thermacidophilum*, is not shown in the presented alignment files. However, unless otherwise noted, all of the described CSIs are specific for the indicated groups (i.e., similar CSIs were not present in the protein homologs from other bacteria in the top 500 Blast hits). It should be noted that significant blast hits for a number of CSIs and CSPs described here are also observed for one of the following three *Chlamydia trachomatis* strains (SwabB1, H1 IMS, and H17 IMS) deposited by the Sanger Institute. We suspect that these anomalous results are due to cross contamination of the sequenced cultures from the above *Chlamydia trachomatis* strains by a *Gardnerella* vaginalis strain. We have communicated our concern with the supporting evidence to the Sanger Institute.

### Identification of conserved signature proteins

BLASTp searches were carried out to examine the specificity of some previously described conserved signature proteins (CSPs), which were indicated to be specific for the order *Bifidobacteriales* (Gao and Gupta, 2012). Additionally, limited work to identify CSPs for the *B. asteroides* group of species was carried out by conducting BLASTp searches on all proteins from the genomes of *Bifidobacterium asteroides* (Bottacini et al., 2012) as query sequences. BLASTp searches were performed against all available sequences in the GenBank non-redundant sequence database and the results of these searches were then manually inspected, as described in earlier work (Gao et al., 2006; Gao and Gupta, 2012), for proteins for which all significant hits were from the *B. asteroides* group of species.

### Homology modeling of elongation factor Tu from *Bifidobacterium longum*

Homology models of EF-Tu homolog from *Bifidobacterium longum* were built using the solved crystallographic structure of EF-Tu from *Escherichia coli* (PDB ID: 3U6K) as the template. Initially, 200 models were generated using MODELER v9.14 (Sali and Blundell, 1993) and ranked/selected using assigned discrete optimized potential (DOPE) scores (Shen and Sali, 2006). The model with the highest DOPE score was then submitted to the ModRefiner program to obtain atomic-level energy minimization and to obtain a model with reliable stereochemistry quality (Xu and Zhang, 2011).

## Results

### Phylogenetic analysis of the species from the order *Bifidobacteriales*

**Figure 1:**
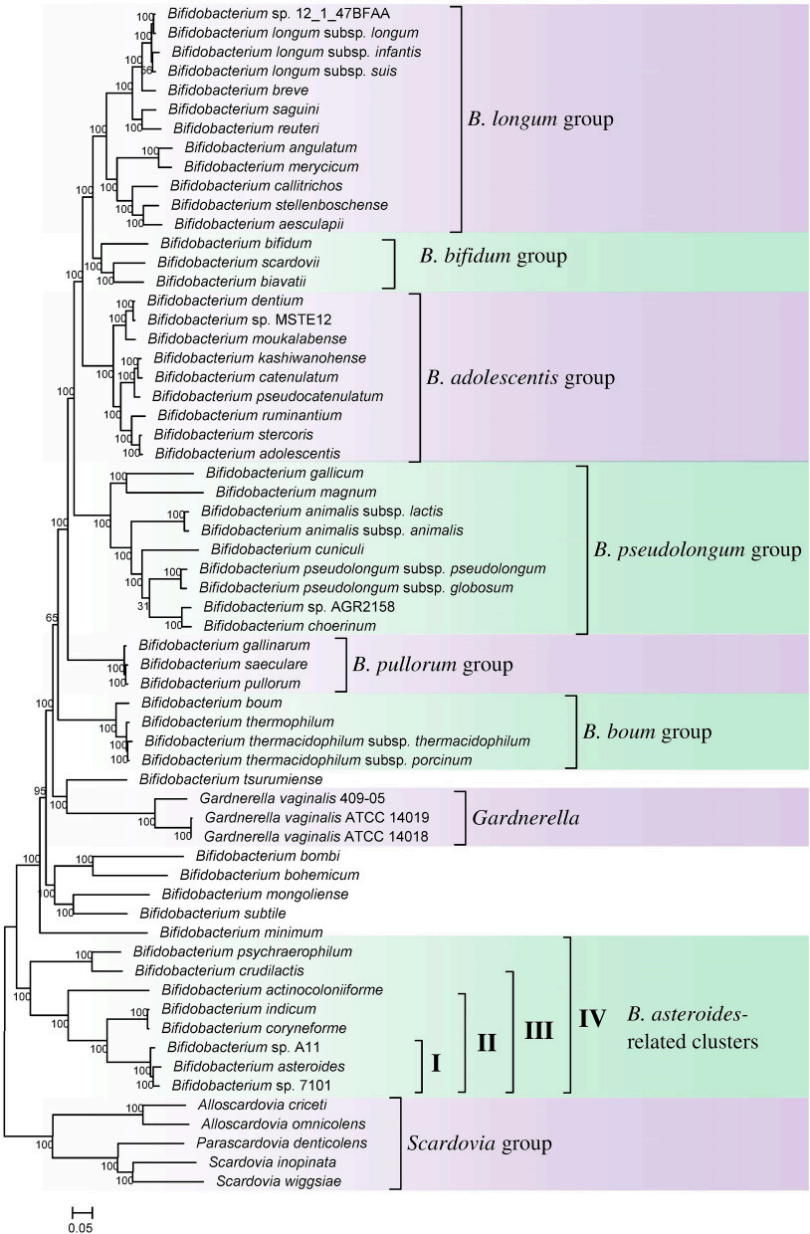
**A maximum-likelihood tree based on concatenated sequences of 614 core proteins from 62 sequenced genome-sequenced members of the order *Bifidobacteriales***. The tree was rooted at the midpoint and SH-like support values are indicated at nodes. A number of different clades/clusters that are consistently observed in phylogenetic trees are marked.

Phylogenomic analyses of members of the genus *Bifidobacterium* have been previously reported based on core protein sequences from 45 and 48 described species from this genus (Lugli et al., 2014; Sun et al., 2015). However, these studies did not include the other members of the order *Bifidobacteriales* such as *Gardnerella, Scardavia, Alloscardovia*, and *Parascardovia*, as well as several unnamed *Bifidobacterium* spp. (viz. strains A11, 7101, AGR2158, MSTE12, 12.1.47BFAA) whose genomes have been sequenced. Additionally, the genome sequence of a recently described species *B. aesculapii* is also now available (Toh et al., 2015). To comprehensively examine the evolutionary relationships among different members of the order *Bifidobacteriales*, a phylogenetic tree was constructed for all 62 genome sequenced members of the family which included 54 *Bifidobacterium* species/strains, 5 species from *Scardovia* and related genera (viz. *Alloscardovia* and *Parascardovia*) and three strains of *Gardnerella vaginalis*. The tree was constructed based on the concatenated sequences of 614 universally or nearly universally present core proteins for which sequence information could be obtained from the 62 sequenced genomes. A maximum-likelihood tree based on these sequences, which represents the most comprehensive phylogenetic analysis of the order *Bifidobacteriales* to date, is presented in Figure 1.

In the tree shown, members of the order *Bifidobacteriales*, at the highest level, form two main clusters. One of these clusters referred to as the *Scardovia* cluster groups together the genera *Scardovia, Parascardovia*, and *Alloscardovia*, whereas the second cluster is comprised of members of the genus *Bifidobacterium* and *Gardnerella*. Importantly in this tree, as well as in an earlier study in a phylogenetic tree based on concatenated sequences for RpoB, RpoC, and GyrB proteins, different strains of *Gardnerella vaginalis* were found to branch in between the *Bifidobacterium* species (Gao and Gupta, 2012), making the genus *Bifidobacterium* polyphyletic. Earlier phylogenetic studies on members of the genus *Bifidobacterium* have identified a number of different clusters, which are referred to as the *B. asteroides, B. pseudolongum, B. longum, B. bifidum, B. adolescentis, B. pullorum*, and *B. boum* groups (Ventura et al., 2006; Turroni et al., 2011; Lugli et al., 2014; Sun et al., 2015). The existence of these groups/clusters is also confirmed and supported by the tree shown in Figure 1. Of these clusters, the species-related to *B. asteroides* cluster exhibited the deepest branching within the genus *Bifidobacterium*, as also observed in earlier work (Lugli et al., 2014; Sun et al., 2015). The *B. asteroides* clade is generally demarcated as being comprised of the *B. asteroides, B. indicum, B. coryneforme*, and *B. actinocoloniiforme* species (marked as cluster III in Figure 1). However, as discussed later, a number of clusters, marked I, II, and IV, which are either part of the *B. asteroides* clade or are related to this clade are also distinguished in phylogenetic trees and by the CSIs identified in this work.

We have also created a phylogenetic tree based on 16S rRNA gene sequences of all named *Bifidobacteriales* species (Supplementary Figure S1). The overall branching pattern in the 16S rRNA tree is similar to that observed in the concatenated protein tree with *Scardovia* and related genera forming the deepest branches in the tree and the genera *Scardovia, Alloscardovia*, and *Parascardovia* were part of one of the deepest branching clusters. The different clusters of the *Bifidobacterium* spp. that are observed in the concatenated protein tree were also supported by the 16S rRNA tree and *G. vaginalis* was found to branch in between these clusters. The polyphyletic nature of the genus *Bifidobacterium* in 16S rRNA gene based phylogenies is also observed in earlier work (Yilmaz et al., 2014).

### Identification of molecular markers that are specific for the order *Bifidobacteriales*

The main focus of this work is the identification of molecular characteristics that are specific for the *Bifidobacteriales* species and could be used for their identification as well as functional studies. As noted earlier, conserved inserts and deletions (i.e., indels or CSIs) in genes/proteins and conserved signature proteins that are uniquely found in a phylogenetically coherent group of organisms provide very useful molecular markers for such purposes. The indels that provide useful molecular markers are of defined size and they are flanked on both sides by conserved regions to ensure that they are reliable characteristics (Gupta, 1998; Gupta and Griffiths, 2002; Ajawatanawong and Baldauf, 2013). These conserved indels in gene/protein sequences result from highly specific and rare genetic changes, hence when such an indel is uniquely found in a phylogenetically coherent group of species, its simplest explanation is that the genetic change responsible for it occurred once in a common ancestor of the indicated group and then the change was passed on to various descendants (Gupta, 1998, 2014; Rokas and Holland, 2000; Gao and Gupta, 2005). Based upon the presence or absence of a conserved indel in outgroup species, it is also possible to determine whether a given indel represents an insert or a deletion (Gupta, 1998; Gao and Gupta, 2012).

**Figure 2:**
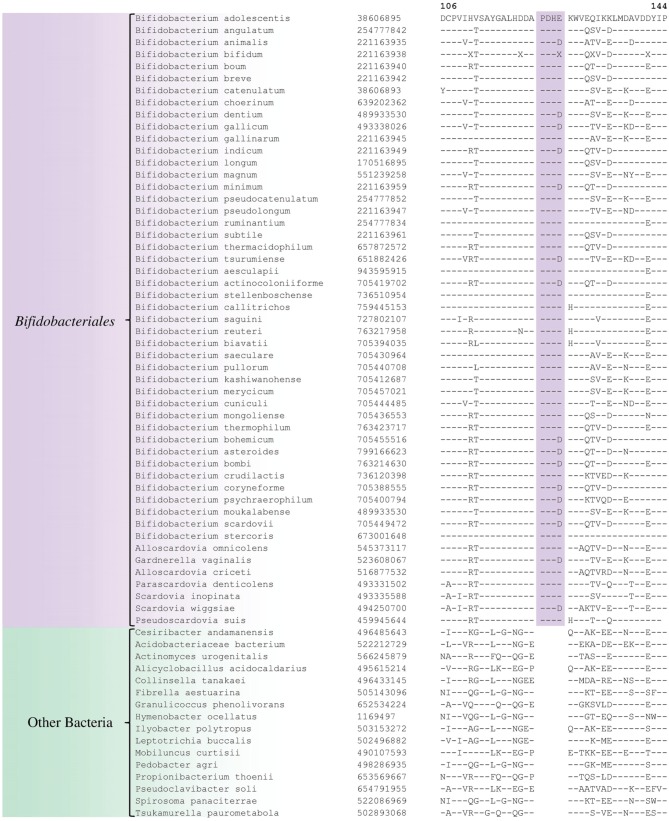
**Partial sequence alignment of the protein synthesis elongation factor-Tu showing a 4 aa insertion in a conserved region that is specific for members of the order *Bifidobacteriales***. The dashes in this alignment as well as all other alignments show identity with the amino acid on the top line. The Genebank Identification numbers of the protein sequences are shown, and the topmost numbers indicate the position of this sequence in the species shown on the top line. Due to space constraints, sequence information for different subspecies is not shown. However, unless otherwise indicated, these CSIs are present in the sequenced subspecies of *B. longum, B. animalis, B. pseudolongum*, and *B. thermacidophilum*. Information for large numbers of other CSIs, which are also specific for the order *Bifidobacteriales* is presented in Table 1 and Supplementary Figures S2–S32.

Comparative analyses of protein sequence alignments from bifidobacteia species carried out in this work have led to the identification of 32 CSIs in a broad range of highly conserved proteins, which are specifically found in different *Bifidobacteriales* taxa (see Table 1). One example of a CSI that is specific for all members of the order *Bifidobacteriales* is shown in Figure 2. In this case, a 4 amino acid (aa) insertion is present in a highly conserved region of the protein synthesis elongation factor EF-Tu, which is commonly shared by all sequenced bifidobacteria species, but it is not found in any other bacteria in the top 500 BLAST hits. The protein EF-Tu is a highly conserved protein, which is universally present in all organisms (Harris et al., 2003) and the 4 aa CSI in this protein is a distinctive characteristic of homologs from all sequenced *Bifidobacteriales* species. Sequence information for 31 other CSIs, which are also specifically shared by members of the order *Bifidobacteriales*, and which are present in proteins involved in different other functions, is provided in Supplementary Figures S2–S32 and some of their characteristics are summarized in Table 1. Barring an isolated exception, all of the CSIs listed in Table 1 are specifically found in different members of the order *Bifidobacteriales* and are not present in the protein homologs from other bacteria. Due to their specific presence in bifidobacteria species, the described CSIs provide novel molecular markers for distinguishing and demarcating members of the order *Bifidobacteriales* from all other bacteria. We have previously described 14 CSPs, whose homologs were specifically found in the 13 different sequenced bifidobacteria species that were available at the time (Gao and Gupta, 2012). Updated BLASTp searches on the sequences of these CSPs confirm that 10 of these CSPs, information for whom is provided in Table 2, are still distinctive characteristics of members of the order *Bifidobacteriales* and they provide additional molecular markers for identification and functional studies on bifidobacteria.

**Table 1:** **Characteristics of conserved signature indels that are Specific for the order *Bifidobacteriales***.

**Protein name**	**GI number**	**Figure no**.	**Indel size**	**Indel region^a^**
Elongation factor Tu	38606895	Figure 2	4 aa ins	106–144
DNA topoisomerase I	489904111	Supplementary Figure S2	1 aa del	31–80
DNA polymerase sliding clamp subunit	408500301	Supplementary Figure S3	1 aa ins	79–118
Beta-galactosidase	504834401	Supplementary Figure S4	1–2 aa ins	371–423
Ketol-acid reductoisomerase	651881972	Supplementary Figure S5	2 aa del	242–284
Serine-pyruvate aminotransferase	489903803	Supplementary Figure S6	2 aa ins	74–119
50S ribosomal protein L21	489922190	Supplementary Figure S7	1 aa ins	42–82
Methionine aminopeptidase	547078960	Supplementary Figure S8	1 aa ins	34–70
Bifunctional acetaldehyde-CoA/alcohol dehydrogenase	500062906	Supplementary Figure S9	1 aa ins	534–574
Bifunctional acetaldehyde-CoA/alcohol dehydrogenase	500062906	Supplementary Figure S10	1 aa ins	809–845
Formate acetyltransferase	500063439	Supplementary Figure S11	2 aa ins	367–416
ATP synthase F0 subunit A	547078870	Supplementary Figure S12	1 aa ins	131–163
Peptide chain release factor 1	489924412	Supplementary Figure S13	2 aa ins	197–237
Arginine ABC transporter ATP-binding protein	489905014	Supplementary Figure S14	1 aa del	224–280
Transketolase	489905793	Supplementary Figure S15	4 aa ins	338–388
Histidine kinase	547084095	Supplementary Figure S16	1 aa ins	362–405
DNA repair ATPase	489905284	Supplementary Figure S17	3 aa ins	353–394
n-acetyl-gamma-glutamyl-phosphate reductase	547072106	Supplementary Figure S18	1 aa ins	10–60
Arginine biosynthesis bifunctional protein ArgJ	547072098	Supplementary Figure S19	1 aa ins	1–42
Excinuclease ABC subunit C	494111998	Supplementary Figure S20	1 aa ins	103–150
Cysteine desulfurase	500063210	Supplementary Figure S21	4 aa ins	54–105
2-C-methyl-D-erythritol 2,4-cyclodiphosphate synthase	489906135	Supplementary Figure S22	1 aa ins	58–81
Argininosuccinate lyase	547072080	Supplementary Figure S23	5 aa ins	405–454
CarD family transcriptional regulator	500063173	Supplementary Figure S24	1 aa ins	30–79
Acetyltransferase GNAT family	547074268	Supplementary Figure S25	1 aa ins	112–152
Acetyltransferase GNAT family	547074268	Supplementary Figure S25	2 aa ins	112–152
Signal recognition particle protein	489904236	Supplementary Figure S26	1 aa ins	70–110
50S ribosomal protein L13	489923970	Supplementary Figure S27	1 aa del	51–90
DNA gyrase B subunit protein	547082727	Supplementary Figure S28	2 aa del	637–686
Hemolysin III	489923478	Supplementary Figure S29	1 aa del	171–216
Pseudouridine synthase	547071034	Supplementary Figure S30	1 aa ins	56–95
Guanylate kinase	500063064	Supplementary Figure S31	4 aa ins	85–124
D-alanine–D-alanine ligase	493336643	Supplementary Figure S32	2–7 aa ins	202–244

a *The indel region indicates the region of the protein where the described CSI is present*.

**Table 2:** **Conserved signature proteins that are uniquely found in the *Bifidobacteriales***.

**Accession no**.	**Length**	**Function**	**Species specificity**
ZP_02917512	73	Unknown, hypothetical	*Bifidobacteriales*
ZP_02917322	275	Unknown, hypothetical	*Bifidobacteriales*
ZP_02917261	336	Unknown, hypothetical	*Bifidobacteriales*
ZP_02917147	228	Unknown, hypothetical	*Bifidobacteriales*
ZP_02917106	399	Unknown, hypothetical	*Bifidobacteriales*
ZP_02919152	201	Unknown, hypothetical	*Bifidobacteriales*
ZP_02918813	121	Unknown, hypothetical	*Bifidobacteriales*
ZP_02916931	84	Unknown, hypothetical	*Bifidobacteriales*
ZP_02917770	76	Unknown, hypothetical	*Bifidobacteriales*
ZP_02918933	321	Unknown, hypothetical	*Bifidobacteriales*
ZP_02917048	222	Unknown, hypothetical	*Bifidobacterium* and *Gardnerella*
ZP_02919141	299	Unknown, hypothetical	*Bifidobacterium* and *Gardnerella*
ZP_02919088	260	Unknown, hypothetical	*Bifidobacterium*
ZP_02918031	283	Unknown, hypothetical	*Bifidobacterium*
ZP_02919040	189	Unknown, hypothetical	*Bifidobacterium*
WP_015021123.1	152	Unknown, hypothetical	*B. asteroides* cluster I
WP_033511744.1	116	Unknown, hypothetical	*B. asteroides* cluster II
WP_015021403.1	283	Unknown, hypothetical	*B. asteroides* cluster III
WP_015022574.1	190	Unknown, hypothetical	*B. asteroides* cluster III
WP_015022150.1	300	Unknown, hypothetical	*B. asteroides* cluster III

*The species that are part of the B. asteroides clusters I, II, and III are indicated in Figure 1*.

### Molecular signatures for some of the subclades of *Bifidobacteriales*

**Figure 3:**
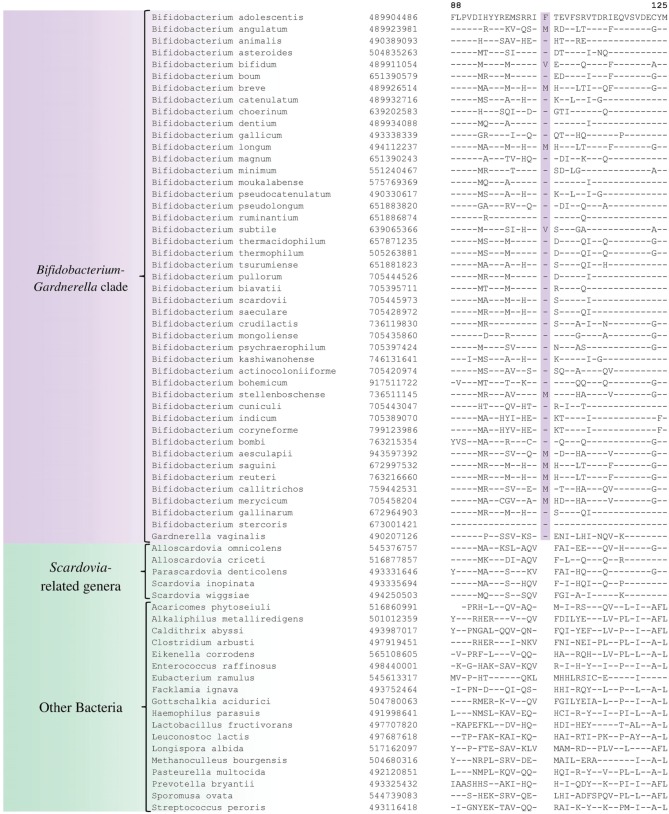
**Partial sequence alignment of DNA polymerase IV showing a 1 aa insertion that is specific for the *Bifidobacterium* and *Gardnerella* species, but not found in any other *Bifidobacteriales***. Information for other CSIs specific for this clade is presented in Table 3 and Supplementary Figures S33–S35.

In the phylogenetic tree based on concatenated protein sequences, *Bifidobacteriales* species form a number of different clusters. At the deepest level, of the two main clusters that are observed, one consists of the genus *Scardovia* and related genera, whereas the other is comprised of species from the genera *Bifidobacterium* and *Gardnerella*. In our analyses, we have also identified a number of CSIs and CSPs which distinguish these two clades of the *Bifidobacteriales*. Figure 3 shows one example of a CSI consisting of a 1 aa insertion in the DNA polymerase IV protein that is specifically found in different *Bifidobacterium* species and *Gardnerella*, but which is not found in any of the sequenced *Scardovia*-related genera of the *Bifidobacteriales*. Two other CSIs in the ribosomal RNA small subunit methyltransferase E protein and GTP-binding protein YchF are also specifically shared by members of the genera *Bifidobacterium* and *Gardnerella*. Sequence information for these CSIs is presented in Supplementary Figures S33, S34 and some of their characteristics are summarized in Table 3. Additionally, we have also confirmed that the homologs of 5 of the 6 previously described CSPs (Gao and Gupta, 2012), information for which is summarized in Table 2, are also present in only members of these two genera.

**Table 3:** **Characteristics of Conserved Signature Indels Distinguishing a number of subgroups within the order *Bifidobacteriales***.

**Protein name**	**GI number**	**Figure number**	**Indel size**	**Indel position**	**Specificity**
DNA polymerase IV	489904486	Figure 3	1 aa ins	88–125	*Bifidobacterium-Gardnerella*
Ribosomal RNA small subunit methyltransferase E	547081721	Supplementary Figure S33	3 aa del	118–160	*Bifidobacterium-Gardnerella*
GTP-binding protein YchF	547055080	Supplementary Figure S34	1 aa ins	309–354	*Bifidobacterium-Gardnerella*
Cytochrome C	500062679	Supplementary Figure S35	3 aa del	730–765	*Bifidobacterium*
Triosephosphate isomerase	651360171	Figure 4	1 aa ins	251–286	Scardovia clade
FHA domain protein	493335662	Supplementary Figure S36	1 aa ins	37–67	Scardovia clade
Glycosyl transferase	648490110	Supplementary Figure S37	2 aa ins	23–67	Scardovia clade
PAC2 family protein	294458767	Supplementary Figure S38	2 aa ins	32–77	Scardovia clade
Phosphate ABC transporter substrate-binding protein	493336671	Supplementary Figure S39	2 aa ins	167–206	Scardovia clade
Phosphogluconate dehydrogenase	497766884	Figure 5	1 aa ins	360–401	*B. longum* cluster
PhoU family transcriptional regulator	489926631	Supplementary Figure S40	2 aa del	159–190	*B. longum* cluster
Cystathionine gamma-synthase	494112910	Supplementary Figure S41	2 aa ins	262–302	*B. longum* cluster
Transketolase	489905793	Supplementary Figure S42	1 aa ins	234–274	*B. longum, B. bifidum* and *B. adolescentis* clade
Purine biosynthesis protein purH	658453400	Figure 6A	1 aa ins	247–278	*B. asteroides* cluster II ^#^
Shikimate dehydrogenase	658453363	Supplementary Figure S43	1 aa ins	264–301	*B. asteroides* cluster II ^#^
5-methyltetrahydropteroyltriglutamate–homocysteine methyltransferase	504834759	Supplementary Figure S44	1 aa ins	336–369	*B. asteroides* cluster II ^#^
ABC transporter substrate-binding protein	504835116	Supplementary Figure S45	1 aa del	253–286	*B. asteroides* cluster II ^#^
5'-methylthioadenosine nucleosidase	504835309	Figure 6B	3 aa ins	1–33	*B. asteroides*-related cluster IV ^#^
Peptide ABC transporter ATP-binding protein	504834913	Supplementary Figure S46A	20 aa ins	76–127	*B. asteroides* cluster I^#^
N-acetyl-gamma-glutamyl-phosphate reductase	504834965	Supplementary Figure S46B	1 aa ins	34–74	*B. asteroides* cluster I^#^

# *The B. asteroides-related cluster I, II, and IV are demarcated in Figure 1*.

**Figure 4:**
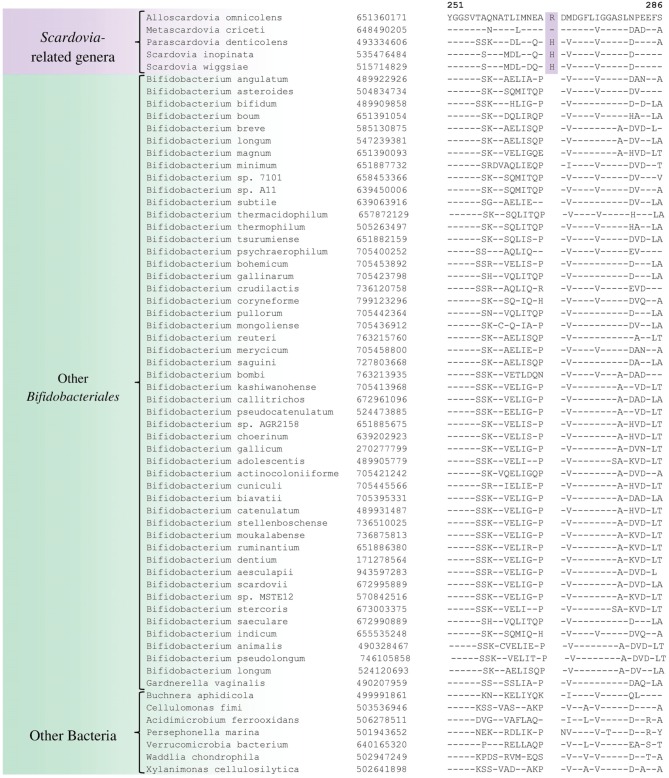
**Example of 1 aa conserved signature indel in the protein triosephosphate isomerase that is specific for the *Scardovia* clade comprising of the genera *Scardovia, Parascardovia, Metascardovia*, and *Alloscardovia***. Information for other CSIs specific for this clade is presented in Table 3 and Supplementary Figures S36–S39.

We have also identified a number of CSIs that are commonly and specifically shared by members of the genus *Scardovia* and related genera for which sequence information is available. One example of a CSI which is specifically found in members of the genera *Scardovia, Parascardovia* and *Alloscardovia*, consisting of 1 aa insertion in the triosephosphate isomerase protein, is presented in Figure 4. Four other CSIs in four different proteins (viz. FHA domain protein, Glycosyl transferase, PAC2 family protein and Phosphate-ABC- transporter substrate-binding protein) are also largely specific for these genera of *Bifidobacteriales*. Sequence information for these CSIs is provided in Supplementary Figures S36–S39 and their characteristics are summarized in Table 3. Interestingly, the CSIs in the Glycosyl transferase and PAC2 family proteins are also commonly shared by *G. vaginalis*.

**Figure 5:**
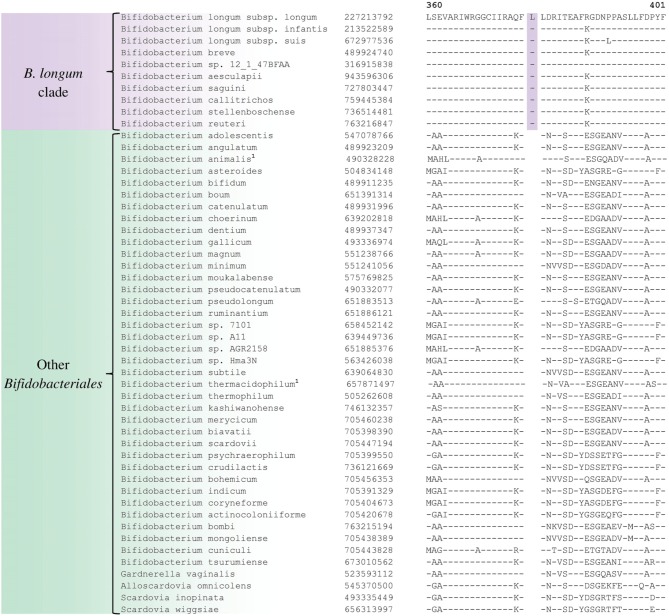
**Partial sequence alignment of phosphogluconate dehydrogenase showing a 1 aa insertion that is specific for the *B. longum* cluster**.

A number of distinct clusters within the genus *Bifidobacterium* are consistently observed in different phylogenetic studies including in the phylogenetic trees constructed in this work (Figure 1). A number of CSIs identified in our work serve to distinguish some of the *Bifidobacterium* clusters. Three of the identified CSIs are specific for the *B. longum* group and sequence information for one of these CSIs, consisting of a 1 aa insertion in the phosphogluconate dehydrogenase, is shown in Figure 5. Sequence information for the other 2 CSIs that are also specific for a subgroup of species from the *B. longum* clade are presented in Supplementary Figures S40, S41 and their characteristics are summarized in Table 3. One additional CSI consisting of a 1 aa insertion in transketolase protein is specifically shared by members of the *B. longum, B. bifidum*, and *B. adolescentis* clades. Members of these clusters group together in phylogenetic trees and the shared presence of this CSI supports the view that that the members of these taxa are more closely and specifically related to each other.

**Figure 6:**
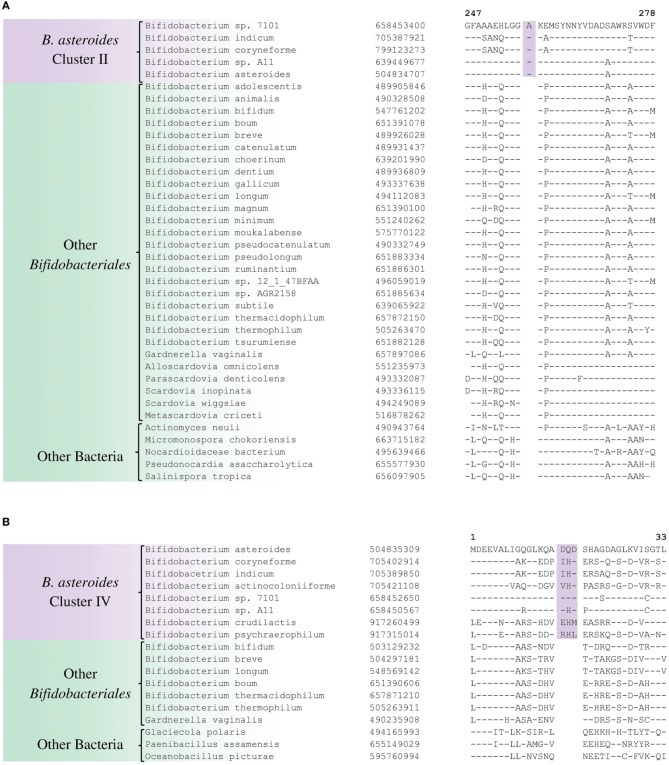
**Conserved signature indels that are specific for the *B. asteroides*-related clades of the *Bifidobacteriales*. (A)** Partial sequence alignment of the purine biosynthesis protein purH showing a 1 aa insertion which is specific for the *B. asteroides* cluster II species in the protein tree (Figure 1); **(B)** Excerpt from sequence alignment of the protein 5′'-methylthioadenosine nucleosidase showing a 3 aa insertion that is specific for the *B. asteroides*-related cluster IV in the protein tree.

The members of the *B. asteroides* cluster forms the deepest branching group within the genus *Bifidobacterium*. A number of CSIs identified in this study are specific for group of species, which are either part of the *B. asteroides* clade or related to this clade. The *B. asteroides* clade is demarcated as being made up of the species *B. asteroides, B. indicum, B. coryneforme*, and *B. actinocoloniiforme* species (marked cluster III in Figure 1) (Lugli et al., 2014; Sun et al., 2015). Surprisingly, in our work no CSI was identified that was commonly shared by all of the species from this clade. However, our work identified four CSIs for a cluster (cluster II) comprising of all of other species from the *B. asteroides* clade, except *B. actinocoliniiforme*, which shows the deepest branching within this clade. One example of a CSI specific for members of the *B. asteroides* cluster II consisting of 1 aa insertion in the purine biosynthesis protein purH is shown in Figure 6A. Sequence information for three other CSIs that are also specific for the *B. asteroides* group is presented in Supplementary Figures S43–S45. In our phylogenetic trees as well as in different identified signatures, two *Bifidobacterium* spp. strains A11 and 7101, isolated from honey bee guts (Anderson et al., 2013), also consistently group with the *B. asteroides*. Two CSIs identified in our work are specifically shared by *B. asteroides* and the *Bifidobacterium* sp. A11 and *Bifidobacterium* sp.7101 (referred to as *B. asteroides* cluster I) providing additional evidence of the close relationship of these *Bifidobacterium* strains to the *B. asteroides*. Sequence information for these CSIs is presented in Supplementary Figure S46. Lastly, one additional CSI identified in this work, consisting of a 3 aa insertion in a conserved region of the protein 5'-methylthioadenosine nucleosidase, is commonly shared by all the members of the *B. asteroides* as well as by *B. crudilactis* and *B. psychaerophilum*. The latter two species form a deeper branching cluster that appears to be specifically related to the *B. asteroides* clade in the tree based on concatenated protein sequences (marked as *B. asteroides* cluster IV in Figure 1). The shared presence of this CSI by the *B. asteroides* clade and *B. crudilactis* and *B. psychaerophilum* support the inference that these species are specifically related to the *B. asteroides* clade.

In addition to the described CSIs, BLAST searches on the protein sequences of *B. asteroides* have also identified 5 CSPs, whose homologs are specifically present in the members of *B. asteroides* group of species. Information for these CSPs is also presented in Table 2. Of these CSPs, three CSPs are specific for the commonly described *B. asteroides* clade (Cluster III in Figure 1), whereas the remaining two are specific for the clusters I and II.

## Discussion

Members of the order *Bifidobacteriales* are one of the main groups within bacteria where several members exhibit health-promoting probiotic effects on humans (Biavati et al., 2000; Biavati and Mattarelli, 2006; Ventura et al., 2007b, 2009a; Cronin et al., 2011; Turroni et al., 2011). Other *Bifidobacteriales* species are also responsible for implicated in the development of dental caries as well as bacterial vaginosis and urinary tract infections (Bradshaw et al., 2006; Mantzourani et al., 2009; Ventura et al., 2009b; Kenyon and Osbak, 2014). However, very little is known at present concerning the genetic or biochemical characteristics of these bacteria that mediate their beneficial or pathogenic effects. In the present work, we have carried out detailed phylogenetic and comparative analyses of protein sequences from the genomes of *Bifidobacteriales* species to examine in depth their evolutionary relationships and also to identify molecular markers that are unique to these bacteria at multiple phylogenetic levels. Based on a robust and comprehensive phylogenetic tree for the *Bifidobacteriales* species based on 614 core proteins from the sequenced genomes, the following inferences regarding the evolutionary relationships among the *Bifidobacteriales* species could be made. (i) The sequenced *Bifidobacteriales* species appear to form two main clusters, a deeper clade consisting of the *Scardovia*-related genera (viz. *Scardovia, Parascardovia* and *Alloscardovia*) and another cluster grouping together *Bifidobacterium* and *Gardnerella* genera. (ii) *Gardnerella vaginalis* rather than branching separately is found to consistently branch in between different *Bifidobacterium* species. (iii) Within *Bifidobacterium* species, a number of distinct clusters, referred to as the *B. asteroides, B. pseudolongum, B. longum, B. bifidum, B. adolescentis, B. pullorum*, and *B. boum* groups, are observed as described in earlier work (Lugli et al., 2014; Sun et al., 2015). Of these clusters, the *B. asteroides* group forms the deepest branching lineage within the *Bifidobacterium* (Bottacini et al., 2012; Lugli et al., 2014; Sun et al., 2015).

The present work also identified large number of novel molecular signatures in the forms of CSIs and CSPs, which are specific characteristics of the members of the order *Bifidobacteriales* at multiple phylogenetic levels. Of these signatures, 32 CSIs and 10 CSPs are specific for the entire order *Bifidobacteriales*. The identified *Bifidobacteriales*-specific CSIs are present in assorted widely distributed proteins carrying out wide variety of cellular functions. All of the 10 *Bifidobacteriales*-specific CSPs are proteins of unknown functions. Given the specificity of these CSIs and CSPs for the *Bifidobacteriales*, the genetic changes leading to these molecular characteristics have likely occurred in a common ancestor of the *Bifidobacteriales* (Gao and Gupta, 2005, 2012). Additionally, our analyses have also identified many other molecular signatures (CSIs and CSPs), which independently support the existence of a number of clades of bifidobacteria that are consistently observed in phylogenetic trees. The clades identified by these molecular signatures include, (i) a clade encompassing the genera *Scardovia, Parascardovia* and *Alloscardovia*, (ii) signatures that are commonly shared by *Bifidobacterium* and *Gardnerella* species to the exclusion of other bifidobacteria, and (iii) signatures demarcating specific clusters of *B. asteroides*- or *B. longum*- related species.

The order *Bifidobacteriales* presently contains a single family, *Bifidobacteriaceae*. Based upon the results of phylogenomic studies and identified molecular signatures, it appears that the members of this order could be divided into two family-level groups, one comprising of the *Scardovia*-related genera (viz. *Scarodivia, Parascardovia*, and *Alloscardovia*) and the other consisting of the genera *Bifidobacterium* and *Gardnerella*. However, genome sequence information for members of several newly described *Scardovia*-related genera (viz. *Aeriscardovia, Neoscardovia*, and *Pseudoscardovia*), is lacking at present (Simpson et al., 2004; García-Aljaro et al., 2012; Killer et al., 2013). In future studies, depending upon whether the species from these genera branch with the *Scardovia*-clade and their sharing of the molecular signatures specific for this clade, the possibility of dividing the order *Bifidobacteriales* into two or more families could be considered.

The genus *Bifidobacterium*, which is comprised of 49 species and subspecies, contains most of the recognized taxa within the order *Bifidobacteriales*. Although earlier phylogenetic studies have consistently observed 6–7 distinct clusters of *Bifidobacterium* species (Ventura et al., 2006, 2007b; Turroni et al., 2011; Lugli et al., 2014; Sun et al., 2015), due to lack of any other distinguishing characteristics, no attempt has been made to formally recognize any of these clusters. In our work, we have identified a number of molecular signatures that are either completely or largely specific for the members of two of these clusters (viz. the *B. asteroides* and *B. longum* groups). Of these clusters, the distinctness of the *B. asteroides* group (comprising of the species *B. asteroides, B. indicum, B. coryneforme, B. actinocoloniiforme, B*. sp. A11, and *B*. sp. 7101) which forms the deepest branching lineage within the *Bifidobacterium*, is supported by 2 CSIs and 4 CSPs that are uniquely shared by most of the members of this clade. Further, most of the species which are part of the *B. asteroides* clade have been isolated from the gastrointestinal tract of honey bees, and unlike other bifidobacteria, they are also capable of carrying out respiratory metabolism (Killer et al., 2010, 2011; Bottacini et al., 2012; Lugli et al., 2014; Sun et al., 2015). All of these characteristics indicate that the members of the *B. asteroides* clade are a good candidate for recognition as a distinct genus level taxon within the order *Bifidobacteriales*.

**Figure 7:**
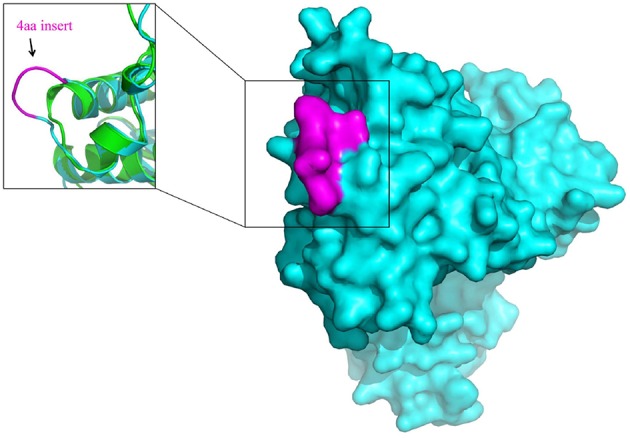
**Surface representation of the homology model of Elongation factor Tu (EF-Tu) from *B. longum* (Cyan)**. The conserved 4 aa insert which is located on the surface of the EF-Tu is shown in magenta. A superposition of the homology model of the *B. longum* homolog of EF-Tu (Cyan) with the *E. coli* homolog of EF-Tu of (PDB ID: 3U6K) (Green) shows that the conserved 4 aa insert forms a surface loop on the protein.

The molecular markers for the order *Bifidobacteriales* and some of its clades, in addition to their utility for taxonomic and diagnostic studies (Ahmod et al., 2011; Gupta, 2014; Wong et al., 2014), also provide important new tools for genetic and biochemical studies. Earlier work on a number of CSIs in the Hsp60 and Hsp70 proteins has established that both large and small CSIs in conserved proteins are essential for the group of organisms in which they are found (Singh and Gupta, 2009; Gupta, 2016b). Removal of these CSIs, or any significant change in them, was shown to be incompatible with the cellular growth of the CSI-containing organisms. Thus, the identified CSIs are predicted to play essential role in the organisms in which they are found. Structural studies on several studied CSIs show that the sequences corresponding to them are present in the surface loops of the proteins (Singh and Gupta, 2009; Gupta and Khadka, 2016). Limited structural work on some of the *Bifidobacteriales*-specific CSIs that we have carried out also shows that these CSIs are located in the surface loops of the proteins. One example of the structural location of a *Bifidobacteriales*- specific CSIs is illustrated in Figure 7. In this case, a homology model of protein synthesis elongation factor Tu from *B. longum* was created to determine the location of the 4 aa *Bifidobacteriales*-specific CSI found in this protein. A structural comparison of the EF-Tu from *B. longum* and *E. coli* shown in Figure 7 reveals that the CSI in the *B. longum* homolog is present in the protein surface loop within the GTPase domain of EF-Tu. The surface loops in proteins play important role in mediating protein-protein or protein-ligand interactions and it is expected that the identified CSIs are involved in mediating novel interactions that are specific and essential for the CSI-containing organisms (Akiva et al., 2008; Hashimoto and Panchenko, 2010). Similar to the CSIs in the EF-Tu protein, our work has identified numerous other CSIs in different essential proteins, which are specific for the *Bifidobacteriales* species. Functional studies on proteins harboring these CSIs provide an important means for discovering novel biochemical characteristics that are unique to either all *Bifidobacteriales* or specific clades of these bacteria, and which could possibly also provide useful insights into the growth-promoting as well as pathogenic effects of some of these bacteria.

## Author contributions

GZ, BG, MA, BK carried out comparative analyses of the bifidobacteriales genomes to identify signatures reported here. ZG and MA constructed phylogenetic trees and BK carried out homology modeling of the protein sequences. BG, MA, and RG were responsible for the writing and editing of the manuscript. All of the work was carried out under the direction of RG.

### Conflict of interest statement

The authors declare that the research was conducted in the absence of any commercial or financial relationships that could be construed as a potential conflict of interest.

## References

[B1] Ahmod N. Z. Gupta R. S. Shah H. N. (2011). Identification of a *Bacillus anthracis* specific indel in the yeaC gene and development of a rapid pyrosequencing assay for distinguishing *B. anthracis* from the *B. cereus* group. J. Microbiol. Methods 87, 278–285. 10.1016/j.mimet.2011.08.015 21907250

[B2] Ajawatanawong P. Baldauf S. L. (2013). Evolution of protein indels in plants, animals and fungi. BMC Evol. Biol. 13:140. 10.1186/1471-2148-13-140 23826714 PMC3706215

[B3] Akiva E. Itzhaki Z. Margalit H. (2008). Built-in loops allow versatility in domain-domain interactions: lessons from self-interacting domains. Proc. Natl. Acad. Sci. U.S.A. 105, 13292–13297. 10.1073/pnas.0801207105 18757736 PMC2533183

[B4] Altschul S. F. Madden T. L. Schaffer A. A. Zhang J. Zhang Z. Miller W. (1997). Gapped BLAST and PSI-BLAST: a new generation of protein databases search programs. Nucleic Acids Res. 25, 3389–3402. 10.1093/nar/25.17.3389 9254694 PMC146917

[B5] Alves P. Castro J. Sousa C. Cereija T. B. Cerca N. (2014). *Gardnerella vaginalis* outcompetes 29 other bacterial species isolated from patients with bacterial vaginosis, using in an *in vitro* biofilm formation model. J. Infect. Dis. 210, 593–596. 10.1093/infdis/jiu131 24596283

[B6] Anderson K. E. Johansson A. Sheehan T. H. Mott B. M. Corby-Harris V. Johnstone L.. (2013). Draft genome sequences of two *Bifidobacterium* sp. from the honey bee (*Apis mellifera*). Gut Pathog. 5:42. 10.1186/1757-4749-5-42 24350840 PMC3878406

[B7] Biavati B. (2012). Family I *Bifidobacteriaceae* Stackebrandt, Rainey and Ward-Rainey 1997, 487^VP^, in Bergey's Manual of Systematic Bacteriology, Vol. 5, The Actinobacteria, eds Whitman W. Goodfellow M. Kampfer P. Busse H. J. Trujillo M. E. Ludwig W. (New York, NY: Springer), 171.

[B8] Biavati B. Mattarelli P. (2012). Genus I *Bifidobacterium* Orla-Jensen 1924, 472^AL^, in Bergey's Manual of Systematic Bacteriology, Vol. 5, The Actinobacteria, eds Whitman W. Goodfellow M. Kampfer P. Busse H. J. Trujillo M. E. Ludwig W. (New York, NY: Springer), 171–206.

[B9] Biavati B. Mattarelli P. (2006). The family *Bifidobacteriaceae*, in The Prokaryotes: An Evolving Electronic Resource for the Microbiological Community, eds Dworkin M. Falkow S. Rosenberg E. Schleifer K. H. Stackebrandt E. (New York, NY: Springer-Verlag), 322–382. 10.1007/0-387-30743-5_17

[B10] Biavati B. Vescovo M. Torriani S. Bottazzi V. (2000). Bifidobacteria: histroy, ecology, physiology and applications. Ann. Microbiol. 50, 117–131.

[B11] Bottacini F. Medini D. Pavesi A. Turroni F. Foroni E. Riley D.. (2010). Comparative genomics of the genus *Bifidobacterium*. Microbiology 156, 3243–3254. 10.1099/mic.0.039545-0 20634238

[B12] Bottacini F. Milani C. Turroni F. Sanchez B. Foroni E. Duranti S.. (2012). *Bifidobacterium* asteroides PRL2011 genome analysis reveals clues for colonization of the insect gut. PLoS ONE 7:e44229. 10.1371/journal.pone.0044229 23028506 PMC3447821

[B13] Bradshaw C. S. Tabrizi S. N. Fairley C. K. Morton A. N. Rudland E. Garland S. M (2006). The association of *Atopobium vaginae* and *Gardnerella vaginalis* with bacterial vaginosis and recurrence after oral metronidazole therapy. J. Infect. Dis. 194, 828–836. 10.1086/506621 16941351

[B14] Castresana J. (2000). Selection of conserved blocks from multiple alignments for their use in phylogenetic analysis. Mol. Biol. Evol. 17, 540–552. 10.1093/oxfordjournals.molbev.a026334 10742046

[B15] Cole J. R. Wang Q. Fish J. A. Chai B. McGarrell D. M. Sun Y.. (2014). Ribosomal Database Project: data and tools for high throughput rRNA analysis. Nucleic Acids Res. 42, D633–D642. 10.1093/nar/gkt1244 24288368 PMC3965039

[B16] Cronin M. Ventura M. Fitzgerald G. F. van Sinderen D. (2011). Progress in genomics, metabolism and biotechnology of bifidobacteria. Int. J. Food Microbiol. 149, 4–18. 10.1016/j.ijfoodmicro.2011.01.019 21320731

[B17] Edgar R. C. (2010). Search and clustering orders of magnitude faster than BLAST. Bioinformatics 26, 2460–2461. 10.1093/bioinformatics/btq461 20709691

[B18] Ferrario C. Milani C. Mancabelli L. Lugli G. A. Turroni F. Duranti S.. (2015). A genome-based identification approach for members of the genus *Bifidobacterium*. FEMS Microbiol. Ecol. 91:fiv009. 10.1093/femsec/fiv009 25764568

[B19] Gao B. Gupta R. S. (2005). Conserved indels in protein sequences that are characteristic of the phylum *Actinobacteria*. Int. J. Syst. Evol. Microbiol. 55, 2401–2412. 10.1099/ijs.0.63785-0 16280504

[B20] Gao B. Gupta R. S. (2012). Phylogenetic framework and molecular signatures for the main clades of the phylum Actinobacteria. Microbiol. Mol. Biol. Rev. 76, 66–112. 10.1128/MMBR.05011-11 22390973 PMC3294427

[B21] Gao B. Parmanathan R. Gupta R. S. (2006). Signature proteins that are distinctive characteristics of *Actinobacteria* and their subgroups. Antonie Van Leeuwenhoek 90, 69–91. 10.1007/s10482-006-9061-2 16670965

[B22] García-Aljaro C. Ballesté E. Rosselló-Móra R. Cifuentes A. Richter M. Blanch A. R. (2012). *Neoscardovia arbecensis* gen. nov., sp. nov., isolated from porcine slurries. Syst. Appl. Microbiol. 35, 374–379. 10.1016/j.syapm.2012.06.007 22824582

[B23] Guindon S. Dufayard J. F. Lefort V. Anisimova M. Hordijk W. Gascuel O. (2010). New algorithms and methods to estimate maximum-likelihood phylogenies: assessing the performance of PhyML 3.0. Syst. Biol. 59, 307–321. 10.1093/sysbio/syq010 20525638

[B24] Gupta R. S. (1998). Protein phylogenies and signature sequences: a reappraisal of evolutionary relationships among archaebacteria, eubacteria, and eukaryotes. Microbiol. Mol. Biol. Rev. 62, 1435–1491. 9841678 10.1128/mmbr.62.4.1435-1491.1998 PMC98952

[B25] Gupta R. S. (2010). Applications of conserved indels for understanding microbial phylogeny, in Molecular Phylogeny of Microorganisms, eds Oren A. Papke R. T. (Norfolk: Caister Academic Press), 135–150.

[B26] Gupta R. S. (2014). Identification of conserved indels that are useful for classification and evolutionary studies, in Bacterial Taxonomy, Methods in Microbiology, Vol. 41, eds Goodfellow M. Sutcliffe I. C. Chun J. (London: Elsevier), 153–182. 10.1016/bs.mim.2014.05.003

[B27] Gupta R. S. (2016a). Editorial: applications of genome sequences for discovering characteristics that are unique to different groups of organisms and provide insights into evolutionary relationships. Front. Genet. 7:27. 10.3389/fgene.2016.00027 26925098 PMC4759622

[B28] Gupta R. S. (2016b). Impact of genomics on the understanding of microbial evolution and classification: the importance of Darwin's views on classification. FEMS Microbiol. Rev. [Epub ahead of print]. 10.1093/femsre/fuw011 27279642

[B29] Gupta R. S. Chander P. George S. (2013a). Phylogenetic framework and molecular signatures for the class Chloroflexi and its different clades; proposal for division of the class *Chloroflexia* class. nov. [corrected] into the suborder *Chloroflexineae* subord. nov., consisting of the emended family Oscillochloridaceae and the family *Chloroflexaceae* fam. nov., and the suborder *Roseiflexineae subord*. nov., containing the family Roseiflexaceae fam. nov. Antonie van Leeuwenhoek 103, 99–119. 10.1007/s10482-012-9790-3 22903492

[B30] Gupta R. S. Chen W. J. Adeolu M. Chai Y. (2013b). Molecular signatures for the class *Coriobacteriia* and its different clades; Proposal for division of the class *Coriobacteriia* into the emended order *Coriobacteriales*, containing the emended family *Coriobacteriaceae* and *Atopobiaceae* fam. nov., and *Eggerthellales* ord. nov., containing the family *Eggerthellaceae* fam. nov. Int. J. Syst. Evol. Microbiol. 63, 3379–3397. 10.1099/ijs.0.048371-0 23524353

[B31] Gupta R. S. Griffiths E. (2002). Critical issues in bacterial phylogeny. Theor. Popul. Biol. 61, 423–434. 10.1006/tpbi.2002.1589 12167362

[B32] Gupta R. S. Khadka B. (2016). Evidence for the presence of key chlorophyll-biosynthesis-related proteins in the genus Rubrobacter (Phylum Actinobacteria) and its implications for the evolution and origin of photosynthesis. Photosyn. Res. 127, 201–218. 10.1007/s11120-015-0177-y 26174026

[B33] Gupta R. S. Naushad S. Fabros R. Adeolu M. (2016). A phylogenomic reappraisal of family-level divisions within the class *Halobacteria*: proposal to divide the order Halobacteriales into the families *Halobacteriaceae, Haloarculaceae* fam. nov., and *Halococcaceae* fam. nov., and the order Haloferacales into the families, *Haloferacaceae* and *Halorubraceae* fam nov. Antonie van Leeuwenhoek 109, 565–587. 10.1007/s10482-016-0660-2 26837779

[B34] Harris J. K. Kelley S. T. Spiegelman G. B. Pace N. R. (2003). The genetic core of the universal ancestor. Genome Res. 13, 407–412. 10.1101/gr.652803 12618371 PMC430263

[B35] Hashimoto K. Panchenko A. R. (2010). Mechanisms of protein oligomerization, the critical role of insertions and deletions in maintaining different oligomeric states. Proc. Natl. Acad. Sci. U.S.A. 107, 20352–20357. 10.1073/pnas.1012999107 21048085 PMC2996646

[B36] Huys G. Vancanneyt M. D'Haene K. Falsen E. Wauters G. Vandamme P. (2007). *Alloscardovia omnicolens* gen. nov., sp nov., from human clinical samples. Int. J. Syst. Evol. Microbiol. 57, 1442–1446. 10.1099/ijs.0.64812-0 17625172

[B37] Jeanmougin F. Thompson J. D. Gouy M. Higgins D. G. Gibson T. J. (1998). Multiple sequence alignment with Clustal x. Trends Biochem. Sci. 23, 403–405. 10.1016/S0968-0004(98)01285-7 9810230

[B38] Jian W. Y. Dong X. Z. (2002). Transfer of *Bifidobacterium* incipinatum and *Bifidobacterium* denticolens to *Scardovia inopinata* gen. nov., comb. nov., and Parascardovia denticolens gen. nov., comb. nov., respectively. Int. J. Syst. Evol. Microbiol. 52, 809–812. 10.1099/00207713-52-3-809 12054242

[B39] Kenyon C. R. Osbak K. (2014). Recent progress in understanding the epidemiology of bacterial vaginosis. Curr. Opin. Obstet. Gynecol. 26, 448–454. 10.1097/GCO.0000000000000112 25304606

[B40] Killer J. Kopecný J. Mrázek J. Koppová I. Havlík J. Benada O.. (2011). *Bifidobacterium actinocoloniiforme* sp. nov. and *Bifidobacterium bohemicum* sp nov., from the bumblebee digestive tract. Int. J. Syst. Evol. Microbiol. 61, 1315–1321. 10.1099/ijs.0.022525-0 20656822

[B41] Killer J. Kopecný J. Mrázek J. Rada V. Dubná S. Marounek M. (2010). Bifidobacteria in the digestive tract of bumblebees. Anaerobe 16, 165–170. 10.1016/j.anaerobe.2009.07.007 19651224

[B42] Killer J. Mrazek J. Bunesova V. Havlik J. Koppova I. Benada O.. (2013). *Pseudoscardovia suis* gen. nov., sp. nov., a new member of the family *Bifidobacteriaceae* isolated from the digestive tract of wild pigs *(Sus scrofa)*. Syst. Appl. Microbiol. 36, 11–16. 10.1016/j.syapm.2012.09.001 23122702

[B43] Le S. Q. Gascuel O. (2008). An improved general amino acid replacement matrix. Mol. Biol. Evol. 25, 1307–1320. 10.1093/molbev/msn067 18367465

[B44] Leahy S. C. Higgins D. G. Fitzgerald G. F. van Sinderen D. (2005). Getting better with bifidobacteria. J. Appl. Microbiol. 98, 1303–1315. 10.1111/j.1365-2672.2005.02600.x 15916644

[B45] Lugli G. A. Milani C. Turroni F. Duranti S. Ferrario C. Viappiani A.. (2014). Investigation of the evolutionary development of the genus *Bifidobacterium* by comparative genomics. Appl. Environ. Microbiol. 80, 6383–6394. 10.1128/AEM.02004-14 25107967 PMC4178631

[B46] Mantzourani M. Fenlon M. Beighton D. (2009). Association between *Bifidobacteriaceae* and the clinical severity of root caries lesions. Oral Microbiol. Immunol. 24, 32–37. 10.1111/j.1399-302X.2008.00470.x 19121067

[B47] Mattarelli P. Holzapfel W. Franz C. M. A. P. Endo A. Felis G. E. Hammes W.. (2014). Recommended minimal standards for description of new taxa of the genera *Bifidobacterium*, Lactobacillus and related genera. Int. J. Syst. Evol. Microbiol. 64, 1434–1451. 10.1099/ijs.0.060046-0 24706714

[B48] Milani C. Lugli G. A. Duranti S. Turroni F. Bottacini F. Mangifesta M.. (2014). Genomic encyclopedia of type strains of the genus *Bifidobacterium*. Appl. Environ. Microbiol. 80, 6290–6302. 10.1128/AEM.02308-14 25085493 PMC4178644

[B49] Miyake T. Watanabe K. Watanabe T. Oyaizu H. (1998). Phylogenetic analysis of the genus *Bifidobacterium* and related genera based on 16S rDNA sequences. Microbiol. Immunol. 42, 661–667. 10.1111/j.1348-0421.1998.tb02337.x 9858460

[B50] Parte A. C. (2014). LPSN-list of prokaryotic names with standing in nomenclature. Nucleic Acids Res. 42, D613–D616. 10.1093/nar/gkt1111 24243842 PMC3965054

[B51] Price M. N. Dehal P. S. Arkin A. P. (2010). FastTree 2–approximately maximum-likelihood trees for large alignments. PLoS ONE 5:e9490. 10.1371/journal.pone.0009490 20224823 PMC2835736

[B52] Pruesse E. Peplies J. Glöckner F. O. (2012). SINA: accurate high-throughput multiple sequence alignment of ribosomal RNA genes. Bioinformatics 28, 1823–1829. 10.1093/bioinformatics/bts252 22556368 PMC3389763

[B53] Rokas A. Holland P. W. (2000). Rare genomic changes as a tool for phylogenetics. Trends Ecol. Evol. (Amst). 15, 454–459. 10.1016/S0169-5347(00)01967-4 11050348

[B54] Sali A. Blundell T. L. (1993). Comparative protein modelling by satisfaction of spatial restraints. J. Mol. Biol. 234, 779–815. 10.1006/jmbi.1993.1626 8254673

[B55] Shen M. Y. Sali A. (2006). Statistical potential for assessment and prediction of protein structures. Protein Sci. 15, 2507–2524. 10.1110/ps.062416606 17075131 PMC2242414

[B56] Sievers F. Wilm A. Dineen D. Gibson T. J. Karplus K. Li W.. (2011). Fast, scalable generation of high-quality protein multiple sequence alignments using Clustal Omega. Mol. Syst. Biol. 7, 539. 10.1038/msb.2011.75 21988835 PMC3261699

[B57] Simpson P. J. Ross R. P. Fitzgerald G. F. Stanton C. (2004). *Bifidobacterium psychraerophilum* sp. nov. and *Aeriscardovia aeriphila* gen. nov., sp. nov., isolated from a porcine caecum. Int. J. Syst. Evol. Microbiol. 54, 401–406. 10.1099/ijs.0.02667-0 15023951

[B58] Singh B. Gupta R. S. (2009). Conserved inserts in the Hsp60 (GroEL) and Hsp70 (DnaK) proteins are essential for cellular growth. Mol. Genet. Genomics 281, 361–373. 10.1007/s00438-008-0417-3 19127371

[B59] Smith S. M. Ogbara T. Eng R. H. K. (1992). Involvement of *Gardnerella-vaginalis* in urinary-tract infections in men. J. Clin. Microbiol. 30, 1575–1577. 1624577 10.1128/jcm.30.6.1575-1577.1992 PMC265332

[B60] Stamatakis A. (2014). RAxML version 8: a tool for phylogenetic analysis and post-analysis of large phylogenies. Bioinformatics 30, 1312–1313. 10.1093/bioinformatics/btu033 24451623 PMC3998144

[B61] Storms V. Vandamme P. (2012). Genus IV gardnerella greenwood and pickett 1980, 176^VP^, in Bergey's Manual of Systematic Bacteriology, Vol. 5, The Actinobacteria, eds Whitman W. Goodfellow M. Kampfer P. Busse H. J. Trujillo M. E. Ludwig W. (New York, NY: Springer), 208–211.

[B62] Sun Z. H. Zhang W. Y. Guo C. Y. Yang X. W. Liu W. J. Wu Y.. (2015). Comparative genomic analysis of 45 type strains of the genus *Bifidobacterium*: a snapshot of its genetic diversity and evolution. PLoS ONE 10:e117912. 10.1371/journal.pone.0117912 25658111 PMC4319941

[B63] Talavera G. Castresana J. (2007). Improvement of phylogenies after removing divergent and ambiguously aligned blocks from protein sequence alignments. Syst. Biol. 56, 564–577. 10.1080/10635150701472164 17654362

[B64] Tamura K. Stecher G. Peterson D. Filipski A. Kumar S. (2013). MEGA6: molecular evolutionary genetics analysis version 6.0. Mol. Biol. Evol. 30, 2725–2729. 10.1093/molbev/mst197 24132122 PMC3840312

[B65] Toh H. Yamazaki Y. Tashiro K. Kawarai S. Oshima K. Nakano A.. (2015). Draft genome sequence of *Bifidobacterium* aesculapii DSM 26737T, isolated from feces of baby common marmoset. Genome Announc. 3:e01463–15. 10.1128/genomeA.01463-15 26659692 PMC4675957

[B66] Turroni F. Bottacini F. Foroni E. Mulder I. Kim J. H. Zomer A.. (2010). Genome analysis of *Bifidobacterium bifidum* PRL2010 reveals metabolic pathways for host-derived glycan foraging. Proc. Natl. Acad. Sci. U.S.A. 107, 19514–19519. 10.1073/pnas.1011100107 20974960 PMC2984195

[B67] Turroni F. van Sinderen D. Ventura M. (2009). *Bifidobacteria*: from ecology to genomics. Front. Biosci. 14, 4673–4684. 10.2741/3559 19273381

[B68] Turroni F. van Sinderen D. Ventura M. (2011). Genomics and ecological overview of the genus *Bifidobacterium*. Int. J. Food Microbiol. 149, 37–44. 10.1016/j.ijfoodmicro.2010.12.010 21276626

[B69] Ventura M. Canchaya C. Del Casale A. Dellaglio F. Neviani E. Fitzgerald G. F.. (2006). Analysis of bifidobacterial evolution using a multilocus approach. Int. J. Syst. Evol. Microbiol. 56, 2783–2792. 10.1099/ijs.0.64233-0 17158978

[B70] Ventura M. Canchaya C. Fitzgerald G. F. Gupta R. S. van Sinderen D. (2007a). Genomics as a means to understand bacterial phylogeny and ecological adaptation: the case of bifidobacteria. Antonie Van Leeuwenhoek 91, 351–372. 10.1007/s10482-006-9122-6 17072531

[B71] Ventura M. Canchaya C. Tauch A. Chandra G. Fitzgerald G. F. Chater K. F.. (2007b). Genomics of *Actinobacteria*: tracing the evolutionary history of an ancient phylum. Microbiol. Mol. Biol. Rev. 71, 495–548. 10.1128/MMBR.00005-07 17804669 PMC2168647

[B72] Ventura M. Canchaya C. Zink R. Fitzgerald G. F. van Sinderen D. (2004). Characterization of the groEL and groES loci in *Bifidobacterium breve* UCC 2003: genetic, transcriptional, and phylogenetic analyses. Appl. Environ. Microbiol. 70, 6197–6209. 10.1128/AEM.70.10.6197-6209.2004 15466567 PMC522111

[B73] Ventura M. O'Flaherty S. Claesson M. J. Turroni F. Klaenhammer T. R. van Sinderen D.. (2009a). Genome-scale analyses of health-promoting bacteria: probiogenomics. Nat. Rev. Microbiol. 7, 61–71. 10.1038/nrmicro2047 19029955

[B74] Ventura M. Turroni F. Zomer A. Foroni E. Giubellini V. Bottacini F.. (2009b). The *Bifidobacterium dentium* Bd1 genome sequence reflects its genetic adaptation to the human oral cavity. PLoS Genet. 5:e1000785. 10.1371/journal.pgen.1000785 20041198 PMC2788695

[B75] Ventura M. Zink R. (2003). Comparative sequence analysis of the tuf and recA genes and restriction fragment length polymorphism of the internal transcribed spacer region sequences supply additional tools for discriminating *Bifidobacterium lactis* from *Bifidobacterium animalis*. Appl. Environ. Microbiol. 69, 7517–7522. 10.1128/AEM.69.12.7517-7522.2003 14660406 PMC310005

[B76] Whelan S. Liò P. Goldman N. (2001). Molecular phylogenetics: state-of-the-art methods for looking into the past. Trends Genet. 17, 262–272. 10.1016/S0168-9525(01)02272-7 11335036

[B77] Wong S. Y. Paschos A. Gupta R. S. Schellhorn H. E. (2014). Insertion/deletion-based approach for the detection of *Escherichia coli* O157:H7 in freshwater environments. Environ. Sci. Technol. 48, 11462–11470. 10.1021/es502794h 25166281

[B78] Xu D. Zhang Y. (2011). Improving the physical realism and structural accuracy of protein models by a two-step atomic-level energy minimization. Biophys. J. 101, 2525–2534. 10.1016/j.bpj.2011.10.024 22098752 PMC3218324

[B79] Yarza P. Richter M. Peplies J. Euzeby J. Amann R. Schleifer K. H.. (2008). The All-Species Living Tree project: a 16S rRNA-based phylogenetic tree of all sequenced type strains. Syst. Appl. Microbiol. 31, 241–250. 10.1016/j.syapm.2008.07.001 18692976

[B80] Yilmaz P. Parfrey L. W. Yarza P. Gerken J. Pruesse E. Quast C.. (2014). The SILVA and “All-species Living Tree Project (LTP)” taxonomic frameworks. Nucleic Acids Res. 42, D643–D648. 10.1093/nar/gkt1209 24293649 PMC3965112

[B81] Zhi X. Y. Li W. J. Stackebrandt E. (2009). An update of the structure and 16S rRNA gene sequence-based definition of higher ranks of the class *Actinobacteria*, with the proposal of two new suborders and four new families and emended descriptions of the existing higher taxa. Int. J. Syst. Evol. Microbiol. 59, 589–608. 10.1099/ijs.0.65780-0 19244447

