# Supplementary material for: Phylogenomic Analyses and Comparative Studies on Genomes of the Bifidobacteriales: Identification of Molecular Signatures Specific for the Order Bifidobacteriales and Its Different Subclades
Source: Front Microbiol. 2016 Jun 27;7:978. doi: 10.3389/fmicb.2016.00978 (PMC4921777; doi:10.3389/fmicb.2016.00978)
Supplement: Supplementary file 1 [file DataSheet1.PDF]

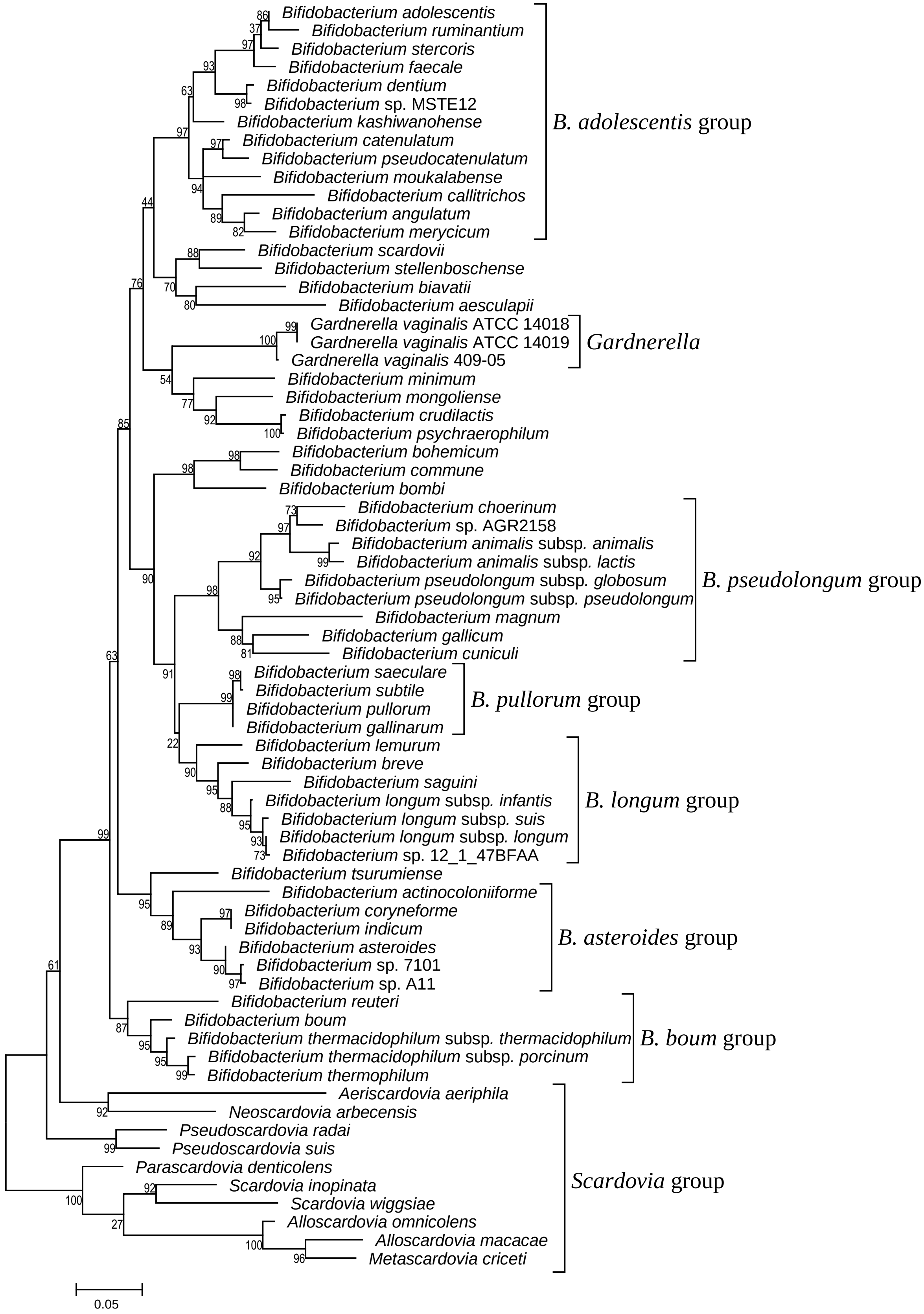

Supplementary figure S1

A maximum-likelihood tree based on the 16S rRNA gene sequences of type strains covering all described species within the order *Bifidobacteriales*. The tree was rooted at the midpoint and bootstrap support values are indicated at nodes. A number of different clades/clusters that are consistently observed in phylogenetic trees are marked.

			31		80
Bifidobacteriales	Bifidobacterium adolescentis	489904111	VMASVGHIRDLAQPSQVPAAEKAK		YGKFGVDIEDGFKPYYIVDGNKKKTV
	Bifidobacterium angulatum	489924281	--------------------D-EH		-------VN---------GAD-R---
	Bifidobacterium animalis	504510055	-L-----------------D----		F------VN---------GPE-----
	Bifidobacterium asteroides	504835434	----------------I--SQ-KA		-------VN----------DK--H--
	Bifidobacterium bifidum	489918302	----------------I--DD--R		F------VD-----------D-----
	Bifidobacterium breve	505251178	--------------------D-P-		F------VN-----------D--R--
	Bifidobacterium catenulatum	489933009	--------------------D-T-		F------V------------------
	Bifidobacterium choerinum	639203254	-L-----------------DQ--R		F------VD-------V-GAD-----
	Bifidobacterium dentium	489934378	------------------S-D---		F------V------------D-----
	Bifidobacterium gallicum	493338498	-L------------------Q-ER		F-R----VN---A-----GSD--H--
	Bifidobacterium longum	494113915	--------------------D---		F------VN-----------D--R--
	Bifidobacterium magnum	551239505	-------------------DD--Q		-------VN---------GTD--R--
	Bifidobacterium minimum	551240839	--------------------K--S		F------VD---E---V-GPE-R---
	Bifidobacterium moukalabense	575769218	------------------SDD--R		-------V------------D-----
	Bifidobacterium pseudocatenulatum	490330843	--------------------D-T-		F------V------------------
	Bifidobacterium pseudolongum	551237663	-L--------------I--DQ--Q		-------VN---------GTD-----
	Bifidobacterium ruminantium	651886928	--------------------D---		--------------------------
	Bifidobacterium subtile	639062571	--------------------Q---		F------VD---A-----GSD-----
	Bifidobacterium thermacidophilum	657871454	-L-----------------SK-D-		F------VN---------GPD-----
	Bifidobacterium thermophilum	505262669	-L-----------------SK-D-		F------VN---E-----GPD-----
	Bifidobacterium tsurumiense	651882004	-------------------GQ-E-		F------VN---E-----GAD-----
	Bifidobacterium actinocoloniiforme	672944897	------------K---I-TSQ-ER		F------VDHD--------D------
	Bifidobacterium aesculapii	943597413	--------------------D---		-------VD-----------D--R--
	Bifidobacterium biavatii	705396040	-------------------SD---		F------V------------D-----
	Bifidobacterium bohemicum	705456372	-K-----------------SK--E		F-R----VNN--------GAD-----
	Bifidobacterium bombi	763215105	-K-------------E----K-E-		F-R----VN---E---V-GPQ-----
	Bifidobacterium boum	705388643	-L------------------K-E-		-------VN---E-----GPD-----
	Bifidobacterium callitrichos	759443576	-------------------D----		F------VD----------A------
	Bifidobacterium coryneforme	799124179	-------------------SQ-KE		--R----VT----------DK-----
	Bifidobacterium cuniculi	705446712	-L-----------------DK--Q		F------VN---T-----GAD-R---
	Bifidobacterium gallinarum	705423499	-------------------SA---		F------V----------GSE--R--
	Bifidobacterium indicum	705390176	-------------------SQ-KE		--R----VT----------DK-----
	Bifidobacterium kashiwanohense	746131381	--------------------D-T-		F------V------------------
	Bifidobacterium merycicum	705459735	----------------I---D-E-		-------V----------GSE-R---
	Bifidobacterium mongoliense	672982973	----------------I---Q-S-		F-R----VD---A---V-GAE-----
	Bifidobacterium psychraerophilum	705397769	--------------------K--Q		F-------D-D-T-------D-----
	Bifidobacterium pullorum	705441790	--------------------D---		F------VD---------GSE-----
	Bifidobacterium reuteri	763217319	--------------------D---		F------VN-----------D--R--
	Bifidobacterium saeculare	705428687	-------------------SA---		F------V----------GSE--R--
	Bifidobacterium saguini	727803497	--------------------D---		F------VN-----------D--R--
	Bifidobacterium scardovii	705447008	--------------------D---		F------V------------D--R--
	Bifidobacterium stellenboschense	736511402	--------------------D-E-		-------VN-----------D--R--
	Bifidobacterium stercoris	673001277	------------------------		--------------------------
	Gardnerella vaginalis	490238861	-------------------SR--A		F------VDH--A---V-GAD-----
	Alloscardovia criceti	516875996	-L--------------I---D--D		------NVN---------GDD-----
	Parascardovia denticolens	493333697	----------------I--ED--R		-------VD---L---V-GKD-----
	Alloscardovia omnicolens	545369570	-L--------------I--SD--E		------NVD----------D------
	Scardovia inopinata	493336463	----------------I---D-E-		-------VD---L-----GND-----
	Scardovia wiggsiae	494252292	---------------E----D-E-		-------VD---L---V-GQD-----
Other Bacteria 2/500	Microbacterium profundi	696575142	-LS---------DKKDI---D--A		---YSI--DN--D---V-SDR-T---
	Microbacterium hydrocarbonoxydans	770664504	-LS---------DKKDI---D--A		---YSI--DN--D---V-SDR-T---
	Actinobaculum massiliense	494059122	-L---------P---EL-ESM-KG	P	F---A--VDGD-E---V-APD-----
	Agrococcus pavilionensis	540322667	-L---------VS-RDL--DL-KG	A	F---A--VDN--A---V-SDE-----
	Arcanobacterium haemolyticum	502935896	-T--I------PK--DL-SDM-KG	P	F---A--V-HN-E---V-NPD---K-
	Austwickia chelonae	493548044	-QS-F------PN--EM--DM-KG	P	--R-A--V-N--D---V--AD--RQ-
	Brevibacterium casei	496838048	-E---------PT--EL--DM-KG	P	----A--VDN--D---R--PG---K-
	Cellulomonas fimi	503535573	-E---------P---EL--DM-KG	P	F---A--VDN--V---V--PD-R-K-
	Dermatophilus congolensis	654875586	-E---------PT---L-P---KG	P	F---A--V-N--A---V-SSE--TK-
	Glaciibacter superstes	551264617	-LS--------IE-KNL-PEL-KG	P	L---S--VDN--E---V-SDS-----
	Kytococcus sedentarius	506260619	-E---------PT--EM--DM-TG	P	----A--VDN--D---VI-AD---K-
	Leifsonia xyli	545648127	-----------IE-KNL-PEL-KG	S	L---S--V-NE-E---V-SDQ-----
	Micrococcus luteus	488945246	-DS--------PK--DL--DM-KG	P	F---A--T-H--T---V-HPD---K-
	Pseudoclavibacter faecalis	518447966	-----------IE-KNL--EQ-KG	S	L---S--V-N--E---V-SDA-----

Supplementary figure S2

Partial sequence alignment of DNA Topoisomerase I showing a 1 amino acid (aa) deletion that is largely specific for the order *Bifidobacteriales*. Besides *Bifidobacteriales*, a similar deletion is also present in species from the genus *Microbacterium*. Although not shown, this CSI is present in all *Bifidobacterium* species and subspecies.

Group	Species	Accession	79		118
Bifidobacteriales	Bifidobacterium asteroides	408500301	SPAGRPDDIMKNRDWDGITM	T	GRQDALDYLKALDTYYTTF
	Bifidobacterium choerinum	639201949	--S------VRY-T--KFEL	E	---NT----R---K--DS-
	Bifidobacterium animalis	549471548	--S------VRY-T--KFEL	E	---NT----R---K--DS-
	Bifidobacterium gallicum	493338661	--Q-----VLRF-T-PKFEE	-	--K------R---A--D-W
	Bifidobacterium pseudolongum	651884062	--S------VRF-T-PKFEL	D	---NT-E--R---A--DS-
	Bifidobacterium subtile	639064042	--D----A-LR--T-K-VDL	-	--HSTT------CDF-DQL
	Bifidobacterium minimum	551241134	--D----A-VRQ-T-K-VDL	-	--RPTA------ID--DV-
	Bifidobacterium dentium	489934779	--D--A-A-TRF-T-PKFEL	E	--VNT--------A--SE-
	Bifidobacterium thermacidophilum	657871539	--D----A-VRQ-T-K-VDL	-	-HRPTA------ID--DV-
	Bifidobacterium moukalabense	575769010	--D--A-A-TRF-T-PKFEV	E	--GNT--------A--SE-
	Bifidobacterium tsurumiense	651882947	--D--A-SVIRH-T-KEFDL	E	---STE------IE--DS-
	Bifidobacterium longum	494117538	T-D--A-A-VR--T-KSFDA	E	--VGTEE-----IE--DA-
	Bifidobacterium magnum	551239855	TAL------TRF-T-PKFAL	E	--AST----R---E--DVL
	Bifidobacterium bifidum	310286550	T-D--A-A-TR--T-KPFDV	D	--AGTEK--E--IE--DA-
	Bifidobacterium actinocoloniiforme	705421456	------I---T-K-LDL	A	---E-------M-A--SE-
	Bifidobacterium aesculapii	943596171	T-D--A-A-TR--T-KPFDL	E	--AGTEA-----IE--DA-
	Bifidobacterium biavatii	705397851	T-D--A-A-VR--T-KPFDV	E	--AGTTA--E--IE--DA-
	Bifidobacterium bohemicum	705457508	-AQ-T-ES-IE--T-SRF-L	M	---NTQ------ID--AD-
	Bifidobacterium bombi	763214919	-GA-F-LSR-N-KPFEL	K	----TQ------VD--AE-
	Bifidobacterium breve	915495461	T-D--A-A-VR--T-KSFDA	K	--VGTEE-----IE--DA-
	Bifidobacterium callitrichos	759444664	T-D--A-A-TR--T-KAFDV	E	--AGTEE-----IE--DA-
	Bifidobacterium crudilactis	736119052	--D----A-LR--S-K--DL	-	--HSTESF----VE--DS-
	Bifidobacterium cuniculi	705449719	--M------IRF-T--KFEL	E	---NT-E--R---A--DE-
	Bifidobacterium gallinarum	705427528	A-D-SA-A-TR--T-KPFDL	E	--HSTEE--Q--IE--
	Bifidobacterium mongoliense	705438507	-AE-------R--T-K-MDL	-	--HST-------CDF-DS-
	Bifidobacterium psychraerophilum	705401202	--D----A-IR--S-K--DL	-	--HSTESF----VE--DS-
	Bifidobacterium pullorum	705444789	A-D-SA-T-TR--T-KPFDL	E	--HSTEE--Q--IE--
	Bifidobacterium reuteri	763216751	T-D--A-A-TR--T-KPFDV	E	--AGTEA-----IE--DA-
	Bifidobacterium saeculare	705432585	A-D-SA-A-TR--T-KPFDL	E	--HSTEE--Q--IE--
	Bifidobacterium saguini	727804438	T-D----A-VR--T-KPFDV	D	--AGTEK--E--IE--DA-
	Bifidobacterium stellenboschense	736509561	T-D--A-A-TR--T-KPFDV	E	--AGTEE-----IE--DA-
	Bifidobacterium scardovii	672998641	T-D--A-A-TR--T-KQFDV	E	---STEK--E--IE--DA-
	Bifidobacterium stercoris	673003107	--S------VRF-T--KFDL	D	E--NT----R--
	Gardnerella vaginalis	523591919	--S--A---VAS-T-KRFSL	S	--KSTKE--Q--IE--NDM
	Alloscardovia omnicolens	545369903	G-D----AVVR----AEFDL	-	-TNSTEA-I---IA--DE-
	Parascardovia denticolens	493331447	WSD--A-G-IAH-T--QFSL	-	-SR-----V---AD--AS-
	Alloscardovia criceti	516876866	--D----A-VR----SDFNT	S	-T-STEA-I---ID--DE-
	Scardovia inopinata	493335545	WSD--A-G-VA--T-ETFNA	K	-SL-TA---Q--I---VS-
	Scardovia wiggsiae	494250758	WSD--A-A-VG--S-ERFDV	E	-S--TSV-----I---EG-
Other Bacteria 1/500	Pseudonocardia dioxanivorans	331696219	--V-T-GALVR--T--DYSI		--D--IAH-G---LV--GV
	Dermatophilus congolensis	654875376	------G--V-----EDYSL		--DTT-AH-A---KV-EGV
	Haliea salexigens	654481354	I-V-T-AALV-R----DY-L		Q-AL-NEH-A---IV-NGV
	Xylanimonas cellulosilytica	269956580	P-V-T-GALV-A-T--DYSL		--AS-IEH-G---EV-EGV
	Micrococcus luteus	612092753	----T-GALVAA-A--DY-L		--AST-EH-A---LV-DGV
	Isoptericola variabilis	334337032	V-V-T-GALV-A-T-EDYHL		--AT-IEH-G---EV-QGV
	Paraoerskovia marina	656321396	A-Q-T-GA-VA--T--DYSL		--ASTIAH-G----V-QGV
	Kineococcus radiotolerans	152965127	--Y-T-GALVAD-S--DYSI		--AG-IEH-G---LV-SGV
	Gordonia kroppenstedtii	516948479	--V-T-GALVAQ-T--DYDL		R-AN-I-HMG---VV-AGV
	Kocuria palustris	493245981	--D--ASSLVQG-T--DYSV		--AG--EH-G---LV-SGV
	Brachybacterium faecium	257067566	--V-T-GRLVAD-A-SDY-L		-KA-SQEH-T---AV-DGI
	Smaragdicoccus niigatensis	516906604	--Q-T-GA-I-D-T-NDYSL		--DTV-AH-G---VV-RGV
	Nocardioidaceae bacterium	495639301	A-R-T-GALVEE-E-NDYSI		--ATTQEH-A---VT-RGV
	Nocardioides alkalitolerans	655024829	--R-TVGAVAAD-T--DYSV		--DT--AH-G---LV-NGV
	Dietzia alimentaria	498226832	--V-TMGANADH-S--DYSL		--DT--AH-G---KV-DGM
	Cellulomonas flavigena	296129750	A-V-T-GALVAA-T--DYSI		--ASTSEH-G---EV-VGV
	Sanguibacter keddieii	269795168	A-R-T-GV-VSE-S--DYSL		--ATTIAH-G---EV-QGV
	Cellulosimicrobium cellulans	640255144	--Q-T-GALVAA-S-EDYSL		--ASTIAH-G---EV-VGV
	Idiomarina loihiensis	56459186	V---T-QS-VDR-S-KDYSI		--GLVTEH-V---KV-NGV
	Arthrobacter gangotriensis	494480256	--N-L-GALVAA-A--DYEI		--ASTSEH-A---LV--GI
	Jonesia quinghaiensis	656029517	--N-L-G-LTEK-T-NDYPI		--ATTSEH-A---VV-SGV
	Aeromicrobium marinum	494138266	P-L-T-GS-VAG-S--DYSI		A-GTTNEH-G--EMV--QL

Supplementary figure S3

Partial sequence alignment of DNA polymerase sliding clamp subunit showing a 1 aa insert that is specific for *Bifidobacteriales*. Some of the *Bifidobacteriales*-specific CSIs are also present in a few strains of *Chlamydia trachomatis* due to spurious reason. Although not shown, this CSI is present in all *Bifidobacterium* species and subspecies.

Group	Species	Accession	371	Insert	423
Bifidobacteriales	Bifidobacterium asteroides	504834401	TYWKGLLSQDMDPNPTYEEAGRFGRQIAQ	KG	VGERLINLQKRNKVAIMVGNDS
	Bifidobacterium dentium	489935879	--------H-LE-----R---V---E--K	PE	-----VH-K---------S-E-
	Bifidobacterium moukalabense	575770314	--------H-LE-----R---V---E--R	PE	-----VH-K---R-----S-E-
	Bifidobacterium adolescentis	500063617	--------H-LE-----R---V---E--K	PE	-----VH-K-H-------S-E-
	Bifidobacterium catenulatum	489930807	--------H-LE-----R---V---E--K	PE	-----VH-K-H-------S-E-
	Bifidobacterium pseudocatenula	490331452	--------H-LE-----C---V---E--K	PE	-----VH-K-H-------S-E-
	Bifidobacterium longum	658453854	-------GH--E---------V---EV-D	PT	--G--AH-A-C-------S-E-
	B. animalis subsp. lactis	490329894	---R----H-FES-----------E-GD	PR	I-DT-SH-S---A---LAS-E-
	Bifidobacterium tsurumiense	651883196	---R----H-LE-----R---V---E---	DP	LASQ--HMR-H-A-----S-E-
	Bifidobacterium actinocoloniiforme	705420105	--------H-LE---------V---E--D	PQ	---S-RH-R---R-----S-EA
	Bifidobacterium biavatii	705400604	-------GH-FE---------V---E--R	PE	TAS--LH-K---------S-EA
	Bifidobacterium coryneforme	797148885	--------H-LE---------G----V--	--	------H---------------
	Bifidobacterium gallinarum	705425717	--------H-LE-----R---V--HE--D	PK	-----VH-K---------S-E-
	Bifidobacterium indicum	655534950	--------H-LE---------G----V--	--	------H---------------
	Bifidobacterium kashiwanohense	705410402	--------H-LE-----R---V---E--K	PE	-----VH-K-H-------S-E-
	Bifidobacterium merycicum	705457467	--------H-LE-----R---V--HEV-S	PE	-----VH-S---------S-E-
	Bifidobacterium psychraerophilum	705400290	S-------H-LES--------V---E--D	PK	--P-ILH-R-H-P-----S-E-
	Bifidobacterium pullorum	705442743	--------H-LE-----R---V--NE--D	PK	-----VH-K---------S-E-
	Bifidobacterium reuteri	763223911	--------H--E---------V---EV-R	PE	-----VH-G---------S-EA
	Bifidobacterium saguini	727804266	--------H--ES--------V---EV-D	IA	--G--VH-A---------S-EA
	Bifidobacterium stercoris	673002199	--------H-LE-----R---V---E--K	PE	-----VH-K-H-------S-E-
	Alloscardovia criceti	516877235	-----V--H-FAK--------I---EM-A	DS	I-G--VHAT-N-R-----S-EA
	Alloscardovia omnicolens	545370778	-----V--H-FAK--------V---EM-N	PA	R-G--VHAH-N-R--M--S-EA
	Scardovia inopinata	493336726	----------FES--V-----EC--FLQT	E	A-KH-MH-K-N-Q-----S-EA
Other Bacteria 0/500	Sanguibacter keddieii	502631810	--------H-LET--------V---EA-A		A-DA-LH-R-S-R-----S-EA
	Actinomyces dentalis	651278382	----------YS-----R--AD---E--E		HADA-TG-T---RI-V-IS-EA
	Paenibacillus daejeonensis	522107964	--------H-FQ---V----KTI--DF-R		LSDQ--D--VA----V--S-E-
	Robinsoniella sp. KNHs210	653043221	--------H-FKE-A--Q---II-NELKR		I-TK-V--K----T--L-S-E-
	Cohnella laeviribosi	517836971	--------H-FE---V----KTI-KDFDR		LSPH--D-R-Q-R--V--S-EA
	Lactobacillus rossiae	515832273	--------H-FK---V-N--KKI-AELQS		I-SNFV-ST-Q-RI--V-S---
	Lachnospiraceae bacterium	511051464	-----I--H-FQE-D--R--CAI--EFSE		I-SH-M--K-K-D--VL-S-EA
	Bacillus hemicellulosilyticus	569811116	--------H-LKS-------KTV-ADFKK		LSSK-V--S-K-D--MLIS-EA
	Blautia producta	517425084	---R----H-FAE-K--K--VVI-SEMKR		L-S---H-K-Q-------S-EA
	Zymophilus raffinosivorans	518384756	--------H-FR---V-N--KQI-KELQA		Y-SHFL-TS-K----FI-S-N-
	Saccharibacillus kuerlensis	517807931	--------H-LQ---V-N--KTI-ADF-R		LN-S-VG-R-T-RT-VF-S-EA
	Eubacterium ramulus	545614440	---R----H-FKE-HV-C--KTI--DF-A		LSPA-C--K-E----MV-S--A
	Thermobacillus composti	505066340	--------H-FQ--RV-R--QTI--DFER		LSD--AD-KIT-R--ML-S-E-
	Pantoea agglomerans	489944905	--------H-LE---V-N--KNI-QTL-R		LSPQ-TG-K-H-R--LL-SQ--
	Hymenobacter norwichensis	652485498	--------H-LQ--------KTI--DFQR		LSPS-L--K-T-----LFS-QA
	Flectobacillus major	652620309	--------H-FE-----L--QTI--DFEK		LSPK----K-Q----FLFS-EA
	Clostridiales bacterium	646323132	--------H-FEE-AV-R--C-I-G-FRE		LSPS-LH-K-E-------S-EA
	Leuconostoc mesenteroides	504090596	--------H-FE---V----QKV-HDFER		LSDK-V--NHAAD--FV-D-E-
	Enterococcus casseliflavus	514878257	--------H-LQ---V-N--KII-SELKK		YQKE-SG--QK-Q--FV-S-E-
	Amphibacillus jilinensis	516040784	--------H-FK--YIF---ETI-SAFKR		LSPV-TG-K-Q-E--FLIS-EA
	Streptococcus plurextorum	654501811	S--R----H-FAE-K--Q--VTV-NELKR		LSPK-V--K-D-R--LL-S-E-
	Erwinia billingiae	502965951	--------H-LL---V-D--CDI-KTLQR		LTPE-SG-S-S-R--ML-S-RG
	Roseburia hominis	503846332	S----V--H-FSE-A--R--SEI-AEWKR		I-TH-M--K-E-R---LLD-L-
	Spirosoma linguale	502691727	--------H-FQ----FD--KTI-KDFER		LSPQ-V--K-T-Q--VLFS-EA
	Flavobacterium subsaxonicum	652613178	--------H-FQ-----N--MTI-ADFKK		LSPK-AS-KVT--A--LFS-EA
	Segetibacter koreensis	517445264	-----V--H-LQ--R--K-VSQTAKELKE		I-TH-V--K-T-----LFSQ--
	Klebsiella variicola	547401139	--------H-FSR---WQ--TSI-ADF-R		LSPQ-AE-KAE-D--LLIS-EA
	Mitsuokella multacida	492422227	-----V--H-LK-GRI-R-VQEV--EM-R		LSTH-QG-R-K-R--ML-S-EA
	Pectobacterium wasabiae	506210876	--------H-FSR---WQ--TTI-ADF-R		LSSS-DS-RADHD--LL-S-EA
	Raoultella ornithinolytica	505398209	--------H-FSR-A-WQ--TTI-ADF-R		LSPQ-AE-KAE-D--LL-S-EA
	Escherichia coli	651709629	--------H-FSR-A-WQ--TTI-ADF-R		LSPQ-AE-KAE-D--LL-S-EA
	Oxalobacteraceae bacterium	522195371	-----V--H-LE--RA-A-IS-T--ELRK		I-P--V--KLH-D---LWSR--
	Kushneria aurantia	518793317	---R---T-SYQ--RI----STI-ADLQR		L-P--V----D-R---Y-STQA
	Catonella morbi	556511032	-----I--H-LK-GRI-D-ISQI-NE-KT		LTKEKLCIK-N--I-LL-DTL-
	Bacteroides oleiciplenus	496421300	-----I-GH-LE--RI-R-FAATAKELKN		I-SK-V--K-K-D---LYSH--

Supplementary figure S4

Partial sequence alignment of beta-galactosidase showing a 2 aa insert that is specific for the order *Bifidobacteriales*. The homologs of this protein are not found in all *Bifidobacteriales*.

			242		284
Bifidobacteriales	Bifidobacterium tsurumiense	651881972	EGGLNKARWSCSDTAQYGDYTN		TVIGEDCRERMQYHLKRIQDG
	Bifidobacterium thermophilum	470202116	---------------------S		---N-ETKK--E-Q-------
	Bifidobacterium thermacidophilum	657871573	---------------------S		---N-ETKK--E-Q-------
	Bifidobacterium subtile	639062141	-------------------FIS		Q-VDDSTKK--E-Q-G-----
	Bifidobacterium pseudolongum	651888403	------D-------------V-		---D-HA--------Q-----
	Bifidobacterium pseudocatenulatum	656094562	---------------------S		---T-ETKK----Q-------
	Bifidobacterium moukalabense	575769097	----------------------		---N----K------G-----
	Bifidobacterium minimum	651887929	-------------------F-S		---D-HT------Q-------
	Bifidobacterium magnum	651390306	------D-------------V-		-C-D-HV--------Q-----
	Bifidobacterium longum	23465119	---------------------S		---T-ETKK----Q-------
	Bifidobacterium gallicum	493338575	------D-------------V-		---D-HT--------Q-----
	Bifidobacterium dentium	283455064	----------------------		---N----K------G-----
	Bifidobacterium choerinum	639203071	------D-------------V-		---D-HV--------------
	Bifidobacterium catenulatum	489930352	---------------------S		---T-ETKK----Q-------
	Bifidobacterium breve	489924628	----------------------		---N----K--E---Q-----
	Bifidobacterium boum	651391263	---------------------S		---N-ETKK--E-Q-------
	Bifidobacterium bifidum	489908094	----------------------		-C-D-HV--------------
	Bifidobacterium asteroides	408501846	-------------------F-S		--VD-STKD--RHQ-Q-----
	Bifidobacterium animalis subsp. lactis	219682634	------D-------------VS		---D-HT--------Q-----
	Bifidobacterium angulatum	489922072	----------------------		---N----K--E---G-----
	Bifidobacterium adolescentis	489903926	----------------------		---N----K------S-----
	Bifidobacterium actinocoloniiforme	672945001	---------------H---F-S		--VD-STKD--R-Q-------
	Bifidobacterium aesculapii	943596274	---------------------S		---T-ETKK----Q-------
	Bifidobacterium biavatii	705393617	----------------------		---N----K--E---Q-----
	Bifidobacterium bombi	763214927	-------------------F-S		--VD-STKD--RHQ-Q-----
	Bifidobacterium callitrichos	759444755	----------------------		---N----K--E---G-----
	Bifidobacterium coryneforme	705404066	-------------------F-S		--VD-STKD--R-Q-------
	Bifidobacterium crudilactis	736119091	--------------------IS		K-VDDSTKD--R-Q-------
	Bifidobacterium cuniculi	705443372	------D-------------V-		---D-HA--------------
	Bifidobacterium gallinarum	705425897	-------------------F-S		--VD-STKD--R-Q-------
	Bifidobacterium kashiwanohense	860226101	----------------------		-C-D-HV--------------
	Bifidobacterium merycicum	736117865	---M--D---------------		-C-D-HV--------------
	Bifidobacterium mongoliense	705438423	-------------------F-S		---D-HT------Q-G-----
	Bifidobacterium psychraerophilum	705401876	--------------------IS		K-VDDSTKD--R-Q-G-----
	Bifidobacterium pullorum	705444883	-------------------F-S		--VD-STKD--R-Q-------
	Bifidobacterium reuteri	763217000	--------------------V-		---N----K--E---Q-----
	Bifidobacterium ruminantium	705405503	----------------------		---N----K------S-----
	Bifidobacterium saeculare	705429946	-------------------F-S		--VD-STKD--R-Q-------
	Bifidobacterium saguini	727804349	----------------------		---N----K--E---Q-----
	Bifidobacterium scardovii	736519773	---------------------S		---T-ETKK----Q-------
	Bifidobacterium stellenboschense	736513612	---------------------S		---T-ETKK----Q-------
	Bifidobacterium stercoris	655535922	----------------------		---N----K------S-----
	Bifidobacterium indicum	316915364	-------------------F-S		--VD-STKD--R-Q-------
	Alloscardovia omnicolens	545370684	----------------------		--VD-S---------------
	Alloscardovia criceti	516877201	----------------------		---D-S--K--E---G-----
Other Bacteria 0/500	Yaniella halotolerans	551251297	----A-Q---I----EF---VS	GP	R--DARVK-N--EV--DV---
	Varibaculum cambriense	566218143	---IT-Q--------E----VS	GP	R--TDQT-QA-RDI-SG-R--
	Trueperella pyogenes	644255599	----T-Q---I----E----VS	GP	R--TAQVK-N--AV-AD----
	Saxeibacter lacteus	656114342	---IA-Q---V----E----VS	GP	R--DDHVAQN--AV-AD-K--
	Renibacterium salmoninarum	163839792	---IA-Q---V----E----VS	GP	L--DAHVKDN-KAV--D----
	Pseudoclavibacter soli	654790796	----A-Q---I----E----VS	GP	R--DPHVK-N-KAV-AD--S-
	Paraoerskovia marina	656321726	---IA-Q---V----E----VS	GP	R--DPSVK-N-KAV-AD--N-
	Nocardioides halotolerans	655241146	---IA-Q---V----E----VS	GP	R--D-HVK-N-KQV-AE-K--
	Mobilicoccus pelagius	497127473	---IA-Q---I----E----VS	GP	R--DSHVK-N--GV-AD--N-
	Microbacterium yannicii	518009702	---IA-Q---V----E----VS	GP	R--DPHVK-N--AV-AD--S-
	Kribbella flavida	284032770	---IA-Q---V----E----VS	GP	RI-DASVKA---DV-ND-K--
	Kineococcus radiotolerans	152965305	---IA-Q---V----EW---VS	GP	R--DASVK---KDV--D----
	Janibacter hoylei	495203854	---IA-Q---I----E----VS	GP	R-VDAKVK-S-KGI-SD----
	Intrasporangium calvum	317125483	---IA-Q---V----E----VS	GP	R--DPSVK-N-KAV-GD--N-
	Geodermatophilus obscurus	284992453	---IA-Q---V----E-----S	GP	K--DQHVKDA-KDV-A--K--
	Desulfotomaculum ruminis	334338962	----SRM-Y-V-N--E----MI	GP	RI-T-ET--E-R-V-QE----
	Cellulosimicrobium cellulans	640261197	---IA-Q---V----E----VS	GP	R--DAHVK-N--GV-AD--S-
	Brachybacterium paraconglomera	498238128	---IA-Q---I----E----VS	GP	R-VTDQTKAD-KAI-AD--S-
	Actinobaculum urinale	551246680	----T-Q--------E----VS	GP	R--TPVVK-N-KEV-AD--S-
	Acaricomes phytoseiuli	648482927	---IA-Q---V----E----VS	GP	R--DPRVK-N-KEV--D----

Supplementary figure S5

Partial sequence alignment of ketol-acid reductoisomerase showing a 2 aa deletion that is specific for the order *Bifidobacteriales.* Although not shown, this CSI is present in all *Bifidobacterium* species and subspecies.

Group	Organism	ID	74		119
Bifidobacteriales	Bifidobacterium adolescentis	489903803	VEVRVAPWGAIKILDGPASD	PH	NLTPPDVIELDPDVWMRLATGITS
	Bifidobacterium angulatum	489921854	----------V---------	--	------------E--L-------T
	Bifidobacterium animalis	490328901	-------YAVV---E-----	--	------------Q--L-V-S---T
	Bifidobacterium asteroides	504834007	----------V---------	--	---------------L---A---D
	Bifidobacterium bifidum	489907952	----------V---------	--	------------E--L---A---T
	Bifidobacterium boum	651391221	--------------------	--	---------------L---C---T
	Bifidobacterium breve	489924574	----------V------E--	--	------------E--L---C---T
	Bifidobacterium catenulatum	489930260	--------------------	--	----------E--------A----
	Bifidobacterium choerinum	639201936	----------V---------	--	----------E-A--L-V-S----
	Bifidobacterium dentium	489934761	--------------------	--	----------E-------------
	Bifidobacterium gallicum	493338634	----------V---------	--	----------E-S--L-----V-T
	Bifidobacterium longum	499381003	----------V------E--	--	------------E--L---C---T
	Bifidobacterium magnum	551239790	-------FS-V---------	--	------------P--L-------T
	Bifidobacterium minimum	551241122	----------V------R--	--	------------TT-L---S-M-R
	Bifidobacterium moukalabense	575769019	--------------------	--	----------E-------------
	Bifidobacterium pseudocatenulatum	490333408	--------------------	--	----------E-------------
	Bifidobacterium pseudolongum	551237476	--------------------	--	------------S--L-V-S-V-T
	Bifidobacterium ruminantium	651887024	--------------------	--	------------------------
	Bifidobacterium subtile	639064069	--------A-V---------	--	---------------L---C----
	Bifidobacterium thermacidophilum	657871634	--------------------	--	----------K----L---C---T
	Bifidobacterium thermophilum	505262480	M-----------------	--	----------K----L---C---T
	Bifidobacterium tsurumiense	651882956	--------------E-----	--	---------------------V-T
	Bifidobacterium actinocoloniiforme	705421438	--------------------	--	------T-----E--L---A-V--
	Bifidobacterium aesculapii	943596197	----------V---------	--	-------V--E-S--L-V---V-T
	Bifidobacterium biavatii	705393945	--------------------	--	----------E----L---A---T
	Bifidobacterium bohemicum	705457574	--------S-V---------	--	----------E----L-M-C-L-T
	Bifidobacterium callitrichos	759444642	--------------------	--	------------Q--L---A----
	Bifidobacterium coryneforme	705404399	--------------------	--	------------E--L--------
	Bifidobacterium crudilactis	736119060	--------A-V------N--	--	------T---E-E--L---C--S-
	Bifidobacterium cuniculi	705449645	--L----F--V---E-----	--	----------E----L---C---
	Bifidobacterium gallinarum	672964309	--------------------	--	------------E--L-----V-T
	Bifidobacterium kashiwanohense	746131120	--------------------	--	----------E-------------
	Bifidobacterium merycicum	705458454	----------V---------	--	---------------L---S----
	Bifidobacterium mongoliense	705438492	--------------------	--	----------E-E------C-L-T
	Bifidobacterium psychraerophilum	705402680	----------V------S--	--	------T--------L-V-C----
	Bifidobacterium pullorum	672987852	--------------------	--	------------E--L-----V-T
	Bifidobacterium reuteri	763217057	----------V---------	--	----------E-E--L---C---T
	Bifidobacterium saeculare	672990431	--------------------	--	------------E--L-----V-T
	Bifidobacterium saguini	727804649	----------V------E--	--	----------E----L---C---T
	Bifidobacterium scardovii	705448025	--------------------	--	------------E--L---A---T
	Bifidobacterium stellenboschense	736513463	----------V---------	--	---------------L-V---V-T
	Bifidobacterium indicum	655534655	--------------------	--	------------E--L--------
	Bifidobacterium stercoris	673001142	--------------------	--	------------------------
	Gardnerella vaginalis	523608354	----------V---E--Q--	--	---------------L--SC---K
	Alloscardovia omnicolens	545369891	-------FA-V---E--Q--	--	------------A--L--VC----
	Scardovia inopinata	493335353	--L----YA-V------K--	--	----------E----L---C----
	Scardovia wiggsiae	494251526	--L-I--YA-V---E--K--	--	----------E-A--L-M-C-F-T
	Parascardovia denticolens	493332911	--L----F------E--R--	--	---------V--P--L---C---T
	Alloscardovia criceti	648490047	-------FA-V-V-E--K--	--	------A---E-A--L---C---M
Other Bacteria 0/500	Actinokineospora inagensis	651291338	-----P-FA-VQCVE--RHT		RG---N-V-MA-RT-LL----RLT
	Beutenbergia cavernae	506364285	-----P-A--TQAIA--RHT		RG---N-V-MR-HT-LA----R-R
	Clavibacter michiganensis	500977104	-----P-F--VQCIE--GHT		RG---N---T--AT-IA----G-T
	Demetria terragena	516904378	-----P-F--VQVIE--RHT		RG--TN-V-ME-AT-LL----SMA
	Gordonia araii	494532914	-----P-FA-VQCIE--RHT		RG---N-V-T--RT-L-----AV-
	Haloglycomyces albus	658649207	-----P-F--VQVVE--RHR		RG---N---TT-E-FLD-V--KIT
	Intrasporangium chromatireducens	586970901	-----P-F--VQCIE--VHR		RG---N---T-AAT-L--V--DLE
	Jonesia denitrificans	506252828	-----P-A--VQCV---RHT		RG---NTV-MN-HT-LE----TVT
	Kineosphaera limosa	493643179	-----P-F--VQCIE--RHT		RG---N-V-T-AQT-L---V-DLT
	Mobiluncus curtisii	490106767	-----P-F--VQ--P-TTHR		RG---A---MS-E--LA--V-GLT
	Paraoerskovia marina	656319947	----IP-A--VQCVE--RHT		RG---N-V-T--QT-LE--S-TLT
	Rhodococcus rhodnii	498803633	-----P-FV-VQCIE--RHT		RG---N-V-T--RT-LE------T
	Saccharopolyspora rectivirgula	658527033	-----P-FA-VQCVE--RHT		RG---N-V-T--RT-LE----R-G
	Salinispora arenicola	501131590	-----P-Y--VQCVP--RHT		RG---N-V-M--ET-L-V---AL-
	Verrucosispora maris	503496212	-----P-Y--VQCVP--RHT		RG---NTV-M--QT-LA----RLE

Supplementary Figure S6

Partial sequence alignment of serine-pyruvate aminotransferase showing a 2 aa insert that is specific for the order *Bifidobacteriales*. This insert is also present in two strains of *C. trachomatis* (SwabB1 and HiIMS) due to spurious causes. Although not shown, this CSI is present in all *Bifidobacterium* species and subspecies.

Group	Species	ID	42		82
Bifidobacteriales	Bifidobacterium angulatum	489922190	DGAKVTLAAADLAKVSVKAEVV	D	DEAKGPKINIQKYKNKTG
	Bifidobacterium animalis	490388967	--E--II-------I-------	N	-D------R---F-----
	Bifidobacterium asteroides	504835356	--S---M--K----I---G---	-	------------F-----
	Bifidobacterium breve	489926438	----------------------	N	------------------
	Bifidobacterium choerinum	639202650	--S---V-------IT------	-	--------R---F-----
	Bifidobacterium dentium	489934207	---N-I-G-K----L------I	N	----------M-------
	Bifidobacterium gallicum	493338993	---N----SG----I-------	-	--------R---F-----
	Bifidobacterium longum	501574122	--D-------------------	N	--------D---------
	Bifidobacterium magnum	651390229	--S--VM--K---GMT------	N	----------M-F-----
	Bifidobacterium minimum	551240413	--ST---S-K---DR-------	-	--V-----S---F-----
	Bifidobacterium moukalabense	575769309	---N-I-G-K----L-------	N	----------M-------
	Bifidobacterium pseudolongum	551237918	--S---I-------I---G---	-	--------R---F-----
	Bifidobacterium subtile	639062375	--SQ---G-K----L-------	-	------------F-----
	Bifidobacterium tsurumiense	651881911	--S----G-K-----------L	N	----------M-------
	Bifidobacterium actinocoloniiforme	705419627	---------K------A-G---	-	--L-----R---F-----
	Bifidobacterium aesculapii	943596960	---N--I-------I-------	K	-D----------------
	Bifidobacterium biavatii	705394987	-----V-G-K---AI---G---	-	----------M-------
	Bifidobacterium bohemicum	705457737	------IG-K------------	-	AQ----------F-----
	Bifidobacterium bombi	763214567	------M--K----I-------	N	------------F-----
	Bifidobacterium callitrichos	759443755	---N------------------	N	------------------
	Bifidobacterium crudilactis	736119698	------V--K----I-------	-	--------R---F-----
	Bifidobacterium cuniculi	705446982	--S-----------IT--G---	N	-D------R---F-----
	Bifidobacterium mongoliense	705435730	-------G-K---QL-------	-	----------M-F-----
	Bifidobacterium psychraerophilum	705397291	------V--K----I-------	-	--------R---F-----
	Bifidobacterium pullorum	705441638	-------G-K---AI------I	-	-S--------M-------
	Bifidobacterium reuteri	763217447	---N--------------G---	-	----------M-------
	Bifidobacterium saguini	727804163	--T----G-K---A--------	-	------------------
	Bifidobacterium stellenboschense	736511071	---N----------I-------	K	-D------S---------
	Bifidobacterium coryneforme	671341489	------M--K----I---G-I-	-	------------F-----
	Bifidobacterium indicum	655535767	------M--K----I---G-I-	-	------------F-----
	Bifidobacterium gallinarum	672964960	-------G-K----I-------	N	-S--------M-------
	Bifidobacterium merycicum	672974738	----------------------	-	------------------
	Bifidobacterium pseudocatenulatum	225158596	--S--V-G-K---N--------	N	--------S-M-------
	Bifidobacterium saeculare	672993535	-------G-K----I-------	N	-S--------M-------
	Bifidobacterium scardovii	672995165	-----V-G-K---A----G---	-	----------M-------
	Bifidobacterium stercoris	673001356	--D---VN-K---NI-------	N	--------S-M-------
	Gardnerella vaginalis	490213545	-------S-K----IT-----L	N	--------S-M-------
	Alloscardovia criceti	516877767	--S------K----I-------	-	--------S---F-----
	Scardovia inopinata	493336660	--SQ--V--K----I-------	N	-----K--T---F-----
	Scardovia wiggsiae	494251738	--S---V--K------------	-	-----K--H---F-----
	Alloscardovia omnicolens	545371833	--S--V---K----MT---Q--	-	--------S---------
	Parascardovia denticolens	493332524	--S---M--K--T-I-------	-	-----E--S-M-F-----
Other Bacteria 0/500	Acaricomes phytoseiuli	648483130	--E-I-SD-K-----K----IL		ENLR----V---F-----
	Acidothermus cellulolyticus	500038874	--PS--TG-TA--DIT-TG---		GH------R-L-------
	Amycolatopsis rifamycinica	637500161	--GE--TD-DA------TGK--		EQT-----R-H-F-----
	Brachybacterium muris	516435082	--DS--S-KS--D--K-T--IL		E-LR----R-L-------
	Brevibacterium casei	496826347	--DA--TD-DA-S-I--S----		--LR----V---------
	Corynebacterium vitaeruminis	643727449	---N--SKSEE------AG-I-		EHV-----K-M-------
	Glaciibacter superstes	551265025	--ETI-SDSKS----K-T---L		NDLR----V---F-----
	Intrasporangium calvum	503257558	---T--SD--K-----------		KP------T-I-F-----
	Leucobacter salsicius	516404970	--D---TD--E----T-T---L		NDLR----I--R------
	Microbacterium gubbeenense	656181773	--DN--T--DA-S-IT-T---M		NDLR----V---------
	Nocardia asteroides	517875574	---DL-TD-DK---I--A----		-QT-----R-H-F-----
	Propionibacterium acidipropion	504882957	--ES--SQKDA------T---L		G-------H-L-F-----
	Renibacterium salmoninarum	501203587	--E-I-SD-K-----K----IL		EDLR----V---F-----
	Rhodococcus triatomae	494796605	--SEL-TD-DK----E-TG---		EHT-----R-H-F-----
	Saccharopolyspora erythraea	497633566	--SD--AD-DA------TGKL-		EQT-----R-H-F-----
	Tetrasphaera australiensis	586945804	--TT--SD-SK------Q----		GP------T-M-F-----
	Tomitella biformata	640107323	---DL-ID-DA---A--TG-I-		EHT-----R-H-F-----
	Tsukamurella paurometabola	502891158	---NL-TD--A-E-I--TG---		-HV-----R-H-F-----
	Varibaculum cambriense	654141410	--DQ--SD----E-IK-T---L		EQT-----R-L-F-----

Supplementary figure S7

Partial sequence alignment of 50S ribosomal protein L21 showing a 1 aa insert that is specific for *Bifidobacteriales*. This insert is also present in a strain of *C. trachomatis* (SwabB1) due to spurious causes. Although not shown, this CSI is present in all *Bifidobacterium* species and subspecies.

Group	Species	Accession	34		70
Bifidobacteriales	Bifidobacterium adolescentis	547078960	GTNLLEIDEFVHKRIVDR	K	GASSCYVDYAPDFGTGPF
	Bifidobacterium angulatum	489922239	-------------K----	-	--E---------------
	Bifidobacterium animalis	490329309	---------I-----A--	-	--Q---------------
	Bifidobacterium asteroides	504835125	-V----L-DY-KD--NS-	-	--T---------------
	Bifidobacterium boum	651390450	-----D--DY-RR--ES-	-	--E---------------
	Bifidobacterium breve	489926814	-------------K----	-	--E---------------
	Bifidobacterium catenulatum	489932392	------------------	-	--E---------------
	Bifidobacterium choerinum	639201886	------------Q-----	-	--E---------------
	Bifidobacterium dentium	489933715	------------------	-	--T---------------
	Bifidobacterium gallicum	493338117	-------------K----	-	--T---------------
	Bifidobacterium longum	548571624	-------------K----	-	--E---------------
	Bifidobacterium magnum	551239186	---------Y--Q-----	-	--E---------------
	Bifidobacterium minimum	551241185	--------D--KQ-VLS-	Q	--E---------------
	Bifidobacterium moukalabense	575769570	------------------	-	--T---------------
	Bifidobacterium pseudolongum	651883750	------------Q-----	P	--E---------------
	Bifidobacterium ruminantium	651886637	------------------	-	--T---------------
	Bifidobacterium subtile	639063005	-----------A---A--	-	--E---------------
	Bifidobacterium tsurumiense	651882088	--------D--RRQ-ES-	-	--E---------------
	Bifidobacterium pseudocatenulatum	490330300	------------------	-	--E---------------
	Bifidobacterium kashiwanohense	705411640	------------------	-	--E---------------
	Bifidobacterium angulatum	489922239	-------------K----	-	--E---------------
	Bifidobacterium reuteri	763217247	-------------K----	-	--E---------------
	Bifidobacterium saguini	727804305	-------------K----	-	--E---------------
	Bifidobacterium bifidum	489911477	---------Y---K----	-	--E---------------
	Bifidobacterium stellenboschense	736510670	-----D-------K----	-	--E---------------
	Bifidobacterium thermacidophilum	673004418	-----D--DY-RR--ES-	-	--E---------------
	Bifidobacterium aesculapii	943597108	-----D-------K----	-	--E---------------
	Bifidobacterium callitrichos	759442902	-----D-------K----	-	--E---------------
	Bifidobacterium biavatii	705398322	-------------K----	-	--E----N----------
	Bifidobacterium cuniculi	705447356	------------R-----	P	--E---------------
	Bifidobacterium merycicum	705459386	-------------K---C	-	--E---------------
	Bifidobacterium mongoliense	705436154	-----------A-K-A--	-	--E---------------
	Bifidobacterium pullorum	705444982	--------D--RR--ES-	-	--E---------------
	Bifidobacterium saeculare	705429307	--------D--RR--ES-	-	--E---------------
	Bifidobacterium bombi	763214366	--------DI-RR--ES-	-	--E---------------
	Bifidobacterium thermophilum	763423833	-----D--DY-RR--ES-	-	--E---------------
	Bifidobacterium crudilactis	736120131	--------D--KQ--EG-	S	--E---------------
	Bifidobacterium bohemicum	705454295	--------DL-RR--EG-	-	--E----N----------
	Bifidobacterium actinocoloniiforme	705422073	-V----L-DY-KRQ-ES-	R	--E---------------
	Bifidobacterium coryneforme	671341303	-V---DL-NH-KE--QS-	-	--T---------------
	Bifidobacterium gallinarum	672965138	--------D--RR--ES-	-	--E---------------
	Bifidobacterium indicum	655535581	-V---DL-NH-KE--QS-	-	--T---------------
	Bifidobacterium scardovii	672996352	-------------K----	-	--E----N----------
	Bifidobacterium stercoris	673001575	------------------	-	------------------
	Gardnerella vaginalis	490209044	--------D--RQ--MS-	-	------------------
	Alloscardovia criceti	648490121	--------N--KHA-EN-	A	--A---------------
	Parascardovia denticolens	493331814	-----D--NY-KEQ-EA-	P	D-E---------------
	Scardovia inopinata	493335903	--------SY-KQ--DQ-	P	D-Q---------------
	Scardovia wiggsiae	494250091	-----D--NY-KGQ-ES-	P	D-Q---------------
	Alloscardovia omnicolens	545372845	--------N--KNA-EN-	A	--T---------------
Other Bacteria Indel (-) 1/500	Actinomadura rifamycini	651270521	-----D--RWTREM--EA		--E------E-S--R---
	Cellulosimicrobium cellulans	640262017	-----QV-QWAREM-DAA		--T--------S--R---
	Corynebacterium variabile	663155508	--S-----RWAREM-E-A		--Q--------S--R---
	Demetria terragena	516899248	-V----L-ALA-RM-K-A		--E---I--H-S--AS--
	Georgenia sp. SUBG003	660684664	-V----L-ALA-DM-RE-		--E---I--H-S--AS--
	Humibacter albus	551279556	--------GWTSQL-T-A		--E--------S--R---
	Jonesia denitrificans	506252237	--------QWAKDM-LGA		--T--------S--S---
	Kineococcus radiotolerans	500725602	--------RWARDM-EEA		--E--------S--R---
	Leifsonia aquatica	545650616	-V----L-RIA-DL-RR-		--E---I--H-S--AS--
	Marmoricola aequoreus	663705811	-V----L--LA-RM-KE-		--E---I--H-S--AM--
	Mobilicoccus pelagius	497131454	--------RWA-EH-RR-		--E---I--H-S--AS--
	Nakamurella multipartita	506226539	-----D--AWAREL-ARA		--V--------S--R---
	Oerskovia turbata	663110044	-V----L-ALA-RM-K--		--E---I--H-S--AS--
	Promicromonospora sukumoe	518859776	-V----L--VA--M-R-Q		--E---I--H-S--AM--
	Rhodococcus triatomae	494793982	-----D--DWTRAM-AEA		--R--------S--R---
	Salinispora pacifica	517022234	-V--M-LEHHARR--AQ-		--E---W----S--R---
	Streptomyces sulphureus	518375733	--S--D--RWTKAM-DEA		--Q--------S--R-A-
	Verrucosispora maris	503497798	-V--MDLEHHARR--AE-		--E---W----S--R---
	Xylanimonas cellulosilytica	502643580	-V----L-ALA-QM-K--		--E---I--H-S--AM--

Supplementary figure S8

Partial sequence alignment of methionine aminopeptidase showing a 1 aa insertion that is specific for the order *Bifidobacteriales*. Additionally, a similar CSI is also present in *Streptococcus pyogenes.* Although not shown, this CSI is present in all *Bifidobacterium* species and subspecies.

			534		574
Bifidobacteriales	Bifidobacterium adolescentis	500062906	VEPEPSVETVERGAAMMR	E	EFEPDTIIAVGGGSPMDASKIM
	Bifidobacterium angulatum	489923950	-------D---K------	-	------------------A---
	Bifidobacterium animalis	504295556	------------------	D	------------------A---
	Bifidobacterium asteroides	504835258	------------------	D	--R---------------A---
	Bifidobacterium bifidum	489911075	--------------E---	-	------------------A---
	Bifidobacterium boum	651390572	--------------S---	D	--K-----------A---A-V-
	Bifidobacterium breve	489926541	-----------K------	-	----------------------
	Bifidobacterium catenulatum	489932691	--------------T---	-	----------------------
	Bifidobacterium choerinum	658450016	------------------	D	------------------A---
	Bifidobacterium dentium	489934063	------------------	-	----------------------
	Bifidobacterium gallicum	493338322	------------------	-	--Q---------------A---
	Bifidobacterium longum	499381171	-----------K--E---	-	----------------------
	Bifidobacterium magnum	551239672	------------------	-	--Q-----------------V-
	Bifidobacterium minimum	551240484	--------------S---	D	------------------A---
	Bifidobacterium moukalabense	575769383	------------------	-	----------------------
	Bifidobacterium pseudolongum	551237770	------------------	D	------------------A---
	Bifidobacterium ruminantium	651886861	------------------	D	----------------------
	Bifidobacterium subtile	639065399	------------------	-	------------------A---
	Bifidobacterium thermacidophilum	657871241	--------------S---	D	--K-----------A---A-V-
	Bifidobacterium thermophilum	505263873	--------------S---	D	--K-----------A---A-V-
	Bifidobacterium tsurumiense	651881811	--------------E---	-	--------------S-------
	Bifidobacterium actinocoloniiforme	672943648	I------------SE---	D	D-K---------------A---
	Bifidobacterium aesculapii	943597237	--------------E---	-	----------------------
	Bifidobacterium biavatii	705395748	-----------K--E---	-	------------------A---
	Bifidobacterium bohemicum	705455066	I------D------E---	D	--Q------I--------A---
	Bifidobacterium bombi	763215317	------ID----------	-	--Q------I--------A-V-
	Bifidobacterium callitrichos	759442482	-----------K--E---	-	------------------A---
	Bifidobacterium coryneforme	797149619	--------------E---	D	--G---------------A---
	Bifidobacterium crudilactis	736119844	--------------K---	-	------------------A---
	Bifidobacterium cuniculi	672961546	------------------	D	------------------A---
	Bifidobacterium gallinarum	705423145	-----------K--G---	-	------------------A---
	Bifidobacterium indicum	655535699	--------------E---	D	--G---------------A---
	Bifidobacterium kashiwanohense	746131667	--------------T---	-	----------------------
	Bifidobacterium merycicum	705458246	-------D---K------	-	------------------A---
	Bifidobacterium mongoliense	705436338	--A---------------	D	------------------A---
	Bifidobacterium pseudocatenulatum	490330600	--------------T---	-	----------------------
	Bifidobacterium psychraerophilum	917315092	--------------K---	-	------------------A---
	Bifidobacterium pullorum	759448201	-----------K--G---	-	------------------A---
	Bifidobacterium reuteri	763216649	-----------K--E---	-	----------------------
	Bifidobacterium saeculare	705428986	-----------K--E---	-	------------------A---
	Bifidobacterium saguini	727803242	-----------K--E---	-	----------------------
	Bifidobacterium scardovii	705445946	--------------E---	-	------------------A---
	Bifidobacterium stellenboschense	736513637	-----------K--E---	-	----------------------
	Bifidobacterium stercoris	673001434	------------------	-	----------------------
	Gardnerella vaginalis	502678122	------------------	D	--Q-------------------
	Alloscardovia omnicolens	545376701	--S---I-----------	D	--Q------I--------A---
	Alloscardovia criceti	516877876	--S---ID----------	D	--Q------I--------A---
	Parascardovia denticolens	493331521	-------Q------T---	-	D-Q---------------A---
	Scardovia inopinata	535478490	--S----Q----------	-	D-H------I--------A---
	Scardovia wiggsiae	494250672	--S----Q------Q---	-	D-H------I--------A---
Other Bacteria 0/500	Cellulomonas fimi	503537904	-------R--QN---Q--		H-R------L--------A-V-
	Clostridium botulinum	490578909	---D------MN---I--		--Q--L--G------I--A-A-
	Haemophilus parahaemolyticus	491983099	---N--LQ--Q--TEL--		S-Q------L--------A---
	Kitasatospora cheerisanensis	641395180	---N--IA---K--EL--		G-R---VV-L--------A-V-
	Lachnoclostridium phytofermentans	657702722	---D------MK------		-----W-VSM-----I--A-A-
	Mannheimia varigena	575447067	---N--LQ--Q--TEL--		S-Q------L--------A---
	Nakamurella multipartita	506228634	-------Q--YK--DL--		D-K------L--------A-V-
	Paenibacillus curdlanolyticus	492906493	---D---------TTL-E		N-Q--C---L--------A-A-
	Ruminococcus bromii	505336361	---D------MK---V--		-----W-V-M-----I--A-A-
	Streptococcus suis	636968892	---D-DIT------EV--		A-Q------L--------A-V-
	Xylanimonas cellulosilytica	502642751	-----------K--QL--		DHR------I----------V-
	Abiotrophia defectiva	557370011	---D-DIT-------L--		--Q------L----V---A-V-
	Actinoplanes missouriensis	504253740	------ID--Q----L--		S-R------L--------A-V-
	Bacillus cereus	487973550	---D---T--QN--N--E		D-Q--W---------I--A-V-
	Bibersteinia trehalosi	505244922	---N--I---RK-TEL--		S-Q------L--------A-V-

Supplementary figure S9

Partial sequence alignment of bifunctional acetaldehyde-CoA/alcohol dehydrogenase showing a 1 aa insert that is specific for *Bifidobacteriales*. Although not shown, this CSI is present in all *Bifidobacterium* species and subspecies.

Group	Species	Accession	809		845
Bifidobacteriales	Bifidobacterium adolescentis	500062906	TPEEGVENLAKAVEDYRD	N	KLGMNKSFQECGVDEDYY
	Bifidobacterium angulatum	489923950	--A---------------	-	----------------FF
	Bifidobacterium animalis	504295556	--A---------------	-	--------KD-------F
	Bifidobacterium asteroides	504835258	-T-----L--R---S---	E	R---DA---AA----GF-
	Bifidobacterium bifidum	489911075	--A---------------	-	---------D---E---F
	Bifidobacterium boum	651390572	----------R-I-----	-	----DA--KA------T-
	Bifidobacterium breve	489926541	--A-A-------------	-	---------D-------F
	Bifidobacterium catenulatum	489932691	------------------	-	-----------------F
	Bifidobacterium choerinum	658450016	--A---------------	-	---------D-------F
	Bifidobacterium dentium	489934063	--A---------------	-	-----------------F
	Bifidobacterium gallicum	493338322	----TT------------	-	-----------------F
	Bifidobacterium longum	658455123	--ADA-------------	-	---------D--------
	Bifidobacterium magnum	551239672	----A-----S-------	-	--------KD-------F
	Bifidobacterium minimum	551240484	----------L---E---	E	-----S---QL------F
	Bifidobacterium moukalabense	575769383	--A---------------	-	-----------------F
	Bifidobacterium pseudolongum	651883828	--A-------A-------	-	---------D-------F
	Bifidobacterium ruminantium	651886861	------------------	-	------------------
	Bifidobacterium subtile	639065399	-DA-------R-------	-	--D-D----A-------F
	Bifidobacterium thermacidophilum	657871241	----------R-IQ----	-	----DA--KA------T-
	Bifidobacterium thermophilum	505263873	----------R-I-----	-	----DA--KA------T-
	Bifidobacterium tsurumiense	651881811	------------------	-	Q------------E-E-F
	Bifidobacterium actinocoloniiforme	917298775	S-QDA--KY-Q-L-----	-	----DS---SA----E-F
	Bifidobacterium aesculapii	943597237	--A-A-------------	-	-----------------F
	Bifidobacterium biavatii	705395748	--A-A-----R-------	-	---------D-------F
	Bifidobacterium bohemicum	705455066	--AQ------L-------	-	----SS--K------E-F
	Bifidobacterium bombi DSM 19703	671259300	----------C-I-----	D	----DSC--Q-----SR-
	Bifidobacterium callitrichos	759442482	--A-A-----R-------	-	-----------------F
	Bifidobacterium coryneforme	705403006	-------L--R-------	Q	----DS---AA------F
	Bifidobacterium crudilactis	736119844	---TA--A--T-------	-	R---DPT--AA----E-F
	Bifidobacterium cuniculi	917314358	--A---------------	-	---------D-------F
	Bifidobacterium gallinarum	705423145	--A-------R-------	-	--Q------D--------
	Bifidobacterium indicum	705389824	-------L--R-------	Q	----DS---AA------F
	Bifidobacterium kashiwanohense	746131667	------------------	-	-----------------F
	Bifidobacterium merycicum	705458246	--A----S----------	-	---------D------FF
	Bifidobacterium mongoliense	705436338	-A------F-S-L-----	-	R-S-DSC-KD-----ET-
	Bifidobacterium psychraerophilum	672986913	S--HA--A--G-------	-	R---DPT--AA-------
	Bifidobacterium pseudocatenulatum	490330600	------------------	-	-----------------F
	Bifidobacterium pullorum	672986472	--A-------R-------	-	--Q------D--------
	Bifidobacterium reuteri	763216649	--A-A-------------	-	--------KD-------F
	Bifidobacterium saeculare	736514775	--A-------R-------	-	--Q------D--------
	Bifidobacterium saguini	727803242	--A-A-------------	-	--------KD-------F
	Bifidobacterium scardovii	705445946	--A-A-------------	-	--------KD-------F
	Bifidobacterium stellenboschense	736513637	--A-A-------------	-	-----------------F
	Bifidobacterium stercoris	673001434	------------------	-	--------K---------
	Gardnerella vaginalis	523592810	--Q-------R---N---	-	L---DA--KA-----E--
	Alloscardovia criceti	516877876	-------K--Q-------	-	----DA--KAA----KHF
	Parascardovia denticolens	493331521	-------S--RGI-----	-	R---D----AA------F
	Scardovia inopinata	493335599	----------A-L-----	-	----DHD-KA------FF
	Scardovia wiggsiae	494250672	----------A-L-----	-	R---DHD-RS------FF
	Alloscardovia omnicolens	551236765	-AD-A-----N--A----	-	----DA---AA----KHF
Other Bacteria 0/500	Actinoplanes missouriensis	504253740	---Q---SY-R---RL--		AV-IPP---GA--N-QEF
	Bibersteinia trehalosi	505244922	--A----SY----H-LSV		RC-VKM-LK-Q-I--KAF
	Cardiobacterium valvarum	494043569	------QS--Q--Y-LGE		RV-IKMN-RDQ-IN-QD-
	Cellulosimicrobium cellulans	640254024	-------SY-T---EL-A		-V-IEPT-AAQ----RAF
	Endozoicomonas elysicola	522070237	------KS--N--R-LMK		R-D-PATI--I----SLF
	Firmicutes bacterium	547319918	------K---D--DRLAK		EI-IKMN-KDA-IE-SDF
	Jonesia denitrificans	506252294	-------SY-R-I-EL-E		AV-IPA---LQ--N-EAF
	Kitasatospora cheerisanensis	641395180	---Q---S--L---EL--		RV-IPR--K-A----AAF
	Lachnoclostridium phytoferment	501157379	NDN-KF---L--IDELKE		-V-IK--IK-Y----K-F
	Nakamurella multipartita	506228634	--A----SY-T---QL-A		MV-IPA---AQ----QVF
	Paraoerskovia marina	656321234	--D-A--SY-R---VL-S		-V-IEA--R-Q----QAF
	Sanguibacter keddieii	502629337	--A----SY-----AL--		-V-IAP---AQ--A-EDF
	Thermobacillus composti	505069352	-D--KL-S-IRKI-ELKE		---IK-TIRDY-I--EEF

Supplementary figure S10

Partial sequence alignment of bifunctional acetaldehyde-CoA/alcohol dehydrogenase showing a 1 aa insert that is specific for *Bifidobacteriales*. Although not shown, this CSI is present in all *Bifidobacterium* species and subspecies.

			367		416
Bifidobacteriales	Bifidobacterium adolescentis	500063439	WATWSDAGFGDDGRTMVTKTSFRLLN	TL	TLEHLGPGPEPNITIFWDPKLP
	Bifidobacterium angulatum	489922771	---------------L----------	--	----------------------
	Bifidobacterium animalis	490328174	--------------------------	--	----------------------
	Bifidobacterium asteroides	504834597	--------------PL---------A	--	----------------------
	Bifidobacterium bifidum	489910259	--------------P-----------	--	----------------------
	Bifidobacterium boum	651390745	--------------C-----------	--	----------------------
	Bifidobacterium breve	643831812	--------------P-----------	--	----------------------
	Bifidobacterium catenulatum	489931185	---------------L----------	--	----------------------
	Bifidobacterium choerinum	639202096	------G-------------------	--	----------------------
	Bifidobacterium dentium	489936405	---------------L----------	--	----------------------
	Bifidobacterium gallicum	493336923	---------------L----------	--	----------------------
	Bifidobacterium longum	499380647	--------------S-----------	--	----------------------
	Bifidobacterium magnum	551238730	---------------L----------	--	----------------------
	Bifidobacterium minimum	551240601	--------------S-----------	--	----------------------
	Bifidobacterium moukalabense	575770636	---------------L----------	--	----------------------
	Bifidobacterium pseudocatenulatum	490333116	---------------L---------D	--	----------------------
	Bifidobacterium pseudolongum	551238343	---------------L----------	--	----------------------
	Bifidobacterium ruminantium	651886428	--------------------------	--	----------------------
	Bifidobacterium subtile	639061174	--------------S-----------	--	----------------------
	Bifidobacterium thermacidophilum	657871873	--------------C-----------	--	----------------------
	Bifidobacterium thermophilum	505263192	--------------C-----------	--	----------------------
	Bifidobacterium tsurumiense	651882867	--------------P-----------	--	----------------------
	Bifidobacterium actinocoloniiforme	705420441	---------S----PL---------A	--	-MD-------------------
	Bifidobacterium aesculapii	943596116	--------------PL----------	--	----------------------
	Bifidobacterium biavatii	705392598	---------A----P-----------	--	----------------------
	Bifidobacterium bohemicum	705455413	--------------H----------A	--	--D---------------A---
	Bifidobacterium bombi	763213834	--------M-----H----------A	--	--D--------------N----
	Bifidobacterium callitrichos	759441833	--------------PL----------	--	----------------------
	Bifidobacterium coryneforme	705401690	--------------PL---------A	--	----------------------
	Bifidobacterium crudilactis	736121027	--------------S-----------	--	----------------------
	Bifidobacterium cuniculi	705444361	------G-------------------	--	----------------------
	Bifidobacterium gallinarum	705424582	--------------------------	--	----------------------
	Bifidobacterium indicum	705387730	--------------PL---------A	--	----------------------
	Bifidobacterium kashiwanohense	672971030	---------------L----------	--	----------------------
	Bifidobacterium merycicum	705458946	---------------L----------	--	----------------------
	Bifidobacterium mongoliense	705435107	--------------P-----------	--	----------------------
	Bifidobacterium psychraerophilum	705398436	--------------SQ----------	--	----------------------
	Bifidobacterium pullorum	705441244	--------------------------	--	----------------------
	Bifidobacterium reuteri	763215963	--------------PL----------	--	----------------------
	Bifidobacterium saeculare	705430487	--------------------------	--	----------------------
	Bifidobacterium saguini	727804855	--------------PL----------	--	----------------------
	Bifidobacterium stellenboschense	736514066	--------------PL----------	--	----------------------
	Gardnerella vaginalis	490207929	---------SE----F----------	--	----------------------
	Alloscardovia criceti	516878191	---------------L----------	--	----------------------
	Parascardovia denticolens	493332136	---------------L----------	--	----------------------
	Alloscardovia omnicolens	545375885	---------------L----------	--	----------------------
	Scardovia inopinata	493336179	---------------L----------	--	--D-------------------
	Scardovia wiggsiae	494248984	---------A-----L----------	--	--D-------------------
Other Bacteria 0/500	Brachybacterium muris	516433643	--------MTE---SQ---------Q		--RN---A------V-------
	Actinobaculum massiliense	494059462	------G---N----L---------Q		--RN---A-----------A--
	Corynebacterium ulceribovis	516654755	---------SE---SL---------Q		--RN---A-----------Q--
	Edwardsiella hoshinae	639182960	---E-L--M-L----L--R----F--		--YTM--S----M--L-SE---
	Enterococcus gallinarum	537117769	---ETL--M-V----L---N---M--		--YTM--S----L--L-SE---
	Gilliamella apicola	637111848	---E-I--M-V----L-------F--		--YTM--S----M--L-SE---
	Klebsiella pneumoniae	571166675	---E-IG-M-V----L---N---F--		--YTM--S-------L-SE---
	Lonsdalea quercina	652343250	---E-L--M-L----L-------F--		--YTM--S----M--L-SE---
	Mahella australiensis	503547833	-V-E-IG-M-L----L-------M-H		---N---A----L-VL-SNR--
	Pectobacterium wasabiae	506210558	---E-L--M-L----L-S-----F--		--YTM--S----M--L-SE---
	Salmonella enterica	603590757	---E-IG-M-V----L---N---F--		--YTM--S------VL-SE---
	Streptomyces davawensis	505473106	-V---M--L-E---PL--R-T--A--		--YN---A----L-V--S-R--
	Trueperella pyogenes	640530397	---------------L---------Q		--MN---A---------SEN--
	Varibaculum cambriense	551245057	----------E----L---------Q		--IN---A---------S-R--
	Xenorhabdus cabanillasii	575850125	---E-I--M-L----L---N---F--		--YTM--S----M--L-SE---
	Yersinia aldovae	490837924	---E-L--M-V----L-------F--		--YTM--S----M--L-SE---

Supplementary figure S11

Partial sequence alignment of formate acyltransferase showing a 2 aa insert that is specific for *Bifidobacteriales*. Although not shown, this CSI is present in all *Bifidobacterium* species and subspecies.

Group	Species	ID	131		163
Bifidobacteriales	Bifidobacterium adolescentis	547078870	LWTLHDDSRPA	D	DRCFETLLDAWRNTPTAALLG
	Bifidobacterium angulatum	489923470	--L--------	N	AE-LSE--E-Q----G-SI--
	Bifidobacterium animalis	504510729	--L--------	-	PI-L-Q---T-T-------I-
	Bifidobacterium asteroides	504834871	--L----C--L	-	NH-LDK-V-T-HSN---S---
	Bifidobacterium bifidum	489909274	V-L--------	-	-T-L-S--ET---D---SV--
	Bifidobacterium boum	651390694	--M--------	N	NQ-L-SM-ET---A---SI--
	Bifidobacterium breve	489925556	V-M-------S	-	NW-L-R--E----ASG-GI--
	Bifidobacterium catenulatum	489931929	-----------	-	ES-L-A-----------S---
	Bifidobacterium choerinum	639202856	--L---E----	-	E--L-R-----A-S-------
	Bifidobacterium dentium	489937282	--LM-------	-	EY-L-S-----------S---
	Bifidobacterium gallicum	493338878	--L--------	-	-Q-L-H--E--AY-----I--
	Bifidobacterium longum	494111121	--L-------S	-	-S-L-R--E------G-SV--
	Bifidobacterium magnum	551238791	--L--------	-	QW-L-R--E--S------I--
	Bifidobacterium moukalabense	575769885	--L--------	-	EH-L-S-----------S---
	Bifidobacterium pseudolongum	551238169	--L--------	-	---L-A---------------
	Bifidobacterium ruminantium	651886152	-----------	-	G--L---------N---S---
	Bifidobacterium subtile	639061547	V-M--------	-	AH-L-N--ET---A-------
	Bifidobacterium thermacidophilum	657871756	--M--------	-	NI-L-S-IET-H-A---SI--
	Bifidobacterium thermophilum	505263283	--M--------	-	NI-L-S-IET-H-A---SI--
	Bifidobacterium tsurumiense	651882356	V-M--------	-	EH-L----E---SA---S---
	Bifidobacterium actinocoloniiforme	705418830	--L-------L	-	ED-L---VGT--D--A-S---
	Bifidobacterium aesculapii	943596555	--L--------	-	GH-L-R-VE------G-S---
	Bifidobacterium biavatii DSM 23969	672954562	----------	-	ED-LAH-------NSS-S---
	Bifidobacterium bohemicum	705455458	--L--------	-	SH-L-S-IE-------IS-I-
	Bifidobacterium bombi	763213810	--L--------	-	R--LAS-VE-K------S---
	Bifidobacterium callitrichos	759443483	--L--------	-	---L-Q-VET-----G-T-I-
	Bifidobacterium coryneforme	705404251	--L-------M	-	PN-L---IES-HRS--VSI--
	Bifidobacterium crudilactis	917260623	--L-------R	-	PE-LD--VQ----A-G-SI--
	Bifidobacterium cuniculi	705443903	--L--------	-	-H-L------K----------
	Bifidobacterium gallinarum	705424769	--------K--	-	A -L-A--E-R------S---
	Bifidobacterium indicum	705388196	--L-------M	-	PN-L---IES-HRS--VSI--
	Bifidobacterium kashiwanohense	746134611	-----------	-	ES-L-S-----------S---
	Bifidobacterium merycicum	705457214	--L--------	N	AN-L-V--E-Q----G-S---
	Bifidobacterium minimum	672977925	--L-------M	G	R--L-C--EVKS---S-DVV-
	Bifidobacterium mongoliense	705435013	--M----A---	-	E----H--ET---A-S-TV--
	Bifidobacterium pseudocatenulatum	490332136	-----------	-	ES-L-V-----K-----S---
	Bifidobacterium psychraerophilum	705398549	--L-------R	-	QE-LD--VE----A-G-S---
	Bifidobacterium pullorum	759446833	-----------	-	A -L----E-R------S---
	Bifidobacterium reuteri	763216070	--L-------S	-	AV-L-H--E------G-SI--
	Bifidobacterium saeculare	705430611	-----------	-	A -L-A--E-R------S---
	Bifidobacterium saguini	727803042	--L-------S	-	AY-L-H--E----A-G-SV--
	Bifidobacterium scardovii	673000036	--L--------	-	-E-LSR-------N---S---
	Bifidobacterium stellenboschense	673005968	--L--------	-	E--L-H-VE----S-G-S---
	Gardnerella vaginalis	532641999	--L-----K-C	-	SK-L-A-RET-------CV--
	Alloscardovia criceti	648490185	M-L-------L	-	NTYVDM-NEVRH-NAS-TII-
	Parascardovia denticolens	493331929	-LL-------V	G	TSYL-G--ETKEKN-S-TVI-
	Scardovia inopinata	493335956	-AVF------L	-	E-YL-L--E-R--N-H-TVI-
	Scardovia wiggsiae	494249640	-LL-------V	G	EQYI-S-TE-Q--N-S-SVI-
	Alloscardovia omnicolens	545376380	M-L-------L	-	-SYVD-MNEVR--NAL-TVI-
Other Bacteria 0/500	Catenulispora acidiphila	506276382	--L-----E--		PDALLR--EVGEHH-E-GVI-
	Cellulomonas flavigena	502882594	--L-----A--		PDALGR-VRTVS-A-SV-VA-
	Lechevalieria aerocolonigenes	663696288	--L-----A-E		PD-LAA--T-AEVS-S--V--
	Lentzea albidocapillata	663699666	--L-----A-E		PD-LAA--T-TEVS-S--V--
	Actinokineospora enzanensis	648624432	I-L-----A-D		PD-LS---L-AEVS-A-GV--
	Actinomyces viscosus	537932628	F-L-----A--		ED-L-R--T-AT-ARSVGIV-
	Amycolatopsis vancoresmycina	489180663	--L----CA-E		RD-L-Q--TVATKE-S-TV--
	Saccharopolyspora spinosa	498001442	--L-----A-E		PE-L-H--R-AEEDSI-----
	Saccharothrix espanaensis	504917851	--L----CA-E		PD-LSL--T-AEVS-S--V--
	Streptomyces cattleya	503908792	--L----CE--		PDALAE--RVADT--S--VV-
	Varibaculum cambriense	551244006	--L-----A-D		PA-LGE-VQ-FE-ST-LGIA-

Supplementary figure S12

Partial sequence alignment of ATP synthase F0 subunit A showing a 1 aa insert that is specific for *Bifidobacteriales*. This indel in also present in some strains of *C. trachomatis* (SwabB1 and HiIMS) due to spurious causes. Although not shown, this CSI is present in all *Bifidobacterium* species and subspecies.

Group	Species	Accession	197		237
			QGRIQTSAAGVIVFPEADDDN	DE	IDIDPKDLKIDIFMSSGP
Bifidobacteriales	Bifidobacterium angulatum	489924412	QGRIQTSAAGVIVFPEADDDN	DE	IDIDPKDLKIDIFMSSGP
	Bifidobacterium animalis	490329291	------------------E-D	--	-E----------------
	Bifidobacterium asteroides	504835141	------------------E-D	--	-E-------V--------
	Bifidobacterium boum	651390466	--------------------D	--	-E----------------
	Bifidobacterium breve	489926776	------------------E-D	--	-E----------------
	Bifidobacterium catenulatum	489932361	------------------E-D	--	-E--Q-------------
	Bifidobacterium choerinum	639201900	------------------E-D	--	-E---------T------
	Bifidobacterium dentium	489933778	------------------E-D	--	------------------
	Bifidobacterium gallicum	493338131	------------------E-D	--	-E----------------
	Bifidobacterium longum	547239588	------------------E-D	--	-E----------------
	Bifidobacterium magnum	551238615	--------------------D	--	-E----------------
	Bifidobacterium minimum	651887962	--------------------D	--	-VV---------------
	Bifidobacterium pseudocatenulatum	656094056	------------------E-D	--	-E--Q-------------
	Bifidobacterium pseudolongum	651883764	-----------L------E-D	--	-E----------------
	Bifidobacterium ruminantium	651886620	------------------E-D	--	-E----------------
	Bifidobacterium subtile	639063029	--------------------D	--	-EV---------------
	Bifidobacterium thermacidophilum	657871347	--------------------D	--	-E----------------
	Bifidobacterium thermophilum	505263720	--------------------D	--	-E----------------
	Bifidobacterium tsurumiense	651882066	------------------E-D	--	-E----------------
	Bifidobacterium merycicum	705459520	---------------------	--	------------------
	Bifidobacterium pullorum	705444675	---------------------	--	-E----------------
	Bifidobacterium moukalabense	736877333	--------------------D	--	------------------
	Bifidobacterium mongoliense	705436112	------------------E-D	--	------------------
	Bifidobacterium bifidum	829064813	------------------E-D	--	-E----------------
	Bifidobacterium coryneforme	799123816	------------------E-D	--	-Q----------------
	Bifidobacterium stellenboschense	736510697	------------------E-D	--	-E----------------
	Bifidobacterium adolescentis	705407090	------------------E-D	--	-E----------------
	Bifidobacterium biavatii	705398355	------------------E-D	--	-E----------------
	Bifidobacterium indicum	705388750	------------------E-D	--	-Q----------------
	Bifidobacterium saguini	727805057	------------------E-D	--	-E----------------
	Bifidobacterium actinocoloniiforme	705419496	--------------------D	--	-E----------------
	Bifidobacterium reuteri	763217233	------------------E-D	--	-E----------------
	Bifidobacterium cuniculi	705448894	------------------E-D	--	-E----------------
	Bifidobacterium aesculapii	943597770	------------------E-D	--	-E----------------
	Bifidobacterium callitrichos	759442860	------------------E-D	--	-E----------------
	Bifidobacterium bohemicum	705454344	------------------E-D	--	-E----------------
	Bifidobacterium bombi	763214043	------------------E-D	--	-E----------------
	Bifidobacterium kashiwanohense	746131929	------------------E-D	--	-E--Q-------------
	Bifidobacterium crudilactis	736120114	-----------------E--D	--	----A-------------
	Bifidobacterium psychraerophilum	705398147	------------------E-D	--	VEV-A-------------
	Bifidobacterium gallinarum	672965152	---------------------	--	-E----------------
	Bifidobacterium saeculare	672993768	---------------------	--	-E----------------
	Bifidobacterium scardovii	672996372	------------------E-D	--	-E----------------
	Bifidobacterium stercoris	673001556	------------------E-D	--	-E----------------
	Gardnerella vaginalis	523594351	------------------E-D	--	-E----------------
	Alloscardovia criceti	516877605	-----------------E---	--	-E--Q-------------
	Parascardovia denticolens	493334845	--------------------D	--	-EV---------------
	Scardovia inopinata	493335871	--------------------D	--	-E----------------
	Scardovia wiggsiae	494250142	--------------------D	--	-T----------------
	Alloscardovia omnicolens	545372742	-----------------E---	--	-E--Q-------------
Other Bacteria 0/500	Actinobaculum urinale	551246096	-------T---L--A-V--PG	-	VE-G-N--R--VYR----
	Brevibacterium mcbrellneri	492548785	----H------L----V-EPE	-	-QL-DN-----VYR----
	Cellulomonas fimi	503537884	----H------L-L---E-EG	-	V----N--R--VYR----
	Isoptericola variabilis	503605345	----H------L----V--PG	-	VE---N--R--V-R----
	Jiangella gansuensis	652480581	----H------L-L---E-EA	-	VE-S-N--R--V-R----
	Kocuria rhizophila	518139155	---VH------L----V-EPD	-	---SQN-----VYR----
	Leifsonia aquatica	545653939	----H------L----V-EPE	-	V--N-N-----V-R----
	Mobiluncus curtisii	490106584	----H------L-M---EETG	D	-E---N--R--VYR----
	Rothia aeria	493944890	----H------L----V-EPE	-	-E-SQN-----VYR----
	Varibaculum cambriense	654140436	----H------L-M--VE-AG	-	-E---NEVRV-V-R----
	Xylanimonas cellulosilytica	502642806	----H------L----V--PG	-	VE---N--R--VYR----
	Zunongwangia profunda	502837588	---VH----T-M-L---EEFD		VE-----VR--Y-C----
	Herbidospora cretacea	663667139	----H------L-Y---EEVD		VQ---N--R--VYR----
	Kribbella catacumbae	521054521	----H------L-L---E-VD		VE--QN--R--V-R----
	Rhodothermus marinus	503832281	----H----S-V-L---EEVD		VE-R-E--R--V-R----

Supplementary figure S13

Partial sequence alignment of peptide chain release factor 1 showing a 2 aa insert that is specific for *Bifidobacteriales.* Although not shown, this CSI is present in all *Bifidobacterium* species and subspecies.

Group	Species	ID	224		265
	Bifidobacterium adolescentis	489905014	GFAREVADQIVFMDGGVVVEQGGPE		IIDHPSEPRFKDFLQHV
	Bifidobacterium angulatum	489922663	-------------------------		---N-T-----------
	Bifidobacterium animalis	490329333	---K----------------R-T-D		-----K-E--Q------
	Bifidobacterium asteroides	504835114	-------E------Q---------D		-----RTE--AE--HT-
	Bifidobacterium boum	651390434	---------V----------H---D		-----------------
	Bifidobacterium breve	489925144	--------------------K----		---N-T-----------
	Bifidobacterium catenulatum	489932428	-------------------------		---N-Q-----------
	Bifidobacterium choerinum	639201873	---K-----V----------S----		---N-K-----------
	Bifidobacterium dentium	489933687	---------V---------------		---N-R-----------
	Bifidobacterium gallicum	493338094	----------------K-------D		-----Q----Q------
	Bifidobacterium longum	494110507	--------------------K---D		-----T-----------
	Bifidobacterium magnum	551239201	---K-----V---------------		-----K-A--Q------
	Bifidobacterium minimum	651887955	---K-----V----E-I---R----		-----G----Q---H--
	Bifidobacterium moukalabense	575769582	--------------------K----		---D-H-----------
	Bifidobacterium pseudocatenulatum	490330265	-------------------------		---N-Q-----------
	Bifidobacterium pseudolongum	651883722	-------------------------		-----R-V--R------
	Bifidobacterium ruminantium	651886650	-------------------------		-----------------
	Bifidobacterium subtile	639062972	------G------N------K----		-L-N-T-Q------N--
	Bifidobacterium thermacidophilum	657871373	---------V----------H----		-----------------
	Bifidobacterium thermophilum	505263683	---------V----------H----		-----------------
	Bifidobacterium tsurumiense	651882097	---K---------------------		-V----S---Q---N--
	Bifidobacterium scardovii	820199604	---------V---------------		-----------------
	Bifidobacterium stellenboschense	736513270	---------V----------S----		-----T-----------
Bifidobacteriales	Bifidobacterium biavatii	672957480	---------V--------------D		-----T-----------
	Bifidobacterium merycicum	705459026	-------------------------		---N-T-----------
	Bifidobacterium kashiwanohense	705411669	-------------------------		---N-Q-----------
	Bifidobacterium reuteri	763217671	---------V----------K----		--E--T-----------
	Bifidobacterium saguini	727802006	---------V----------K----		---N-T-----------
	Bifidobacterium cuniculi	705447393	------------------------D		---Q-K----R------
	Bifidobacterium callitrichos	759442920	---------V----------H-TSD		-----T-----------
	Bifidobacterium bombi	763214066	---K-----V---------------		---N-TS---SE--S--
	Bifidobacterium aesculapii	943597115	---------V----------A----		-----Q-------
	Bifidobacterium bohemicum	672951208	---------V----------R-SSS		-----A----Q---SN-
	Bifidobacterium mongoliense	705436491	--------------------R-DSG		-V-D-K----R---N--
	Bifidobacterium coryneforme	799124869	--------------E---------K		-----ATD--RS--DT-
	Bifidobacterium indicum	917502550	--------------E---------K		-----ATD--RS--DT-
	Bifidobacterium actinocoloniiforme	705422104	-------G-V----------S---D		-----ESR--QE--RT-
	Bifidobacterium crudilactis	917260528	---------V----------S-TSD		-----KTE--SS--KS-
	Bifidobacterium psychraerophilum	672987090	---------V----Q-----H--SD		-----QTE--GA--KS-
	Bifidobacterium stercoris	673001588	-------------------------		-----K-S---------
	Gardnerella vaginalis	657896738	---------V----D-M---C-TAD		---N-QTN------H--
	Alloscardovia criceti	516877572	---K----------------S-TSD		-----QTD--R---TA-
	Parascardovia denticolens	493334817	---------V---A---I--S-D-S		------TS------DS-
	Scardovia inopinata	535477574	---K-----V-------I--S---D		-----Q-Q---G--NS-
	Alloscardovia omnicolens	545372890	---K----------------S-SSD		-----QTD--QE---A-
	Brachybacterium squillarum	498218672	---------V----Q-AII-R-T--	Q	V--N-QSE-LQG--NS-
	Corynebacterium maris	529144781	---------V----------S-P-A	E	VL-N-Q-A-TQQ--SSL
	Facklamia sourekii	654658840	---A--G-RV------YI--N-T-D	Q	-FN--Q-A-TQA--EK-
	Arthrobacter siccitolerans	635354025	----K----V----E-E---A-T-G	D	LF-N-KSE-LQR--SE-
	Bacillus bataviensis	494147136	N--K----RVI------I----TSA	D	-F---K-E-T-R--SK-
	Gulosibacter molinativorax	652542387	--------RV------K---SSA-A	D	LF---K-S-TQ---SK-
	Kandleria vitulina	656033547	------S-RVI-----YI--E-N-Q	E	L-N--K-K-TI---NQ-
Other Bacteria 0/500	Kribbella flavida	502687231	A-------RV-------I----S--	D	V-GN-TQE-TQN--RR-
	Leifsonia aquatica	545656725	--------RV--L-D-----E-T-A	D	VL---R-K-TRE--SR-
	Mesorhizobium opportunistum	503659822	-----IG--L-------I--R-A-R	E	M-AN-KS--TRE--SR-
	Paenibacillus larvae	558473311	---K--G-RVL-----FI--E-T--	E	-FS--KHE-TQS--SK-
	Rhodospirillales bacterium	653047549	---------V-------IA-R-P-S	S	-LRS-KNE-TRE--TR-
	Streptomyces avermitilis	499297346	--------RV------RI----P-A	E	VL---KHE-TR---SK-
	Treponema maltophilum	513873911	--------KVL---N-L-A-K-T--	Q	-FN--QNK-LQQ---R-
	Veillonella dispar	491529839	---K-----V---E---I----D-K	V	VLE--K-E-TRK--SRY
	Xylanimonas cellulosilytica	502640439	--------EV----Q-------P-S	E	VL-A-R-Q-TR---A--

Supplementary figure S14

Partial sequence alignment of arginine ABC transporter ATP-binding protein showing a 1 aa deletion that is specific for *Bifidobacteriales*. This indel is also present in some strains of *C. trachomatis* (SwabB1 and HiIMS) due to spurious causes. Although not shown, this CSI is present in all *Bifidobacterium* species and subspecies.

Group	Species	ID	338		388
Bifidobacteriales	Bifidobacterium adolescentis	489905793	NPDKAALYDRIKAGELPEGFDKAI	DDLE	ATFEVGKNVATRGASGSTLNAIA
	Bifidobacterium angulatum	489922943	-----------------------L	----	D-------I--------V-----
	Bifidobacterium animalis	490328476	--------N---N-----E-----	----	-G--A-SK----K---AVI----
	Bifidobacterium asteroides	504834110	--KR-------RRH-P-KEL-S--	-E--	NELH--DS-PA-Q---RV-----
	Bifidobacterium bifidum	545409181	----------L------------L	----	-------K---------V-----
	Bifidobacterium boum	651391059	--------E----HK------E--	-KF-	-AQ----G----KS--QV-----
	Bifidobacterium breve	489926010	------------------------	----	-------G---------V-----
	Bifidobacterium catenulatum	489931475	---N------L-------------	----	-------G---------V-----
	Bifidobacterium choerinum	639202915	--------E---N----AE-----	----	-G--A-SK----K---AVI----
	Bifidobacterium dentium	489936852	------------------------	----	-------G---------A-----
	Bifidobacterium gallicum	493337659	--------E-----K------A--	-E--	-G-N--DK----K---AAI----
	Bifidobacterium longum	494112011	----------L------------L	----	-------G---------V-----
	Bifidobacterium magnum	551239015	--------E-----K------A--	-E--	-G----AK----K---AAI----
	Bifidobacterium minimum	551240281	----------L---D---------	--V-	SG--S-SK----K---VV-----
	Bifidobacterium moukalabense	575770090	------------------------	----	-------G---------V-----
	Bifidobacterium pseudolongum	551237083	------------------D-----	----	------SK----K---AA---V-
	Bifidobacterium ruminantium	651886384	------------------------	----	-----------------------
	Bifidobacterium subtile	639063929	--------E---------------	----	-S--A-SK----K---AV-----
	Bifidobacterium thermacidophilum	657872133	--------E----HK------E--	-TF-	-AQ----G----KS--QV-----
	Bifidobacterium thermophilum	505263492	--------E----HK------E--	-AF-	-AQ----G----KS--QV-----
	Bifidobacterium tsurumiense	651882154	------------T-D------E--	-KAV	EG-----GI-------TI-----
	Bifidobacterium stellenboschense	736510073	----------L-------------	----	-----------------V-----
	Bifidobacterium saguini	727803652	------------------------	----	-------G---------V-----
	Bifidobacterium aesculapii	943597266	----------L-------------	----	-------S---------V-----
	Bifidobacterium merycicum	705458795	-----V------------------	----	----A------------V-----
	Bifidobacterium callitrichos	759441679	----------L-------------	----	-------G---------V-----
	Bifidobacterium scardovii	736519723	----------L------------L	----	-------K---------V-----
	Bifidobacterium kashiwanohense	746133033	---N------L-------------	----	-------G---------V-----
	Bifidobacterium pseudocatenulatum	490332689	---N------L-------------	----	-------G---------V-----
	Bifidobacterium biavatii	705392774	--------E-L------------L	----	-------G---------V-----
	Bifidobacterium reuteri	763215747	-------------K---D-----	----	-------G---------V-----
	Bifidobacterium cuniculi	705445558	--------E---S-K------Q--	-E--	-S----AK----K---NVI----
	Bifidobacterium gallinarum	705423812	--------E--H--K---D-----	----	-GY---AK----K---AV---L-
	Bifidobacterium pullorum	705442372	--------E--H--K---D-----	----	-GY---AK----K---AV---L-
	Bifidobacterium mongoliense	705436901	----Y---E-LV--K---------	----	SS--A-PK----K---AVI----
	Bifidobacterium bohemicum	705453688	----SR------------------	----	-GWAA-DKA---K---QV---M-
	Bifidobacterium psychraerophilum	705400262	-----------A--K---D-----	-E-V	-G-TA-SK----K---AV-----
	Bifidobacterium crudilactis	736120754	-----------A--K---D-----	-E-V	-G-TA-SK----K---AV-----
	Bifidobacterium actinocoloniiforme	672944293	-----S-----HS-K---D--Q--	-QA-	-A--T-SKL---K---AV-----
	Bifidobacterium bombi	763214234	-----------H--K------E--	-KAI	-A-KA-DK----K---QV-----
	Bifidobacterium coryneforme	671340945	-----------H--K---------	----	-G--P-SK----K---AVI--L-
	Bifidobacterium indicum	655535245	-----------H--K---------	----	-G--P-SK----K---AVI--L-
	Bifidobacterium saeculare	672991800	--------E--H--K---D-----	----	-GY---AK----K---AV---L-
	Bifidobacterium stercoris	673003380	------------------------	----	-----------------------
	Gardnerella vaginalis	523594270	----------L---K---------	---V	-G----SK----K---AV-----
	Alloscardovia criceti	516878306	-----------S------D-----	-E--	-GISA-AS----K---AA-----
	Parascardovia denticolens	493332066	--A----H---HE-R---E-----	-E--	-S----SS----K---AVI--L-
	Scardovia inopinata	493336099	-------H---HE-K---E-----	----	------SS----K---AVI--L-
	Scardovia wiggsiae	494249125	-------H---H--R---E-NRV-	----	----A-SS----K---AVI--L-
	Alloscardovia omnicolens	545375595	-------F--MA-----AD-N---	-E--	-GVTA-AS----K---AA-----
Other Bacteria (0/250)	Actinomadura oligospora	651278921	-----------SRR-----WK--V		PS-AA--DL---A---QV---L-
	Arthrobacter arilaitensis	503114516	-SEE---LA-LQ-------WTEK-		P---P-STM---A---KVI----
	Brevibacterium album	656050723	Q-ER-Q-F--LQS-A--A-LRE-L		PE--A-SKI---A---TV---L-
	Curtobacterium flaccumfaciens	516498820	-A--K--F--VQSK-----LEA-L		PV-PAD-A-S--A---KVI----
	Glaciibacter superstes	551264875	--ERKV-L---LS-D--D-V-A-L		PV--G-TEMS--A---KV---LG
	Humibacter albus	551279235	--ERK--L--LLS------VAE-L		PV--P--D-S--A---KV---L-
	Kocuria palustris	493242105	H--R-E----LRD-T----YAE-F		PQ-PA-ET----A---KVI----
	Leifsonia aquatica	545657351	--ERKQ-L--LLT------VEDVL		PV--P--D-S--A---KV-----
	Microbacterium yannicii	518009084	--ERK--L--LES----QDIAS-L		PS--D--D-S--A---LVI--L-
	Nesterenkonia alba	655014902	---S------LLN----Q-WEEVL		PE-PA-EA----A---KVI--V-
	Rothia mucilaginosa	502667675	--ER-E--K-LV-----ADYKA-F		PV--A-TS----A---TVI----
	Streptosporangium amethystogenes	664378246	--ER--EF---SVRQ--A-WSE-L		PI----SS----K---EV--SL-
	Thermobispora bispora	502897249	--ER-E-F---SRR-----WH-VL		PA--P-SSI---K---EV---L-

Supplementary figure S15

Partial sequence alignment of transketolase protein showing a 4 aa insert that is specific for the order *Bifidobacteriales*. Although not shown, this CSI is present in all *Bifidobacterium* species and subspecies.

			362		405
Bifidobacteriales	Bifidobacterium adolescentis	547084095	RMTVLVEDLLSLARLDEGRGIDI	T	QQVKLTSVVRDAADDLHALD
	Bifidobacterium angulatum	489921716	----------------------M	-	---S---L-A--T-------
	Bifidobacterium animalis subsp. lactis	588478325	--------------------VSM	N	-R-DF---MQ-SV-------
	Bifidobacterium asteroides	504835001	------T---------------T	-	LP-D--GL---GV-------
	Bifidobacterium bifidum	489909116	-----------------------	-	---P--TL-T--TE------
	Bifidobacterium breve	489926961	----------------------V	-	GT---S-L-T--V-------
	Bifidobacterium catenulatum	489933222	-----------------------	-	---------K----------
	Bifidobacterium choerinum	639202400	---------------------S-	-	-R-DIGA-LA-S-S------
	Bifidobacterium dentium	489937475	-----------------------	-	---P----------------
	Bifidobacterium gallicum	493337870	---L-----------------SV	D	-T---C--L--SL-------
	Bifidobacterium longum subsp. longum	665772361	----------------------M	-	GT---S-L-T--V-------
	Bifidobacterium moukalabense	575769694	-----------------------	-	---P----------------
	Bifidobacterium pseudocatenulatum	656093921	-----------------------	-	---------N----------
	Bifidobacterium ruminantium	651886764	-----------------------	-	---------K----------
	Bifidobacterium subtile	639063787	---L------------------V	-	-H-Q--GLLA----------
	Bifidobacterium thermacidophilum	657872533	----------------------V	-	---NI--QLT--C-------
	Bifidobacterium thermophilum	505263550	----------------------V	-	---NI--QLT--C-------
	Bifidobacterium actinocoloniiforme	914249196	---E--Q-------M-----V-T	S	LD-N---L-N--V-------
	Bifidobacterium aesculapii	943595964	----------------------M	-	-T-S--G--G--L-------
	Bifidobacterium biavatii	672957149	----------------------L	-	---P---I-S--T-------
	Bifidobacterium bohemicum	763226201	---L------------------L	-	-D-N-DQLLE--V-------
	Bifidobacterium bombi	671259971	--S-----------------V--	-	-L-R-DR-FE-S--------
	Bifidobacterium boum	917009856	----------------------V	-	---LI--QLN--C-------
	Bifidobacterium callitrichos	917512904	----------------------M	-	-T-S--TL-T--V-------
	Bifidobacterium coryneforme	671341172	---D--S--------------NT	N	LP-DM-GL---SV-------
	Bifidobacterium cuniculi	917314137	---L--------------H---M	-	-HIN-GA-M--C--------
	Bifidobacterium gallinarum	672965304	-----------------------	-	---N--AI-K----------
	Bifidobacterium indicum	655535461	---D--S--------------NT	N	LP-DM-GL---SV-------
	Bifidobacterium kashiwanohense	746132220	-----------------------	-	---------K----------
	Bifidobacterium magnum	917286201	---A----------------VNP	-	--IRMSTILN-SI-------
	Bifidobacterium merycicum	672973604	----------------------M	-	-E-S---L-T--T-------
	Bifidobacterium minimum	672978294	---------------------M	-	-E-S---L-T--T-------
	Bifidobacterium mongoliense	672984942	-----------------------	-	---AVRPLIA----------
	Bifidobacterium psychraerophilum	672987247	--A-------------------V	H	-E---S---N--V-------
	Bifidobacterium pullorum	917516093	-----------------------	S	---H---I------------
	Bifidobacterium reuteri	918251088	----------------------V	-	GS-N-A-L-T--V-------
	Bifidobacterium saeculare	672994143	-----------------------	-	---N--AI-K----------
	Bifidobacterium scardovii	673000277	-----------------------	-	---P---LIS--S-------
	Bifidobacterium stellenboschense	672999828	----------------------M	-	-T-S-----A--L---Y---
	Bifidobacterium stercoris	673001685	-----------------------	-	---------C----------
	Bifidobacterium tsurumiense	763402658	---E-------------------	-	--IR-N-ILS----------
	Gardnerella vaginalis	532643045	---E----------F------NV	S	-T-NV-PILK--TN--R---
	Parascardovia denticolens	493332725	---S---S------M-------L	A	-R-RVDQL-EES-E------
	Scardovia inopinata	493336783	--SS---A--------------L	G	-KMR-DQIIKE--E------
	Scardovia wiggsiae	494251991	--SS---A--------------L	G	-KIGVDQI-KES-E------
	Alloscardovia omnicolens	545373466	--SA------------------L	A	-T-HM-DILS-SVE------
Other Bacteria 0/500	Actinobaculum massiliense	494059184	--SG------Q---M----PLQL		EE-S-SD-AE-SLY--S-RA
	Cellulomonas fimi	503535739	--GT------A------N-PLRH		DP-D--VLAA--VS--R---
	Geodermatophilus obscurus	502715570	--G-------Q-----QQ-PLT-		TP-D-AE-AG--VH-AR--Q
	Isoptericola variabilis	503605625	--GT--N---V--------PMRH		EP-DVVAIA----Q------
	Kocuria atrinae	515564993	---Q------T------R-AATM		-HMD-LHLAY---S-A--TA
	Kutzneria albida	644680641	---T------L-----S--PLST		AE-D-SRLLV--VS-A-VAS
	Leifsonia aquatica	545652749	---S------L--------EL-Q		DP-D-SLLLI--LS-A--AG
	Mycobacterium yongonense	523908759	---R------L-----S--PLER		ES-D-SRLAV--VN-A-VAG
	Nocardia nova	644651698	---A----M-L-----S---LEH		EP-D--RLTV--VS-A-IAG
	Oerskovia turbata	663098971	--GT--N---A--------P-RH		EP-N-AALA--S-G------
	Promicromonospora sukumoe	518861377	--GT--N---V--------PVAR		ED-D-AALA--S-Q------
	Rathayibacter toxicus	653814453	---S------L--------ALEK		EP-D--RLLIE-VS-A-VAG
	Rhodococcus erythropolis	527105325	--GL------M------Q-PLEN		KP-D-LA-AA-SVQAAR-IA
	Solirubrobacter soli	654596529	--G-------T------V-EAEH		GA-D-SVLA---V--AR-T-
	Tetrasphaera japonica	665505523	---S------L--------PLAR		DE-D--ILAV--VS-A--AS
	Varibaculum cambriense	566218164	--GL------K--------K-KM		EA-D-YRIAAAG-L--S--S
	Xylanimonas cellulosilytica	502643283	--GL--N---V--------PLRT		DR-D-VAMAD---Q--V---

Supplementary Figure S16

Partial sequence alignment of histidine kinase showing a 1 aa insertion that is specific for *Bifidobacteriales*. Although not shown, this CSI is present in all *Bifidobacterium* species and subspecies.

			353		394
Bifidobacteriales	Bifidobacterium adolescentis	489905284	KEELLTKAEALVPVKDEKAAKQA	RQA	LAAIQEEWDQIGYVPR
	Bifidobacterium angulatum	489922339	-----K--------A--------	---	-----D----------
	Bifidobacterium animalis	490328693	--A--A--------ETGEQ----	-VK	--Q-------------
	Bifidobacterium asteroides	504834573	--A-VA-----L----T------	--E	--K------A--F---
	Bifidobacterium bifidum	489909129	-----K--------A--A---K-	---	-G---D----------
	Bifidobacterium boum	651390337	-----KQ-------A--------	--K	--E--D----------
	Bifidobacterium breve	489926974	--A--V---------TDAE--K-	--E	--K-------------
	Bifidobacterium catenulatum	489933231	--A--V-----------ES----	---	----------------
	Bifidobacterium choerinum	639202407	--A--V-----------AE--K-	--Q	--E-------------
	Bifidobacterium dentium	489937462	-----V-----------E-----	---	----------------
	Bifidobacterium gallicum	493337861	-----K-----L--TT-AQ----	---	--G-------------
	Bifidobacterium longum	494112423	--A--V---------TDAE--K-	--E	--K-------------
	Bifidobacterium magnum	551238870	--A--AQ-Q--L--NNAQE----	--K	----------------
	Bifidobacterium minimum	551240123	--A--AQ-------A--A---K-	-KS	--D---K--E------
	Bifidobacterium moukalabense	575769703	-----V-----------E-----	---	--S-------------
	Bifidobacterium pseudocatenulatum	490334243	-----V---------S-EE----	---	----------------
	Bifidobacterium pseudolongum	651883633	-----V----------AAQ--K-	--Q	--E-------------
	Bifidobacterium ruminantium	651886768	------------------E----	---	----------------
	Bifidobacterium subtile	639063802	-----K---T------A------	---	------Q--E------
	Bifidobacterium thermacidophilum	657872529	-----K--------A-A------	--K	--E--G----------
	Bifidobacterium thermophilum	505263545	-----K--------A-A------	--K	--E--G----------
	Bifidobacterium tsurumiense	651882900	-----K-----L--A--E-----	---	-SS-------------
	Bifidobacterium actinocoloniiforme	705420482	--A---R----L-----S-----	---	--R-------------
	Bifidobacterium aesculapii	943595969	-----K--------A------R-	--E	--K-------------
	Bifidobacterium biavatii	705394130	-----A--------A--A---K-	---	----------------
	Bifidobacterium bohemicum	705453664	-----K--------A--A---K-	--T	--S---Q---------
	Bifidobacterium bombi	763214659	-----K-----L--V--Q-----	---	--T--D----------
	Bifidobacterium callitrichos	759443331	-----V--------A------H-	--E	--K-------------
	Bifidobacterium coryneforme	797149044	--A--------L----V------	---	--S-------------
	Bifidobacterium crudilactis	736121057	--A--V-----L-----ES----	--S	--K------L------
	Bifidobacterium cuniculi	705447511	---T-TE----	--Q	----------------
	Bifidobacterium gallinarum	705425348	-----KQ-------A--A---K-	---	----------------
	Bifidobacterium indicum	917502482	--A--------L----V------	---	--S-------------
	Bifidobacterium kashiwanohense	746132243	--A--V-----------ES----	---	----------------
	Bifidobacterium merycicum	705459263	--A--K--------A--------	---	--E--D----------
	Bifidobacterium mongoliense	705436628	--A---R---------LPS--R-	---	-G---D---E------
	Bifidobacterium psychraerophilum	705398363	--A--V-----L---N-ES----	--S	--Q------L------
	Bifidobacterium pullorum	759447470	-----KQ-------A--A---K-	---	----------------
	Bifidobacterium reuteri	763216184	-----V---------TDDE--K-	--E	--K-------------
	Bifidobacterium saeculare	672994147	-----KQ-------A--A---K-	---	----------------
	Bifidobacterium scardovii	705450880	---A--A---K-	-E-	--S-------------
	Bifidobacterium stellenboschense	736513932	--A--V--------A------H-	--E	--K-------------
	Bifidobacterium stercoris	673001690	-----------------------	---	----------------
	Gardnerella vaginalis	523608730	--A--V-----L-----E---A-	-K-	-SE------K--F---
	Scardovia inopinata	493336197	--A--A--QQ-L--TTVHQ----	---	-GK--D---SV-F---
	Scardovia wiggsiae	494248955	-----V--Q-----NTAEQ----	---	--R--DD--S--H---
	Parascardovia denticolens	493332155	--A--E--K--L----V-E---T	--S	--K--D----------
	Alloscardovia criceti	516878185	--A--V--------A-V----A-	-I-	-GE--D---G--R---
Other Bacteria 0/500	Brevibacterium album	656050656	--A--AE-RG-L----LERT-A-		-RGL--R-EDA-R---
	Cellulomonas fimi	503537103	--A--AE---IL--R-LAS----		-RS--DR-EAA-K---
	Arthrobacter crystallopoietes	491409210	--V-VAE-Q--L-IE-LG--RK-		-QS-RDR-EE--K---
	Intrasporangium calvum	503258015	-----VE----L--T-LER--AQ		-R---DK--AA-K---
	Janibacter hoylei	495202275	--G--AE-Q--L----LDKT-A-		-RS--DR-EAA-K---
	Kineosphaera limosa	493639635	------Q-QQ-L--RNVES--A-		-RTL--K--AA-K---
	Oerskovia turbata	663110302	--A--EQ--K----T-LAQ--V-		-RD---K-EDA-K---
	Pseudoclavibacter soli	654790627	--Q--V-----L--T-WE--RAS		-RR--D---AA-K---
	Sporichthya polymorpha	655914859	--A-VEE--R-L--T-AGS-RK-		-RG---R--R--SL--
	Streptomyces varsoviensis	664352006	----VIE--K-L--S-L-S-RA-		FRS-N-R-EA--H---
	Thermomonospora curvata	502615550	--Q--VE--R-L--R-VRQ-R--		-RS---R--AA-P---
	Xylanimonas cellulosilytica	502642020	--AI--E---------LA---T-		-RG---R-EAA-K---
	Frankia alni	499921452	--K--AE-S--D-TDT-RSLRR		-REL-DR--E--R---

Supplementary figure S17

Partial sequence alignment of DNA repair ATPase showing a 3 aa insertion that is specific for *Bifidobacteriales*. Although not shown, this CSI is present in all *Bifidobacterium* species and subspecies.

			10		60
	Bifidobacterium adolescentis	547072106	PGIAQGLVEPKDIVADLVVGYSGAGKNLK	R	TNLLAAEAFGSALPYSVGGTH
	Bifidobacterium angulatum	489923522	---------S-S-----------------	-	--------LQA---G-----
	Bifidobacterium animalis	490328626	-A------DT-S-----A-----------	-	-----S--M---S-------
	Bifidobacterium asteroides	504834965	-AL-A---QTD-L----A-------RSSG	-	MD----Q-IN--H----A---
	Bifidobacterium bifidum	489910115	-A--E----RR-L----------------	-	------------V--G-----
	Bifidobacterium boum	651391446	----E----STG-----A-----------	-	---------------G-----
	Bifidobacterium breve	489925449	----E------------------------	-	--------LQ----------
	Bifidobacterium catenulatum	489931298	--V-------V------------------	-	--------------------
	Bifidobacterium choerinum	639202464	-A--EN--V-HT-----A--------S--	-	-----S--LN--T-------
	Bifidobacterium dentium	489936572	--V----I---------------------	-	--------------------
	Bifidobacterium gallicum	493337776	--V-E-----TS--S--A-----------	-	M-----------T--G-----
	Bifidobacterium longum	494110388	----E----SQ------------------	-	--------LQ---------K-
	Bifidobacterium magnum	551238932	-AM-N---DASTL----A-----------	-	-----S--IN--V-------
	Bifidobacterium minimum	551240152	-AVHD--IDERG--------------S--	-	-G------L---V--G-----
	Bifidobacterium moukalabense	575770559	--V----I---------------------	-	A-------------------
	Bifidobacterium pseudocatenulatum	490332944	----------A------------------	-	V-------------------
	Bifidobacterium pseudolongum	651883579	----NN--V-HT-----A-----------	-	-----S--LN--V--A-----
	Bifidobacterium ruminantium	651886317	---------S-------------------	-	--------------------
	Bifidobacterium subtile	639064500	-A--H---D--G-----A--------S--	-	-D---S------HA-G---S-
	Bifidobacterium thermacidophilum	657872467	----E--I-HTG-----A-----------	-	------------V--G-----
	Bifidobacterium thermophilum	505264178	----E--I-HTG-----A-----------	-	------------V--G-----
Bifidobacteriales	Bifidobacterium tsurumiense	651882205	-ALSA--- TG-V-V--A----------S	K	PS----Q-----S------K-
	Bifidobacterium aesculapii	943596608	--V-E----H---I---------------	-	--------L---V--G-----
	Bifidobacterium biavatii	705394675	--V-E--I---SV----A-----------	-	--------L---V--G-----
	Bifidobacterium bohemicum	737153220	-AV-E---DYG--L---A---------P-	-	PG----------T------V-
	Bifidobacterium callitrichos	759444384	--V-E-------L----------------	-	--------L-----G-----
	Bifidobacterium coryneforme	705403285	-AVSA---RTD-L------A------TPG	-	VD----Q-LN--H--G---A-
	Bifidobacterium crudilactis	736120506	-AV-T---DMHS---N-------------	-	------Q-----T--G---S-
	Bifidobacterium cuniculi	705445215	-AV---I-D--S---T-A--------S--	-	-D---S---N--V-------
	Bifidobacterium indicum	705388441	-AVSA---RTD-L------A------TPG	-	VD----Q-LN--H--G---A-
	Bifidobacterium kashiwanohense	746133239	--V-------T------------------	-	--------------------
	Bifidobacterium merycicum	705456846	-------I---SV----------------	-	--------LQA---G-----
	Bifidobacterium mongoliense	705436706	-----D-I-RHGV----A-------RKTG	-	M-----------Q--G---V-
	Bifidobacterium psychraerophilum	705400932	-ALVA---DAQE---N----------S--	-	--------L---T--G-----
	Bifidobacterium pullorum	705440522	-AV-EN-I--T------------------	-	A-----------V-------
	Bifidobacterium reuteri	763216124	-A--E---DS-------------------	-	-----S--LQ-----A-----
	Bifidobacterium stellenboschense	736510229	--V-E----HT------------------	-	---------------G-----
	Bifidobacterium gallinarum	672966253	-AV-E----TT------------------	-	A-----------V-------
	Bifidobacterium saeculare	672991646	-AV-E----TT------------------	-	A-----------V-------
	Bifidobacterium scardovii	672996798	--V-E---D-----V--------------	-	------------A-------
	Bifidobacterium stercoris	673003118	---------S-------------------	-	--------------------
	Alloscardovia omnicolens	545373635	-AL-A--- NSQ-S-HA--------RKTG	-	V----S--L-N-TA---A-S-
	Alloscardovia criceti	516877329	-A-SA--- DTN-T-N------------G	-	I-----Q-L---TA-A---K-
	Actinobaculum massiliense	494059767	-AV-A--TDGTG-T-T-A---------TP-		P--IFS--IE--A--K-----
	Arsenicicoccus bolidensis	656265464	--F-A--L-GD-V-VVAAS-T-----G--		PH--G---M-AMS--G---S-
	Brachybacterium faecium	506256407	-LVRA---DAGHLS-V-P-------RSA-		QH--FS--S---S--A-----
	Cellulomonas fimi	503536743	----A-V---R-V--V-A-------RT--		-H---S-GL---Q--A-----
	Demetria terragena	648489172	-AFEG---QTD-V-VVAAS-T-----A--		PH--GSQVM-AMS--G---G-
	Geodermatophilaceae bacterium	652450108	-AL-A--- TP-V-VVAAS-T-----SA-		-H--GS-VM---SA-G---V-
	Humibacter albus	551281553	--V-A---DET----V-A--P--------		-TY--S-LL-N-V--G-----
Other Bacteria 0/500	Isoptericola variabilis	503604423	--V-A---S-V-L--V-AN-------A--		PH---S--L-A-A--A-----
	Jiangella gansuensis	652482365	--L-A-V-A-D-V--V-AC-T-----SV-		PH---S-IM-A-S--A---V-
	Kineococcus radiotolerans	501035095	--L-A--L--A-V-VVAAS-T-----S--		PH--G--VM--MS--G---G-
	Mobiluncus curtisii	490107296	-AVWA---DAS-L--N-A---------AM-		SH---VS-L-N-QG-AMA-V-
	Nocardiopsis potens	516175794	-AL-E--ASAE-L-VVA-T-T----RS--		P---GS-TM--VA--G---V-
	Oerskovia turbata	663109348	--V-A--LS-V-V--V-AN-------S--		-H---S--L---Q--A---S-
	Pseudoclavibacter soli	654790746	-AV-A----LQ-L--V-S--P----RS--		P----S-IM-A-SA-A---V-
	Rhodobacter capsulatus	502831321	-L-EA-VIDLDE--I--KA-V----RS--		E---H--LA-GTM---A--K-
	Sanguibacter keddieii	502630595	--V-A--LSTD-L-TV-A---------S--		NH---S--L---Q--A-----

Supplementary figure S18

Partial sequence alignment of n-acetyl-gamma-glutamyl-phosphate reductase showing a 1 aa insertion that is specific for *Bifidobacteriales.* Although not shown, this CSI is present in all *Bifidobacterium* species and subspecies.

```
                                                         1                                            42
Bifidobacteriales
Bifidobacterium adolescentis          547072098   MSVTFAQGFSAAGVAAGISA V EGKKDLALVVNNGPLDAAAGV
Bifidobacterium angulatum             489923521   --------------E----S - Q--------------------
Bifidobacterium animalis              490328624   ------K--E----V----S Q T--Q-V---------N-----
Bifidobacterium bifidum               489910114   ------K------------S I -----------Q----S----
Bifidobacterium boum                  651391445   --------------V----S K A--Q---F-------------
Bifidobacterium breve                 489925452   --------------V----S - -----------------V---
Bifidobacterium catenulatum           489931300   --------------E----- - ---------------------
Bifidobacterium choerinum             639202465   ---------T----V----S - -----------D---K-----
Bifidobacterium dentium               489938295   -------------------- - -----M-------------A-
Bifidobacterium gallicum              493337775   ---------R----E----- - A----V-----D--IN-----
Bifidobacterium longum                494110387   -------------------S - ---------------------
Bifidobacterium minimum               551240153   --------------V----S - P--R-V-V---D--R------
Bifidobacterium moukalabense          575770558   -------------------- - -------------------A-
Bifidobacterium pseudocatenulatum     490332941   --------------E----- - ----------------M----
Bifidobacterium pseudolongum          651883578   --------------E----- T P----V-----E---T-----
Bifidobacterium ruminantium           651886318   --------------S----- - A--------------------
Bifidobacterium subtile               639064503   ----Y----K----D----- K P------F---E-----V---
Bifidobacterium thermacidophilum      657872468   -------------------- K A--L---F-------------
Bifidobacterium thermophilum          505264177   -------------------- K A--L---F-------------
Bifidobacterium tsurumiense           651882204   ----Y---------V--V-S - A----M-V---E-----C---
Bifidobacterium aesculapii            943596607   ---------A----E----- - A--------------------
Bifidobacterium asteroides            799166503   --I-Y-A--QTGTAI-HRPG G SRRRN------K--M-T-V--
Bifidobacterium biavatii              705394533   --------------V----- - ---------------------
Bifidobacterium callitrichos          759444385   -------------------S - -D------I------------
Bifidobacterium coryneforme           799123583   ----Y-A--Q-GTAK-HR-G G SER-N-TMI--Q--M-T-V--
Bifidobacterium crudilactis           736120507   ----Y-K--E----S----- H -----------Q---H-----
Bifidobacterium dentium               489938295   -------------------- - -----M-------------A-
Bifidobacterium gallinarum            705424157   ------K--R----E----- - D----------D-----M---
Bifidobacterium indicum               705388438   ----Y-A--Q-GTAK-HRTG G SER-N-TMI--Q--M-T-V--
Bifidobacterium kashiwanohense        705411905   --------------E----- - ---------------------
Bifidobacterium magnum                551238933   -T---PS----S--C-R--- - P--------M-E--YWN----
Bifidobacterium merycicum             705456842   --I-----------E----S - K--------------N-----
Bifidobacterium mongoliense           705436708   ----Y----R----Q----- - -S-R---M---D---Q-----
Bifidobacterium psychraerophilum      705400645   ----Y----A----P----S I A------V-T-Q----V----
Bifidobacterium pullorum              759447322   ------K--R----E----- - D----------D-----M---
Bifidobacterium reuteri               763216123   --------------Q----- - AD-------------------
Bifidobacterium saeculare             705432208   ------K--R----E----- - D----------D-----M---
Bifidobacterium stellenboschense      736510231   ---------A-S--E----- - A--------------------
Bifidobacterium scardovii             672996797   -------------------S - A--------------------
Bifidobacterium stercoris             673003119   -------------------- - ---------------------
Alloscardovia omnicolens              551236028   ---V----------RG---S - -----V-V-------NV----
Alloscardovia criceti                 516877326   ---V------T---RG---- I ----------------V----
Other Bacteria 0/500
Catenulispora acidiphila              506274283   ----S-K--R-S-I----KD   S-NP-------D---T-----
Cellulosimicrobium cellulans          640257610   -T--TP---R-------LKS   T-A--V-----D----V--A-
Jonesia quinghaiensis                 656029677   ----Y-R--T-------LKN   S-AQ-V-----A----T-SA-
Actinomadura madurae                  545321527   ----APR--R----V--LKD   S-SR-------D--SR-----
Actinomyces viscosus                  489882751   ----A----R----R--LK-   S--P-------D----T----
Agrococcus lahaulensis                651234018   ----A-A--R-------VKS   S-GA-VT----D--I------
Amycolatopsis alba                    522118430   -T--QPK--R--------K-   ---L-------D---QV----
Arsenicicoccus bolidensis             656265463   ----A----R----T--LK-   S-RP-V-----D--QH---A-
Austwickia chelonae                   493547466   -----PA--R----T--LKL   S-AA-V-----D---YS----
Microbacterium indicum                656173520   ----APR--E-------LKS   T-RN-V-V---R---KTG-A-
Microtetraspora glauca                663716881   ----A----T---I----KQ   N-NP-------T--RR-----
Nocardiopsis alkaliphila              516197782   ----AP---R---I---VGS   Q-RR-V-----D--SR--GAI
Oerskovia turbata                     663109347   -T--GPL--R-------LKS   T----V-----E----V--A-
Paraoerskovia marina                  656320687   ----A----R-T--V--LKS   T-ER-V-----D--EL-----
Saccharomonospora azurea              491591341   -T--APK--R-------LKS   S--P-V-----D--W-V--A-
Streptomyces zinciresistens           494761195   ----A-K--T---I----KE   N-NP-------E--RR-----
```

Supplementary figure S19

Partial sequence alignment of arginine biosynthesis bifunctional protein ArgJ showing a 1 amino acid insertion that is specific for *Bifidobacteriales*. Although not shown, this CSI is present in all *Bifidobacterium* species and subspecies.

Group	Organism	ID	103		150
Bifidobacteriales	Bifidobacterium longum	494111998	LGLAEAPLRIECYDISNT	V	GGAFQVASMVVFEDAIAKKSEYRRFAIRG
	Bifidobacterium adolescentis	547074632	---EQ-------------	-	-----------------------------
	Bifidobacterium angulatum	489922916	---E--------------	-	---------------V-------------
	Bifidobacterium animalis	490328460	---SQ-------------	-	--------------GM-R-----------
	Bifidobacterium asteroides	504834739	---DR-------------	-	--Q------------VP------------
	Bifidobacterium bifidum	489909868	----Q-------------	-	-----------------------------
	Bifidobacterium boum	651391049	---PQ-------------	-	---------------------------K-
	Bifidobacterium breve 31L	584611288	---NQ-------------	-	-------------------------S---
	Bifidobacterium catenulatum	489931497	---EQ-------------	-	-----------------------------
	Bifidobacterium choerinum	658450183	---DQ-------------	-	---Y----------GV-------------
	Bifidobacterium dentium	489940303	---EQ-------------	-	-----------------------------
	Bifidobacterium gallicum	493337177	------------------	-	---Y-----------M-------------
	Bifidobacterium moukalabense	575770080	---EQ-------------	-	-----------------------------
	Bifidobacterium pseudocatenulatum	490332651	---EQ-------------	-	-----------------------------
	Bifidobacterium pseudolongum	651883376	---DQ-------------	-	--------------G--------------
	Bifidobacterium subtile	639063903	---KQ------G------	I	--V--------------------------
	Bifidobacterium thermacidophilum	657872125	---PQ-------------	I	---Y-------------------------
	Bifidobacterium thermophilum	505263502	---PQ-------------	I	---Y-------------------------
	Bifidobacterium tsurumiense	651882164	---SQ-------------	-	A-------------G--------------
	Bifidobacterium actinocoloniiforme	917298552	---KQ-------------	-	---------------V-------------
	Bifidobacterium aesculapii	943597287	------------------	-	---------------V-------------
	Bifidobacterium biavatii	705395314	---SQ-------------	-	---Y-------------------------
	Bifidobacterium bohemicum	705453898	---SQ------G------	I	--IY-----------V-------------
	Bifidobacterium bombi	763213928	---KQ------G------	I	--I-----------GV-------------
	Bifidobacterium callitrichos	759441645	---D--------------	-	---------------V-------------
	Bifidobacterium coryneforme	799123300	---DK-------------	-	--Q-----------G-P------------
	Bifidobacterium crudilactis	736120768	---DK-------------	I	A--------------V---------S-K-
	Bifidobacterium cuniculi	917313818	---QQ-------------	-	---Y----------G--------------
	Bifidobacterium gallinarum	917310270	---D--------------	-	--------------G--------------
	Bifidobacterium indicum	705387990	---DK-------------	-	--Q-----------G-P------------
	Bifidobacterium kashiwanohense	757808365	---EQ-------------	-	-----------------------------
	Bifidobacterium magnum	672972440	---SQ-------------	-	D-SY--G--------V----------V--
	Bifidobacterium merycicum	705458811	---N--------------	-	---------------V-------------
	Bifidobacterium minimum	672977296	-N-DH------------A	T	D-SY--------------P-L----E-H-
	Bifidobacterium mongoliense	705436925	---KQ-------------	I	---------------V-R-----------
	Bifidobacterium psychraerophilum	672985945	----Q-------------	L	---Y-----------T---------S---
	Bifidobacterium pullorum	917305364	---D--------------	-	--------------G--------------
	Bifidobacterium reuteri	763215765	--MS--------------	-	-----------------------------
	Bifidobacterium ruminantium	705404810	---EQ-------------	-	---Y-------------------------
	Bifidobacterium saeculare	917309243	---D--------------	-	--------------G--------------
	Bifidobacterium scardovii	705446616	---KQ-------------	-	-----------------------------
	Bifidobacterium stellenboschense	917316020	---D--------------	-	---------------V-------------
	Bifidobacterium stercoris	673003370	---EQ-------------	-	-----------------------------
	Alloscardovia criceti	516878324	-M-DN-------F----A	S	--------------GM---NF--H-----
	Alloscardovia omnicolens	545375555	-M-DK-------F----A	S	--S-----------G----NY--H-----
	Parascardovia denticolens	493332059	---K-------A-----A	S	--GY----------GAP--K---H-S---
	Gardnerella vaginalis	532642999	-SMS-------------S	A	D-RY----------G---P------S-K-
	Scardovia inopinata	493336094	-D---------------A	S	--GY----------GM----Q--H-----
Other Bacteria 0/500	Scardovia wiggsiae	494249140	---K-------------A	S	--GC----------G--R-K---H-----
	Actinotalea ferrariae	601043275	-D-PT-----------T-		Q-TH--G-------GL-RR----H-VV--
	Catenulispora acidiphila	506274426	I-MDQ-----------HL		Q-TNV---------GLPR-------T-K-
	Granulicoccus phenolivorans	652533503	---P------------HL		Q-TEV---------GLPR-------I-K-
	Jonesia denitrificans	506251761	-E-S----------V-HN		Q-T--T--------GLP---A--L-H-K-
	Kribbella flavida	502685306	-D-P-----L----V--L		Q-TEV---------GLPR-------V---
	Microlunatus phosphovorus	503628234	-E-PS------------L		Q-TEV---------GLPR-------I--S
	Mycobacterium avium	489969288	---S--------V---HV		Q-TDV-G-L-----GLPR--D--H----E
	Nocardioides insulae	655250070	-E-G-V-----------L		Q-TEV---------GLPR-------V---
	Paraoerskovia marina	656321354	-E-DT-----------T-		Q-TH----------GL-R-G---H--V--
	Promicromonospora sukumoe	518858895	-D-DS-----------H-		Q-T--S--------GL-R-----S-S---
	Sanguibacter keddieii	502630373	-D-DS-----------T-		M-TH-A--------GL-R-----T-GVK-
	Thermobispora bispora	502897266	---EQ-------F-V-H-		Q-SDV-G-------GL-R--------V-N
	Trueperella pyogenes	640530175	---ER------G----H-		Q-TN--G-------GL----A--HYI---

Supplementary figure S20

Partial sequence alignment of excinuclease ABC subunit C showing a 1 aa insertion that is specific for *Bifidobacteriales*. The presence of this indel in a strain of *C. trachomatis* (HiIMS) is possibly due to spurious causes. Although not shown, this CSI is present in all *Bifidobacterium* species and subspecies.

Group	Species	ID	54	Insert	105
Bifidobacteriales	Bifidobacterium adolescentis	500063210	HRGAHELAARSTMAFEDARAKVAKLVGADS	AEGE	EEIVVTAGATAGLNLLAT
	Bifidobacterium angulatum	489923132	-----------------------R----NA	E---	------------------
	Bifidobacterium animalis	490328383	-----T--GL--V---Q------T----SA	E--N	--L---M---D---T---
	Bifidobacterium asteroides	504834798	----------------H--SR--A----EG	Q--G	-----------A------
	Bifidobacterium bifidum	489913498	---------L-------------R----NA	E--N	------------------
	Bifidobacterium boum	651391140	----------------E------R----N-	E---	------------------
	Bifidobacterium breve	489925914	------------V---E-----------NT	----	--V---G-----------
	Bifidobacterium catenulatum	489931782	----------------E------R----ST	V---	------S-----------
	Bifidobacterium choerinum	639202986	-----T------V---Q------R----S-	D--G	--L---M---DA--M---
	Bifidobacterium dentium	489937025	----------------E----M-R----NA	----	--V---S-----------
	Bifidobacterium gallicum	493337092	-----------------------R----NA	Q---	--L---S-----------
	Bifidobacterium longum	494112595	----------------E-----------NA	----	--V---G-----------
	Bifidobacterium magnum	551239341	------------L------VS-S-------	E--S	--L---------------
	Bifidobacterium minimum	551240338	----------------H---S--A-----T	R--Q	------------------
	Bifidobacterium moukalabense	575769993	----------------E----M-R----NA	T--G	------S-----------
	Bifidobacterium pseudocatenulatum	490332310	----------------E------R----N-	----	------S-----------
	Bifidobacterium pseudolongum	651883427	------------V-------AM-RF---SA	E--H	--L---------------
	Bifidobacterium ruminantium	651886204	----------------Q-----------N-	----	------S-----------
	Bifidobacterium subtile	639061761	---------L--E---T-------------	E--H	------------------
	Bifidobacterium thermacidophilum	657872216	------------L---E------R----N-	E---	------------------
	Bifidobacterium thermophilum	505263397	----------------E------R----N-	E---	------------------
	Bifidobacterium tsurumiense	651882287	----------------E--G---A----SA	E--N	------S----------A
	Bifidobacterium actinocoloniiforme	705419057	-------------D--R--SQI-R-----A	R--H	------------------
	Bifidobacterium aesculapii	943596827	------------V---R------R----NA	E--A	------G-----------
	Bifidobacterium biavatii DSM 23969	672954906	------------V---E------R----NA	E--Q	--V---G-----------
	Bifidobacterium bohemicum	705454012	------------V---E---RM-RF----D	G---	------------------
	Bifidobacterium bombi	763214397	------------T----------RF---NY	-D--	Q---I------S------
	Bifidobacterium callitrichos	759442064	----------------------------NA	T---	------------------
	Bifidobacterium coryneforme	799123386	------------L---ES----------E-	QD-C	----I------A------
	Bifidobacterium crudilactis	736122283	----------------E--G-I-R--N--A	D---	----F------S------
	Bifidobacterium cuniculi	705446896	------------E---Q------R------	T--S	--L-------------T-
	Bifidobacterium gallinarum	705427009	------------V---R---SM-R----CA	D--G	------G-----------
	Bifidobacterium indicum	705388085	------------L---ES----------E-	QD-G	----I------A------
	Bifidobacterium kashiwanohense	705412365	----------------E------R----NA	V---	------S-----------
	Bifidobacterium merycicum	705460324	------------T---------------NA	E---	------------------
	Bifidobacterium mongoliense	705434838	------------V----------R-----A	EPDH	------S----A------
	Bifidobacterium psychraerophilum	705400467	------------V---E------R--H-SE	----	----F------S------
	Bifidobacterium pullorum	705442509	------------V---R---SM-R----CA	D--G	------S-----------
	Bifidobacterium reuteri	763215883	----------------E-----------NA	----	------G-----------
	Bifidobacterium saeculare	705432435	------------V---H---SM-R----CA	D--G	------G-----------
	Bifidobacterium scardovii	705447551	------------T----------R----NA	----	------------------
	Bifidobacterium stellenboschense	736512067	----------------E-----------NA	E--N	--V---G-----------
	Bifidobacterium stercoris	673003500	------------------------------	----	------------------
	Gardnerella vaginalis	657897421	---------L--V---E-----------C-	E--N	---I--P---GA------
	Scardovia inopinata	493336053	-----A---E--E-Y-A--GDI-R----N-	QP-Q	------S-----------
	Scardovia wiggsiae	494249222	------------Q-Y----TR--HF---Q-	QS-A	------P-----------
	Alloscardovia criceti	516878426	--------SL--D-Y-NG-H-I-Q----HY	G--D	D-------------M--N
	Alloscardovia omnicolens	545375297	--------SL--D-Y-NG-YA--R-I--HY	G--S	D-----G-------M--N
	Parascardovia denticolens	493332015	------------E---A---Q--S-----C	EP-A	--L--------S------
Other Bacteria 0/500	Hyphomicrobium nitrativorans	563761452	--A-------A-D-Y-G--Q---HFL--SG	P	---IF-R---EAI--V-A
	Lachnoclostridium phytofermentans	501155088	-----T---TA-D-Y-A--N-IKHFIN-S-	Y	---IFVR---E-I--V-A
	Alistipes shahii	505359488	---V-F-SEEA-VLY-A--ERI-AF---AA	K	----F------S--TV-Y
	Nocardia brasiliensis	504796942	--A---------D-Y-Q--DL--RFI--A-	A	---IFVR---E-I--I-Q
	Photobacterium phosphoreum	595580911	---S-S-T-NA-L---Q---T-Q-FI--A-	H	K--IW-R---EA---I-Q
	Rhodomicrobium vannielii	503185025	------------D-Y-H--Q---GFI--N-	P	---IFVR---E-I--V-K
	Synechococcus elongatus	499563880	---V-T-SGKA-D-Y-G--Q---RFIN-KT	-	Q---Y-RN-SEAI--V-Y
	Thioclava dalianensis	661437848	---L-T-SNIA-EKY-GT-----RFL-VKD	-	----F-T-T-E-I--VSY
	Treponema primitia	497948445	------------D-Y----G---RFL--P-	K	DN--FVR-T-E-I--V-Q
	Vibrio tasmaniensis	515674032	---S-S-T-QA-SQ--A--D---QFI--T-	S	K--IW-R---EA---I-Q
	Xylanimonas cellulosilytica	502641708	-----F--EEA-S---E--TA--AF----D		D---W-S----AI--V-Y
	Sanguibacter keddieii	502630556	-----Q--EEA-L-------Q--TF----E		D---W-SN---AI--V-Y
	Promicromonospora sukumoe	518857055	-----Q--EEA-E---Q---S--AF----E		G---WQP----AI-VV-Y
	Microbacterium yannicii	518011277	-----T---EA-EL------T--GF---RP		-QL-W-S--------I-Y
	Aureococcus anophagefferens	323455939	-----A--T-A-D-Y-A--D---AF---RR		D---W-R---EAI--V-Q
	Beutenbergia cavernae	506363108	-----A--EEA-V---A--TQ--A---V-A		G---W-KN--E-I--V-Y
	Kocuria palustris	493243897	-----T--VEA-D------RT--GF---QE		----W-SN--E-----TY

Supplementary figure S21

Partial sequence alignment of cysteine desulfurase showing a 4 aa insertion that is specific for *Bifidobacteriales*. Although not shown, this CSI is present in all *Bifidobacterium* species and subspecies.

Group	Species	Accession	58		81
Bifidobacteriales	Bifidobacterium adolescentis	489906135	DIGMLFGVG	S	DSAGAGKHGDEMLR
	Bifidobacterium angulatum	489923421	---S-----	K	T-R---M--AD---
	Bifidobacterium animalis	490328284	---T-----	A	--R---MR-EA---
	Bifidobacterium asteroides	504834861	---T---L-	-	QAP---M--AA--E
	Bifidobacterium bifidum	489909294	---G-----	A	-AP---M--CD---
	Bifidobacterium boum	651390684	---T-----	G	------Q--GP--E
	Bifidobacterium breve	489925572	---S-----	-	HAH---M--SD---
	Bifidobacterium catenulatum	489931913	---T-----	-	Q-D------A---Q
	Bifidobacterium choerinum	658450165	--T-----	A	A-R---M---M--A
	Bifidobacterium dentium	489938860	---T-----	A	N-D------A----
	Bifidobacterium gallicum	493338889	---SY----	A	--Q---RT-RS--Q
	Bifidobacterium longum	658453823	---S-----	A	-AH---R--VD--Q
	Bifidobacterium magnum	551238798	---------	-	A-R---MR-TV--E
	Bifidobacterium moukalabense	575769894	---T-----	A	--D---R--A---Q
	Bifidobacterium pseudocatenulatum	490332156	---T-----	-	Q-D------A----
	Bifidobacterium pseudolongum	651888501	---T-----	E	S-Q---MS-TA--T
	Bifidobacterium ruminantium	651886161	---------	A	------A-------
	Bifidobacterium thermacidophilum	657871747	---T-----	A	------A--TD---
	Bifidobacterium thermophilum	505263295	---T-----	A	------A--TD---
	Bifidobacterium tsurumiense	651882347	---TV----	-	HAR---M--VQ--G
	Bifidobacterium aesculapii	943596542	---SM----	A	TAH---MY-AD-I-
	Bifidobacterium callitrichos	759443353	---S-----	-	NAH---M--VA---
	Bifidobacterium coryneforme	799123449	---SH--Q-	P	Q-K---M--A----
	Bifidobacterium crudilactis	736122311	---SF----	P	T-K-S-M--RD-I-
	Bifidobacterium cuniculi	705443930	---S-----	P	Q-L-S-MR--A--
	Bifidobacterium gallinarum	705425035	---T-----	-	------Q--GA---
	Bifidobacterium kashiwanohense	746132457	---T-----	-	Q-D------A----
	Bifidobacterium psychraerophilum	705398814	---SF----	P	Q-K--HM--RD-I-
	Bifidobacterium pullorum	759446967	---T-----	-	------Q--GA---
	Bifidobacterium reuteri	763216231	---S-----	A	AAH---M--RD--
	Bifidobacterium saeculare	705430810	---S-----	-	------Q--GA---
	Bifidobacterium saguini	727803170	---S-----	A	-AH---M--VD--
	Bifidobacterium scardovii	673000024	---S-----	A	------R--AD---
	Bifidobacterium stellenboschense	736510402	---A-----	-	-AH---M--AD---
	Bifidobacterium indicum	655535346	---SH--Q-	P	Q-K---M--A----
	Bifidobacterium stercoris	673000906	---------	-	--------------
Other Bacteria 0/500	Actinomadura atramentaria	648649855	-L-AV--TA		-PRWS-AS-TAL-T
	Actinomyces cardiffensis	492785742	EM-TV--TD		RPQWR-AS-EQF--
	Corynebacterium ciconiae	516649818	-L-TFV---		RAEYD-VS-EQL--
	Gordonia paraffinivorans	493955309	-V-SV--T-		RPEWE-VS-VA---
	Haloglycomyces albus	658648874	-L-SN--TS		RPEW-NAT-A-L--
	Humibacter albus	551280648	---SR--TA		-PRF--A--EVF-T
	Jiangella gansuensis	652483002	-L-AV--TD		RPEWS-AS-VAL-T
	Kutzneria albida	644672984	-L-AV--T-		-PRWS-A--V-L-A
	Lechevalieria aerocolonigenes	663693960	-L-AV--TS		-PRWS-A--VVL-T
	Microbacterium indicum	656174692	---SR--TA		RPEF--A--E-F--
	Mycobacterium tuberculosis	504397842	---EV---D		-PRWQ-VS-AD---
	Nonomuraea coxensis	522033448	-L-R---TA		-PRW--AT-VAL-E
	Propionibacterium acnes	488492655	---SV--TA		-PQW--AA-ADLVG
	Pseudonocardia acaciae	655570827	-L-SV--T-		-PRW--V--AA--A
	Spirillospora albida	663134258	-L-AV--TA		-PRWS-AS-RAL-A
	Thermomonospora curvata	502614142	-L-AV--TA		-PRWR-AS-T-L-A

Supplementary figure S22

Partial sequence alignment of 2-C-methyl-D-erythritol 2,4-cyclodiphosphate synthase showing a 1 aa insertion that is specific for *Bifidobacteriales.* Although not shown, this CSI is present in all *Bifidobacterium* species and subspecies.

Group	Species	ID	405		454
Bifidobacteriales	Bifidobacterium adolescentis	547072080	AEDRGQELWDLTDEDFINV	FKDFL	PADKAPQVREVLSTEGSVSARNGKGG
	Bifidobacterium angulatum	489923515	--G--V-----------ST	-AA--	--EV--K------SD---AS------
	Bifidobacterium animalis	490328615	--N--V-----S-D---EI	-RG-V	EP-V-VE--------------A----
	Bifidobacterium asteroides	504834958	--G--C--A--D-D--VR-	-A-WV	-VER--E--S--TAG-A-R--Q-Q--
	Bifidobacterium bifidum	489910085	--G-----R----D---GI	-R---	--------------------------
	Bifidobacterium boum	651391439	--G-S--------D---A-	-----	--------------K---T----H--
	Bifidobacterium breve	489925470	--G--------------ET	-AA--	--G---G------SH---DS------
	Bifidobacterium catenulatum	489931308	-------------D--VE-	--E--	------E-------------------
	Bifidobacterium choerinum	658449985	--S--E-----S-D---EI	-RG-V	APEQ-VK--------------A----
	Bifidobacterium dentium	489936585	-----------------E-	-----	--------------------------
	Bifidobacterium gallicum	493337768	--G----------D-Y-G-	-A---	---I-V---D----A---A-------
	Bifidobacterium longum	494112829	--G----------N---ET	-AA--	------G------SH---DS------
	Bifidobacterium magnum	551238939	--G-------------VET	-QE-V	APEL--E--G---AA----S------
	Bifidobacterium minimum	551240159	--S--V-------D-YRA-	-AGY-	DSES--R--Q---AT---A----R--
	Bifidobacterium moukalabense	575770552	--E--------------E-	-R---	--------------------------
	Bifidobacterium pseudocatenulatum	547837238	-------------D--VE-	--E--	------E-------------------
	Bifidobacterium pseudolongum	551237222	--G--E-------D---E-	-RE--	--EQ-VG-----------A--A----
	Bifidobacterium ruminantium	651886323	--------------N--S-	-----	--E-----------------------
	Bifidobacterium subtile	639064520	--E--G-----D-G--VEI	-SN--	------A------V----A-------
	Bifidobacterium thermacidophilum	657872474	--G-----C----D---S-	-SG--	--------------K---T----R--
	Bifidobacterium thermophilum	505264170	--G----------D---S-	-SG--	--------------K---T----R--
	Bifidobacterium tsurumiense	651882198	--Q--------S-A--A-I	-H---	D-QT-AG-----------N-------
	Bifidobacterium actinocoloniiforme	705420132	--Q--V-----S----QRA	-----	--PL-----Q----Q---Q--Q-Q--
	Bifidobacterium aesculapii	943596596	--G----------D--TST	-AA--	------G------SH---DS------
	Bifidobacterium biavatii	705394679	--G-------------V--	-----	---L----------------------
	Bifidobacterium bohemicum	705453853	--G-SV-------A---E-	-----	-PQV--K--T--TV-------D-E--
	Bifidobacterium callitrichos	759443428	--G--------------ST	-AA--	------G------SH---DS------
	Bifidobacterium coryneforme	799123572	--G--C-------Q---RE	-RNY-	-D-Q--K------AD---Q----R--
	Bifidobacterium crudilactis	736122110	--G-A------S----AD-	-----	--GQSAG--A---AQ--AD-------
	Bifidobacterium cuniculi	705445229	--T-HE-------D---EI	-H---	---Q-IG-----------A--A----
	Bifidobacterium gallinarum	705424141	--E----------A---T-	-EGY-	------------------A--R----
	Bifidobacterium indicum	917502370	--G--C-------Q---RE	-RNY-	-D-Q--K------AA---Q----R--
	Bifidobacterium kashiwanohense	547837238	-------------D--VE-	--E--	------E-------------------
	Bifidobacterium merycicum	705456822	--G--V-------D---TT	-AA--	--ET--K------SD---AS------
	Bifidobacterium mongoliense	705436720	--S--V--L--S-D--RTI	-H--V	-T-R--M-H-----S-----------
	Bifidobacterium psychraerophilum	705400629	--G-D------R-D--ADI	-----	--EQ-AD--A---AQ--ADS---Q--
	Bifidobacterium pullorum	705440508	--E----------D--VT-	-A---	------------------A--R----
	Bifidobacterium reuteri	763216111	--G--------------DT	-AA--	------G--Q---SH---DS------
	Bifidobacterium saeculare	705432190	--E----------A---T-	-EGY-	------------------A--R----
	Bifidobacterium saguini	727802989	--G----------Q---DT	-AA--	--E---G------SH---DS------
	Bifidobacterium stellenboschense	736510266	--G-------------KST	-AA--	--E---G------SH---DS------
	Bifidobacterium scardovii	672996789	--E-------------AG-	-----	-----AE-------------------
	Bifidobacterium stercoris	673003125	-------------------	-----	--------------------------
	Gardnerella vaginalis	532643121	--SLKCD-S--SND-YATI	LDGII	D-N-I-DL-L---SY----S-C----
	Alloscardovia criceti	516877314	--Q------------Y-SI	--E--	N--V----------Q---NS-L----
	Parascardovia denticolens	493332775	--K--V-------D-YARI	-SG--	---L----KTI-TVQ---A--Q-Q--
	Scardovia inopinata	493336820	--S--KD-A--S-S--ARL	-A-Y-	-EEQ-VT--D---VQ---N---QA--
	Alloscardovia omnicolens	545373668	--E--V-----S---YCGI	-QN--	S--V--G-------Q---N--A-Q--
Other Bacteria 0/500	Austwickia chelonae	493547462	C-K--I-----S-S-LFEI		S-HL--E--S---VS--LAS-DA---
	Brachybacterium paraconglomera	498235967	--EQDK-------DELAGI		SEHLD-S------V---IAS-DA---
	Brevibacterium album	656051432	--S--V-----S---YAGI		S-HLT-A-----T-H--IDS--A---
	Cellulosimicrobium cellulans	640257625	C-E--I-----S-D-LAGI		SEHLT-G--D---V---LAS-DAV--
	Intrasporangium calvum	503257727	C-S--I-----S----ARI		DPHLT-T--D---V---LAS-DAV--
	Isoptericola variabilis	503604430	C-E--I-----S-A-LAAI		SEHLT-E--S--TV----AS-DAV--
	Janibacter hoylei	495201164	C-E--I-----S---LAGI		SQHLT-D------VD--LAS-DA---
	Kribbella flavida	502687465	C-K--I---------LAAI		SPHLT-D--S---V-----S-D-R--
	Micrococcus luteus	488942580	--A--V--------EYAAI		SPALT------------LGS-DAQ--
	Mobilicoccus pelagius	497128022	C-E--I-----S-K-LAGI		SDHLR-G--D---VP--LAS-D----
	Mycobacterium tuberculosis	657343554	-Q--VG-QE---DELAAI		SPELT-------TI-------DCR--
	Promicromonospora sukumoe	518860713	C-A--I-----S---LAKI		SEHLT-E--S---V---LAS-D-V--
	Ruania albidiflava	551299390	C-EN-I-------DQLADI		DPALT-------TLG--LAS-D-V--
	Streptacidiphilus jeojiense	663211729	C-P--L--S-----Q-AEI		SPTLT-E-----TVH-AIAS-D-R--
	Tetrasphaera australiensis	586946012	C-E--I-----S-N-LAAI		SPHLTSG------VQ--LAS-DA---
	Thermobifida fusca	499611740	C-E--ID-P--S-A-LAAI		SPHLT-------TVQ--LES-AAR--
	Varibaculum cambriense	551244499	C-EN-I--A--S--Q-AAI		NPLLT-E------PA---R--A-R--

Supplementary figure S23

Partial sequence alignment of argininosuccinate lyase showing a 5 aa insertion that is specific for *Bifidobacteriales*. Although not shown, this CSI is present in all *Bifidobacterium* species and subspecies.

Group	Species	Accession	30		79
Bifidobacteriales	Bifidobacterium adolescentis	500063173	RRYKLNVEKIATGDVNKIAEVVRDLAQRDVD	E	HGLSAGEKRMLTKARSIL
	Bifidobacterium angulatum	489923422	----------------N--------------	-	------------------
	Bifidobacterium animalis	490328283	----------------N--------S-----	-	------------------
	Bifidobacterium asteroides	504834862	-------------E-----------S-----	-	-----------I---K--
	Bifidobacterium boum	651390685	----------S-----Q--------S--N--	-	------------R-----
	Bifidobacterium breve	489925569	-------------------------------	-	---------------A--
	Bifidobacterium catenulatum	489931914	-------------------------------	-	---------------N--
	Bifidobacterium choerinum	639202864	----------S--------------------	-	------------------
	Bifidobacterium dentium	489937269	-------------------------------	-	------------------
	Bifidobacterium gallicum	493338888	---------L---------------------	-	----------------V-
	Bifidobacterium longum	547239616	-------------------------------	-	---------------A--
	Bifidobacterium magnum	551238797	-------------------------------	-	------------------
	Bifidobacterium minimum	551240989	-------------------------S-----	-	------------R-----
	Bifidobacterium moukalabense	575769893	-------------------------------	-	------------------
	Bifidobacterium pseudolongum	551238161	----------S--------------------	-	------------------
	Bifidobacterium ruminantium	651886160	-------------------------S-----	-	------------------
	Bifidobacterium subtile	639061570	-------------------------------	-	------------R--N--
	Bifidobacterium thermacidophilum	657871748	----------S-----Q--------S--N--	-	------------R-----
	Bifidobacterium thermophilum	505263294	----------S-----Q--------S--N--	-	------------R-----
	Bifidobacterium tsurumiense	651882348	-------------------------------	-	------------R-----
	Bifidobacterium actinocoloniiforme	705418837	-------------E-----------S-----	-	-----------G------
	Bifidobacterium aesculapii	943596543	-------------------------------	-	------------------
	Bifidobacterium biavatii	705397546	-------------------------------	D	------------------
	Bifidobacterium bifidum	489909292	---------L---------------------	-	------------R-----
	Bifidobacterium bohemicum	705454160	-------------------------S-----	-	------------RS----
	Bifidobacterium bombi	763213821	-------------E-----K-----S--N--	-	------------RS-G--
	Bifidobacterium callitrichos	759443355	-------------------------------	-	------------------
	Bifidobacterium coryneforme	799123451	-------------E-----------S-----	-	-----------I---K--
	Bifidobacterium crudilactis	736120936	-------------------------------	-	------------------
	Bifidobacterium cuniculi	705443925	-------------------------------	-	------------------
	Bifidobacterium gallinarum	705424794	-------------------------------	-	------------R-----
	Bifidobacterium kashiwanohense	705413663	-------------------------------	-	------------------
	Bifidobacterium merycicum	705456668	----------------N--------------	-	------------------
	Bifidobacterium mongoliense	705434995	-------------------------------	-	------------R-----
	Bifidobacterium pseudocatenulatum	490332154	-------------------------------	-	------------------
	Bifidobacterium psychraerophilum	705398566	-------------------------------	-	------------------
	Bifidobacterium pullorum	705441073	-------------------------------	-	------------R-----
	Bifidobacterium reuteri	763216061	-------------------------------	-	---------------A--
	Bifidobacterium saguini	727803050	-------------------------------	-	---------------A--
	Bifidobacterium stellenboschense	736510399	-------------------------------	-	------------------
	Bifidobacterium indicum	655535347	-------------E-----------S-----	-	-----------I---K--
	Bifidobacterium saeculare	672992517	-------------------------------	-	------------R-----
	Bifidobacterium scardovii	673000025	-------------------------------	-	------------------
	Bifidobacterium stercoris	673000907	-------------------------S-----	-	------------------
	Gardnerella vaginalis	518090738	-L--------------N--------S-----	-	-----------SR-----
	Alloscardovia criceti	516878123	------Q----S-----V-----------DN	D	-----------SR--T--
	Scardovia inopinata	493335966	-------------------------S---D-	D	-----------S---G--
	Scardovia wiggsiae	494249628	-------------------------S---DE	D	-----------AR--GV-
	Parascardovia denticolens	493331939	-------------------------S---D-	-	-----------S---N--
	Alloscardovia omnicolens	545376333	------Q----S-----V-----------DN	D	-----------SR--T--
Other Bacteria 0/500	Corynebacterium callunae	505465202	----A-Q-RL-S-----V-------WR--Q-		R----------S---QV-
	Gulosibacter molinativorax	652541951	----S-M--L-S---I-VS------WR--Q-		K----------S---QV-
	Herbidospora cretacea	663667715	----A-L--L-S-----V-------WR--RE		R----------A---Q--
	Humibacter albus	551280649	----A-L--L-S---I-VS------WR--Q-		R----------A---Q--
	Isoptericola variabilis	503605646	----A-L--L-S---I-V-------SR--A-		R----------A---Q--
	Kocuria rhizophila	518140287	----A-L--L-S---L-V-------WR--RG		K--------------Q--
	Kutzneria albida	644672990	----A-L--L-S-----V-------WR-EK-		R----------A---Q--
	Mobiluncus curtisii	490106909	----A----------V-V-------SR--SN		R--------------Q--
	Mycobacterium tusciae	493286058	----A-L--L-S-----V-------WR--QE		R----------A---Q--
	Nonomuraea coxensis	522033449	----A-L--L-S-----V-------WR--RE		R----------A---Q--
	Oerskovia turbata	663098921	----A-L--L-S---I-V-------SR--A-		R----------A---Q--
	Pseudonocardia dioxanivorans	503443434	----A-L--L-S-----V-------WR-EK-		R----------A---Q--
	Saxeibacter lacteus	656112760	----A-L--L-S-----V-------WR--RE		R----------A---Q--
	Spirillospora albida	663134254	----A-L--L-S-----V-------WR--KE		R----------A---Q--

Supplementary figure S24

Partial sequence alignment of CarD family transcriptional regulator showing a 1 aa insertion that is specific for *Bifidobacteriales*. Although not shown, this CSI is present in all *Bifidobacterium* species and subspecies.

Group	Species	Accession	112				152
Bifidobacteriales	Bifidobacterium adolescentis	547074268	GYAPMAVALLADWA	M	FDPTG	PR	LHRMEIDILPENKRSRAVA
	Bifidobacterium angulatum	489924264	-I----L-M-----	I	GS-D-	-Q	---L--A-----G--L--V
	Bifidobacterium animalis	490389075	-LT-L---M-----	L	L----	-Q	---L----V---E---R-V
	Bifidobacterium asteroides	504835293	-F--L-L-M--N--	F	R-RR-	-H	---L-VA-----Q--LR-V
	Bifidobacterium bifidum	489911033	-F------------	F	C----	--	------AL----E---R--
	Bifidobacterium boum	651390588	-I--L---V-----	F	DAAD-	-K	---I--A---Q-R---R-P
	Bifidobacterium breve	489926505	-F------------	L	T--F-	-V	---L--A---D-A--L--V
	Bifidobacterium catenulatum	489932737	--------------	-	---S-	-Q	-------L----A---K--
	Bifidobacterium choerinum	639202590	-FT-L--TM-----	-	L--D-	--	---V---LV-Q-E---R-V
	Bifidobacterium dentium	489934105	-F-----T------	-	---D-	-H	---L--AL----A---R--
	Bifidobacterium gallicum	493338352	-F--L--TM-----	-	-A-V-	-H	---LG-----S-H---R-V
	Bifidobacterium longum	494112228	-F----L-M-----	L	G--F-	-A	---L--A---D-A--L--V
	Bifidobacterium magnum	551239642	-LT-L--CIV----	L	-YE--	-L	---L----V-S-Q--QS--
	Bifidobacterium moukalabense	575769361	-FT-----------	-	-N-D-	-H	---L--AL----V---R-
	Bifidobacterium pseudocatenulatum	490330636	--------------	-	---S-	-Q	-------L----E---K--
	Bifidobacterium pseudolongum	551238480	-LT-L---MVC---	L	QM---	--	-------LV---E-----V
	Bifidobacterium ruminantium	651886880	N--------M----	-	--A--	--	-------M----E---N--
	Bifidobacterium subtile	639065341	I--T---------	L	H--D-	-G	---L--AM--H-A---R--
	Bifidobacterium thermacidophilum	657871224	-I--L---I-----	F	DC-D-	--	---I--A-I-D-H---R-T
	Bifidobacterium thermophilum	505263892	-I--L---IV----	F	DC-D-	--	---I--A-I-D-H---R--
	Bifidobacterium tsurumiense	651881832	-IM-L---M-----	L	GESS-	-K	---L---M-IN-R--HR--
	Bifidobacterium actinocoloniiforme	914249305	-F--L-L---G---	L	RS-D-	--	-----VA-----A--LR-VA
	Bifidobacterium biavatii	705395957	----L---M-----	F	Y--S-	--	---L--AL----A---R-
	Bifidobacterium bohemicum	672949121	-I------------	-	L-AA-	S-	---L--AM----RC-KR--
	Bifidobacterium callitrichos	759442551	-I--T-L-M-----	L	A----	--	---L--A-----E--L--
	Bifidobacterium coryneforme	799124011	-F--LSL-------	F	RAD--	-H	---I-VA-----Q--L---
	Bifidobacterium crudilactis	736121785	-FT-L---MAS---	-	L--E-	-H	---I--A-----R--KR-
	Bifidobacterium cuniculi	705443002	-LT-L---M-----	L	H--H-	--	---L--------Q---R-
	Bifidobacterium gallinarum	917310429	-F--T---V-----	L	T-AA-	--	---L--AV----E--L---
	Bifidobacterium indicum	705389097	-F--LSL-------	F	RAD--	-H	---I-VA-----Q--L---
	Bifidobacterium kashiwanohense	757807658	--------------	-	---S-	-Q	-------L----E---K--
	Bifidobacterium merycicum	705459745	-I----L-M-----	I	GS-D-	-Q	---L--A-----Q--L--
	Bifidobacterium mongoliense	917311320	-I------------	L	L-EA-	--	---I--A-----R--LR--
	Bifidobacterium psychraerophilum	705397381	-F--L---MAS---	L	T--S-	--	---I--A-----R--KR-
	Bifidobacterium pullorum	705444548	----A--GV-----	L	T----	--	---L--AV----E--L---
	Bifidobacterium reuteri	672993277	-F----L-M-----	L	T--F-	-A	---L--A---D-A--L--
	Bifidobacterium saeculare	672993589	-F--T---V-----	L	A-AA-	--	---L--AV----E--L---
	Bifidobacterium saguini	727803275	-F----L-M-----	L	T--F-	-A	---L--A-----A--L--
	Bifidobacterium scardovii	705446003	-F--L---------	F	A--A-	-Q	---L--A-----H---R-
	Bifidobacterium stellenboschense	673003563	-I--T-L-M-----	I	G----	--	---L--A-----T--L--
	Bifidobacterium stercoris	673001408	--------------	-	-----	--	--------------F----
	Parascardovia denticolens	493331631	--I-Q---I-----	F	K--Q-	--	---L--NV----A--IR--
	Scardovia inopinata	493335676	--I-R-A-Q-C---	F	--E--	--	---I---MI-D-F--VR--
	Scardovia wiggsiae	515714918	--I-R-A-V-S---	F	---A-	--	---L---MI---T--VR--
Other Bacteria 0/500	Janibacter hoylei	750599210	-I--T-------HL		M--Q-	RA	---VQLE-R-D-AP-L---
	Acidothermus cellulolyticus	500038291	-IM-T-L--VV-HC		-G-G-		---I-AN-R---AA--R-V
	Agromyces subbeticus	551274144	-IT-T----AT-YC		-GVLR		------C-R---AP-LR-V
	Arthrobacter castelli	652423348	-V--T---MAT-HC		INSL-		---I-VN-R---TA-LR-V
	Beutenbergia cavernae	502173778	-IT-L---MAS-YC		WFVL-		---I-VN-R---AA-LR-V
	Cryptosporangium arvum	589395134	-VI-T-L-MAV-FS		-AHAA		---I-VN-R---TS--R-V
	Granulicoccus phenolivorans	652531727	-II-T---MAT-YC		-AVL-		-----VA-R---EK-LR-V
	Isoptericola variabilis	503605553	-VI-TS--MAT-YC		-TAL-		---I--N-R---GP-LR-V
	Jiangella gansuensis	652480141	-IM-T---MAV-YC		-FVA-		---L-VN-R---RA-LR-V
	Longispora albida	517159089	-VT-T-L--AV-H-		-G-G-		---V-VN-R----P--R-V
	Mobiluncus curtisii	490107261	-LT-L-L-MVI--G		-ASY-		---L-VN-R---RP-LR-V
	Nonomuraea coxensis	648523037	-IT-T-L-MAV-HC		-FT--		---L-AN-R---SA--R-V
	Streptosporangium roseum	502658873	-IT-T---MAV-HC		-FT--		---V-AN-R---HA--R-V
	Thermobifida fusca	499610090	-VM-T----AV-H-		-FTV-		---V-AN-R---HK--R-V
	Verrucosispora maris	503496785	-VI-T-L--VV-H-		-G-G-		---V-VN-R---RP--R-V
	Xylanimonas cellulosilytica	502643162	-VI-TG--MAT-YC		-QVL-		---I-VN-R---E--LR-V
	Yaniella halotolerans	657928257	NIV-T----A--YL		LSHV-		---I--N-V---AP-IR-V

Supplementary figure S25

Partial sequence alignment of acetyltransferase GNAT family showing a 1 aa insert and a 2 aa insert that are either completely or largely specific for the order *Bifidobacteriales.* Although not shown, this CSI is present in all *Bifidobacterium* species and subspecies.

Group	Species	ID	70		110
	Bifidobacterium adolescentis	489904236	LNPAQQVVKIVNEELTAVLG	A	GVDRPLNFAKNPPTIIMLAG
	Bifidobacterium angulatum	489924097	----------------S---	-	--------------------
	Bifidobacterium animalis	490388912	--------R---D---DI--	Q	--------------------
	Bifidobacterium asteroides	504834263	--------S--YD---SI--	-	--------------V-----
	Bifidobacterium bifidum	489908452	----------------D---	Q	--------------------
	Bifidobacterium boum	651391566	-----------------I--	-	--------------------
	Bifidobacterium breve	489924903	----------------D---	Q	--------------------
	Bifidobacterium catenulatum	489932891	--------------------	-	--------------------
	Bifidobacterium choerinum	639203212	------------G---DI--	Q	----------T---------
	Bifidobacterium dentium	489934256	----------------D---	-	--------------------
	Bifidobacterium gallicum	493337956	------------D---DI--	Q	-----I----Q---------
	Bifidobacterium longum	494114030	----------------D---	Q	--------------------
	Bifidobacterium magnum	551239548	----------------DI--	R	--E-------TG--------
	Bifidobacterium minimum	551240882	--------R-------TI--	-	-------V------V-----
	Bifidobacterium moukalabense	575769281	----------------G---	-	--------------------
	Bifidobacterium pseudocatenulatum	490330760	--------------------	-	--------------V-----
	Bifidobacterium pseudolongum	651884037	------------D---DI--	Q	--------------------
	Bifidobacterium ruminantium	651887348	--------------------	-	--------------------
	Bifidobacterium subtile	639065606	--------Q--------I--	-	--------------------
	Bifidobacterium thermacidophil	657872295	-----------------I--	-	--------------------
	Bifidobacterium thermophilum	505262730	-----------------I--	-	--------------------
	Bifidobacterium tsurumiense	651883094	----------------SI--	Q	--------------V-----
	Bifidobacterium actinocoloniiforme	705421831	--------S---D---QI--	-	----------T---V-----
Bifidobacteriales	Bifidobacterium aesculapii	943597222	-----------------I--	-	--------------------
	Bifidobacterium biavatii	705397144	------------D---DI--	-	--------------------
	Bifidobacterium bohemicum	705456086	----------------GI--	-	--------------------
	Bifidobacterium bombi	763215523	----------------NI--	-	--------------------
	Bifidobacterium callitrichos	759444924	-----------D----D---	-	--------------------
	Bifidobacterium coryneforme	799122688	--------S--Y----GI--	S	--------------V-----
	Bifidobacterium crudilactis	736121531	------I-S-------K---	S	----------K---------
	Bifidobacterium cuniculi	705449746	------------D---GI--	Q	--------------------
	Bifidobacterium indicum	705391157	--------S--Y----GI--	S	--------------V-----
	Bifidobacterium kashiwanohense	746131487	--------------------	-	--------------------
	Bifidobacterium merycicum	705459918	---V------------D---	-	--------------------
	Bifidobacterium mongoliense DSM 21395	672983031	--------R---D----I--	-	------A-------V-----
	Bifidobacterium psychraerophilum	705403353	------I-S-------K---	-	----------K---------
	Bifidobacterium pullorum	705441691	-----------YD---G---	Q	--------------------
	Bifidobacterium reuteri	763217138	-----------D----D---	Q	--------------------
	Bifidobacterium saguini	727804562	----------------D---	Q	--------------------
	Bifidobacterium scardovii	705450083	----------------D---	-	--------------------
	Bifidobacterium stellenboschense	672999176	-----------------I--	-	--------------------
	Bifidobacterium saeculare	672993503	-----------YD---G---	Q	--------------------
	Bifidobacterium stercoris	673001326	--------------------	-	--------------------
	Parascardovia denticolens	493332616	--------S---D---QI--	-	--------------------
	Scardovia inopinata	493336691	--------S---D---DI--	-	--------------------
	Scardovia wiggsiae	494251809	--------S---D---EI--	-	N-------------------
	Metascardovia criceti	516876369	--------S--HD---SI--	-	--------------V-----
	Alloscardovia omnicolens	545371376	--------S--H--------	-	--------------------
	Gardnerella vaginalis	490206952	------------D---Q---	-	----Q-----Y---V-----
	Gulosibacter molinativorax	652543421	--------Q------VRI--	G	DEA-Q-S---R---V---V-
	Pseudoclavibacter faecalis	518449077	--------Q------VRI--	G	-EA-K-Q---R---V---V-
	Brachybacterium faecium	502489787	------------D--VE---		-ATGE-H-------V-----
	Corynebacterium ulcerans	503677723	----E--I--------GI--		-ET-R--L------V-----
	Agrococcus lahaulensis	651233264	------------D--I-I--		-DS-R-EL--T---V-----
	Gordonia rhizosphera	493375018	------------D--VGI--		-ET-RVT---K---V-----
	Intrasporangium calvum	503258459	---------------VTI--		-ET-QIR-------V-----
	Jiangella gansuensis	652480863	-------I----D--VRI--		-ET-R-R---Q---V-----
	Leucobacter salsicius	516404559	--------Q------VEI--		-AQ-R-E-------------
Other Bacteria 2>500	Longispora albida	517162171	-------I---H---IS---		-EA-R-Q---H---V-----
	Micromonospora aurantiaca	503049604	------I--------INI--		-EG-R-Q---H---V-----
	Nocardiopsis chromatogenes	516222295	-------I-------IE---		-ET-EIR-------V-----
	Nonomuraea coxensis	522035330	------------D--IEI--		-ET-R-RL--T---V-----
	Paraoerskovia marina	656320545	------------D--VGI--		-ES-S--L--T---V-----
	Rhodococcus equi	503181913	---------------VGI--		-ET-R-Q---T---V-----
	Segniliparus rugosus	494735332	-----------D----EI--		-ES-R-Q---T---V---V-
	Tetrasphaera elongata	499073064	------------D--VSI--		-ET-K-RL------V-----
	Thermobispora bispora	502896258	-----------HD--IE---		-ET-RIR---T---V-----
	Turicella otitidis	490737759	-------I----Q---GI--		-ET-R--L------V-----

Supplementary figure S26

Partial sequence alignment of signal recognition particle protein showing a 1aa insertion that is largely specific for the order *Bifidobacteriales*. In addition to the *Bifidobacteriales,* this CSI is also present in *Pseudoclavibacter faecalis* and *Gulosibacter molinativorax*. Although not shown, this CSI is present in all *Bifidobacterium* species and subspecies.

Group	Species	ID	51		90
Bifidobacteriales	Bifidobacterium angulatum	489923970	GNHVIVINAAKVALTGNKLG		KELYKHSGRPGGLRRDTYAQ
	Bifidobacterium animalis	490389100	--Y--I---D-I----D-MD		----S-----------S--E
	Bifidobacterium asteroides	504835260	--N------S-MV-----SD		-V--T-----------S-G-
	Bifidobacterium bifidum	503155567	-----I---D-I------M-		----S-----------S--E
	Bifidobacterium boum	651390575	---------S-I--------		----S-----------K--D
	Bifidobacterium breve	489926530	-----I---D-I------M-		----S-----------S--E
	Bifidobacterium choerinum	639202576	--Y--I---D-I----D-MD		----S-----------S--E
	Bifidobacterium dentium	489934077	-----I---D-I------M-		----------------S--E
	Bifidobacterium gallicum	493338328	--F--I---D-I-F----M-		----A-----------S---
	Bifidobacterium longum	501574073	-----I---D-I------M-		----S-----------S--E
	Bifidobacterium magnum	551239668	-----I---D-I--S---M-		----S-----------S--E
	Bifidobacterium minimum	551240482	---------G----------		----S--------------K
	Bifidobacterium moukalabense	575769376	-----I---D-I------M-		----M-----------S--E
	Bifidobacterium pseudolongum	551237766	--Y--I---D-I----D-MD		----S-----------S--E
	Bifidobacterium ruminantium	651886868	-----IL--D-I-------S		----M-----------S---
	Bifidobacterium subtile	639065387	-----IV----I--------		----M---------S-N-GD
	Bifidobacterium thermacidophilum	657871239	-------------------D		-V--S-----------K-VD
	Bifidobacterium thermophilum	505263876	-------------------D		-V--S-----------K--D
	Bifidobacterium tsurumiense	651881818	-----I---E-I--------		----S-----------S--K
	Bifidobacterium actinocoloniiforme	705420984	---------S--------D-		-V--A-----------S-GE
	Bifidobacterium adolescentis	489904498	-----IL--D-I--------		----M-----------S---
	Bifidobacterium aesculapii	943597257	--F------D-I----D---		----A-------------G-
	Bifidobacterium biavatii	705395729	-----I---D-I------M-		----S-----------S--E
	Bifidobacterium bohemicum	705455072	-D---I---E-I--S-K-MD		-V--S---------E-S--D
	Bifidobacterium bombi	763215276	-A-------E-I-------S		-V--S---------E-S--
	Bifidobacterium callitrichos	759442501	--Y------D-I------M-		----A---------------
	Bifidobacterium crudilactis	736119841	------V--S------D---		----T---------A-S-
	Bifidobacterium cuniculi	705445881	--Y------D-I----D-MD		----S-----------S--E
	Bifidobacterium merycicum	705458222	--------------------		--------------------
	Bifidobacterium mongoliense	705435866	-----I-----I------T-		-L--T---------G-S--
	Bifidobacterium psychraerophilum	705397439	---------G-I----D---		----T---------A-S-
	Bifidobacterium pullorum	705443373	---------S-I------M-		----S-----------S---
	Bifidobacterium reuteri	763216654	-DF------D-I------M-		----V-----------S--E
	Bifidobacterium saguini	727803255	-DY------D-I------M-		----S-----------S--E
	Bifidobacterium stellenboschense	736511156	--Y------D-I--------		----S-----------K-SE
	Bifidobacterium catenulatum	212660327	-----IL--D-I--------		----M-----------S-
	Bifidobacterium coryneforme	671341424	--N-------------D-S-		-V--T-----------S-GE
	Bifidobacterium gallinarum	672964893	---------S-I------M-		----S-----------S--E
	Bifidobacterium indicum	655535701	--N-------------D-S-		-V--T-----------S-GE
	Bifidobacterium pseudocatenulatum	225158513	-----IL--D-I--------		----M-----------S-
	Bifidobacterium scardovii	673000766	-----I---D-I------M-		----S-----------S---
	Gardnerella vaginalis	490207114	-----I---E-I-------S		----S-----------S--E
	Alloscardovia criceti	648490168	--F------D-I--S----A		-NI-H---F-S---A-S--E
	Parascardovia denticolens	493331516	------L--D-IS-SR---D		-N--H---F-S---A-S--E
	Scardovia inopinata	493335597	--Y------E-IS-S----E		-N--H---Y-S---A-S---
	Scardovia wiggsiae	494250679	--F------E-IS-S-G--D		-N--H---F-S---A-S--E
	Alloscardovia omnicolens	545376704	--F------D-IS-S----N		-N--S---Y-S---A-S--E
Other Bacteria 1>500	Acaricomes phytoseiuli	516859451	-DF--I---E------A--E	Q	-RA-R---Y-----SVN--E
	Arthrobacter arilaitensis	503114940	-D---I---D---------E	N	-RA-R---F----KSIN--D
	Beutenbergia cavernae	506363871	-DF---V--D--------EQ	Q	-LA-R---Y----KATS--E
	Cellulomonas massiliensis	517966155	-DF---V--E------T-RD	T	-LA-R---Y-----ATP-SE
	Glaciibacter superstes	551265726	-DF--IV--E-I----A--D	Q	-KA-R---Y----TAT---E
	Haloglycomyces albus	644028461	-DY--IV--E-AV--R---R	D	-KA-R---Y----KEQS--E
	Kocuria atrinae	515564233	-DF--I---E------A--E	Q	-RA-R---Y----KSTN--E
	Leifsonia aquatica	545656480	-DF--I---D------Q-AE	Q	-KA-R---Y----KAT--SE
	Mycobacterium vanbaalenii	500102720	-DF------D-I--S-D--N	K	-FA-R---Y-----KRSIGE
	Nocardiopsis alba	504721408	-DF--IV--E---------E	Q	-RA-R---Y-----SVA--D
	Nodularia spumigena	493211983	-DF------E-I-V--K-RT	Q	-LYRR-------MKTE-F-K
	Pseudoclavibacter faecalis	518448820	-DF-VIV--D------A--E	Q	-KA-R---Y-----AES-TE
	Saccharomonospora azurea	491585774	-DF--IV--D--------RD	Q	-FA-R---Y----SKRSFGE
	Saccharothrix espanaensis	504918111	-DF--I---D------K-RD	Q	EFV-R---H-----QRSFGE
	Sanguibacter keddieii	502631298	-DF------D--------RE	Q	-LA-T---Y-----AVA-SD
	Smaragdicoccus niigatensis	516914149	-DY--I---D---------A	D	-KI-R---H----KSR-AGE

Supplementary figure S27

Partial sequence alignment of 50S ribosomal protein L13 showing a 1 aa deletion that is specific for the order *Bifidobacteriales*, except for the presence of a similar insert in *Dethiosulfovibrio peptidovorans.* Although not shown, this CSI is present in all *Bifidobacterium* species and subspecies.

Group	Species	ID	637		686
Bifidobacteriales	Bifidobacterium adolescentis	547082727	RILLLTLFYRYMRPLIEYGHVYAAVPPLHRIALT		GTHKGEYIYTYSDDEL
	Bifidobacterium angulatum	489922697	-----------------N-Y--------------		-S----F---------
	Bifidobacterium animalis	549638555	-T-------------V-A---------------A		-AR-------------
	Bifidobacterium asteroides	504834565	-----------------N-R-------------A		-RN--KF---------
	Bifidobacterium bifidum	489910357	-----------------H-Y-------------A		-S----F---------
	Bifidobacterium boum	651390766	---------------V-A---------------A		-K--------------
	Bifidobacterium breve	489927323	-----------------H-Y--------------		-S--------------
	Bifidobacterium catenulatum	489931135	-----------------H----------------		-AR-------------
	Bifidobacterium choerinum	639202119	-T---------------A----------------		-PR-------------
	Bifidobacterium dentium	489936346	-----------------H----------------		-A--------------
	Bifidobacterium gallicum	493336890	-T-------------VDA---------------N		ASR--Q----------
	Bifidobacterium longum	494111880	-----------------H-Y--------------		-S--------------
	Bifidobacterium magnum	551238698	-T---------------E----------------		-PR-------------
	Bifidobacterium minimum	551240617	-------------G---D-R-------------S		-KR---FL--------
	Bifidobacterium moukalabense	575770667	-----------------H----------------		-S----F---------
	Bifidobacterium pseudocatenulatum	490330988	---------C------------------------		-A--------------
	Bifidobacterium pseudolongum	651884252	-T---------------E----------------		-PR-------------
	Bifidobacterium ruminantium	651886454	----------------------------------		-V----F---------
	Bifidobacterium subtile	639062798	-T---------------F---------------A		-K--------------
	Bifidobacterium thermacidophilum	657871895	-----------------A---------------A		-K----F---------
	Bifidobacterium thermophilum	505263162	-----------------A---------------A		-K----F---------
	Bifidobacterium tsurumiense	651882850	-----------------N-R-------------A		-KR-------------
	Bifidobacterium actinocoloniiforme	917298573	-----------------E-------------S-A		-KR----L--------
	Bifidobacterium aesculapii	943596061	-----------------H-Y-------------A		-SR-------------
	Bifidobacterium biavatii	705392547	-----------------H---------------A		-S--------------
	Bifidobacterium bohemicum	705455381	----------F-----SH---------------A		-SR---F---------
	Bifidobacterium bombi	763214415	----------FL--M-----I-------------		-KR---F---------
	Bifidobacterium callitrichos	759441799	-----------------H-Y-------------A		-S--------------
	Bifidobacterium coryneforme	799123106	---------------------------------A		-KR---F---------
	Bifidobacterium crudilactis	736121077	---------------V-H---------------A		-KR-------------
	Bifidobacterium cuniculi	705444420	-T---------------H----------------		-PR-------------
	Bifidobacterium gallinarum	705424517	-----------------H-R-------------A		-S--------------
	Bifidobacterium indicum	705387677	---------------------------------A		-KR---F---------
	Bifidobacterium kashiwanohense	672971796	-----------------H----------------		-A--------------
	Bifidobacterium merycicum	705459002	-----------------H-Y--------------		-S----F---------
	Bifidobacterium mongoliense	705435152	-------I-------------------------A		-S--------------
	Bifidobacterium psychraerophilum	705401273	-N----F-----MVTA-Y--V-M---Y-LKW-		--AHDFV-T-A-
	Bifidobacterium pullorum	759446750	-----------------H-R-------------A		-S--------------
	Bifidobacterium reuteri	763216401	-----------------H-Y--------------		-SR----L--------
	Bifidobacterium saeculare	705430447	-----------------H-R-------------A		-S--------------
	Bifidobacterium saguini	727802741	-----------------H-Y--------------		-S--------------
	Bifidobacterium scardovii	705448677	-----------------H---------------A		-S----V---------
	Bifidobacterium stellenboschense	736514454	-----------------H---------------A		-S--------------
	Bifidobacterium stercoris	673002689	----------------------------------		----------------
	Alloscardovia omnicolens	545375984	-T--------------AE----------------		-KR---------EE--
	Parascardovia denticolens	493332162	-T-------------V-A---------------A		-K----FV----EE--
	Scardovia inopinata	493336206	-T-------------VQA---------------M		-K-----V----EE--
	Scardovia wiggsiae	494248940	-T-------------V-S---------------A		-KR----L----E---
	Alloscardovia criceti	516878177	-T--------------AE---------------A		-KR---------EE--
	Gardnerella vaginalis	490208377	-----------------H---------------A		-KR-------------
Other Bacteria 1>500	Brachybacterium muris	516434628	-T------H------V-A-R----------VEIV	HG	-AK-N-FV----EA--
	Cellulomonas flavigena	502882308	-T------F------V-D-R-----------EVI	GA	-SR-N--L----EA--
	Demetria terragena	516900127	-T------F-----MV-A-R-F---------ETS	AA	-RK---V-----E--M
	Intrasporangium oryzae	586965919	-T------F------V-A-R----M------EVI	NA	-SK-N------NEL-M
	Jonesia quinghaiensis	656030607	-T------F-------DE-R----------VEVI	GA	-SR-NH------EA--
	Microtetraspora glauca	663718479	-T------Q-----MV-A-R-F---------E-V	QP	KKGQDK-V-----S--
	Mobilicoccus pelagius	497132776	-T------F------VAA-R-----------EVL	GA	-SR---LV---NEN-M
	Oerskovia turbata	663111437	-T------F------VDA-R----------VEVI	GA	-SR-N-------EA--
	Paraoerskovia marina	656321574	-T------F-----M-DN-R-----------EVV	GS	-RR---------EA--
	Sanguibacter keddieii	502629961	-T------F------VDS-R----------VEVI	GA	-SR-N-------EA--
	Trueperella pyogenes	644254437	-T------F------V-A-R----------VEVA	GR	-GK---F--S---A--
	Streptomyces viridochromogenes	490091530	-T------H-----MV-A-R-F---------EIV	QP	KKGQDK-V-----R--
	Xylanimonas cellulosilytica	502642334	-T-------------V-A-R-F---------EVY	GA	RGKGN-------EA--
	Arcanobacterium haemolyticum	502935017	-T------F------V-A-R-----------EVA	GQ	-RR----------TQ-

Supplementary figure S28

Partial sequence alignment of DNA gyrase B subunit protein showing a 2 aa deletion that is specific for the order *Bifidobacteriales*. Additionally, a similar CSI is also present in *Actinobaculum* sp. oral taxon 183. Although not shown, this CSI is present in all *Bifidobacterium* species and subspecies.

Group	Species	ID	171 … 216
			LIAGTYTPVAFALQPFWRNTIIIS MWACTAIAIIINVIWTTAPRWL
Bifidobacteriales	Bifidobacterium angulatum	489923478	LIAGTYTPVAFALQPFWRNTIIIS MWACTAIAIIINVIWTTAPRWL
	Bifidobacterium animalis	490328268	---------S---DD---KA---G --S--L--L--H-V-IN-----
	Bifidobacterium asteroides	504834878	--------IS---D----RI--LG --GASLV-M-VH-F-IE-----
	Bifidobacterium bifidum	489909259	---------S---A-HM--A--AG I-S--LV-L--H---IS-----
	Bifidobacterium boum	651390698	---------S---E-RM-DL---- --V----VM--H---IN-----
	Bifidobacterium breve	489924606	I----N---L---N-AI-RPYLTV I--TA--GT-LHIV-LRT-N-V
	Bifidobacterium catenulatum	489931951	---------S---E-----S--AG --I--TV-L--H---IS-----
	Bifidobacterium choerinum	639202850	---------S---D---------G --S---V--L-H-V-ID-----
	Bifidobacterium dentium	489937294	---------S---SS---DS---G --T--IV-LV-H---IS-----
	Bifidobacterium gallicum	493338861	---------S---DD--------G --S--F--LV-H---I------
	Bifidobacterium longum	494111115	---------S---E-----V---- ---------V-H---IN-----
	Bifidobacterium magnum	551238785	--------I----DT----A---G --S--FV-LL-H---I------
	Bifidobacterium minimum	651887901	-------------PRP--EG--TG --S--LV-L--H---I------
	Bifidobacterium moukalabense	575769878	---------S---S----DS---G --T--IV-LV-H---IS-----
	Bifidobacterium pseudolongum	651883491	--------A----DA--------G --S--LV-L--H-V-IN-----
	Bifidobacterium ruminantium	651886146	---------S---T-----S--AG --T--T--L--H---IS-----
	Bifidobacterium subtile	639061523	---------S---DS---TA--VG --S--FV-MV-H---IS-----
	Bifidobacterium thermacidophilum	657871761	---------S-----RM-DI--V- --V----VM--H---IN-----
	Bifidobacterium thermophilum	505263275	---------S-----RM-DL--V- --V----VM--H---IN-----
	Bifidobacterium tsurumiense	651882361	--------T---------V---G --S---A-M-VH---IS-----
	Bifidobacterium actinocoloniiforme	672944127	-------------DG---RV-LVG I-SA-IVVMF-H-V-IS-----
	Bifidobacterium adolescentis	748204296	---------S---T----DS--AG --I--TV-L--H---IS-----
	Bifidobacterium aesculapii	943596561	---------S---------F---- --V---V-M--H---I------
	Bifidobacterium biavatii	705397574	---------S---T-H--DA--AG -----LV-LV-H---IG-----
	Bifidobacterium bohemicum	763226099	---------S---SVK--TI--SG --V--TA-L--H---ID-----
	Bifidobacterium bombi	763213892	--------IS---SHH---L--VG --V--VA-ML-H---IN-----
	Bifidobacterium callitrichos	917512674	---------S---------F---- ------V----H---IN-----
	Bifidobacterium coryneforme	705404318	-------------DT---R----G --SA-LV-MVVH-F-IN-----
	Bifidobacterium crudilactis	736122335	--------A----SLA-QRV---G L-S---V-MLVH---IS-----
	Bifidobacterium cuniculi	917313737	--------IS---DD---QV---G --S---V-L--H-V-IN-----
	Bifidobacterium gallinarum	705424754	---------S---E-----A--GG -----SV-M-VH---IS-----
	Bifidobacterium indicum	705389614	-------------DT---R----G --SA-LV-MVVH-F-IN-----
	Bifidobacterium kashiwanohense	705413624	---------S---E-----S--AG --I--TV-L--H---IS-----
	Bifidobacterium merycicum	705456742	---------S---------LV--- --V--------HGV-IN-----
	Bifidobacterium mongoliense	705435022	---------S---S---STS--VG --S--LV-L--H---IN-----
	Bifidobacterium pseudocatenulatum	490332122	---------S---E-----S--AG --I--TV-L--H---IS-----
	Bifidobacterium psychraerophilum	917315158	--------A----NQ--QRV---G L-S---V-MLVH---I------
	Bifidobacterium pullorum	705441101	---------S---E-----A--GG -----S--M-VH-V-IS-----
	Bifidobacterium reuteri	672990689	---------S---------F---- -----LV-L--H---IN-----
	Bifidobacterium saeculare	705430602	---------S---E-----A--GG -----SV-M-VH---IG-----
	Bifidobacterium saguini	917310614	---------S---E-----F--T- --S--L--L--H---IN-----
	Bifidobacterium scardovii	757810721	-------------E-----A--AG I-S--LV-LV-H---IG-----
	Bifidobacterium stellenboschense	736510359	---------S---------F---- ------V-MV-H---IS-----
	Parascardovia denticolens	493331923	---------S---EER--VI--A- --IF----L--H---IN-----
	Scardovia inopinata	493335950	--------IS---SSGM--I--AT --IF-T--L--H---IN-----
	Scardovia wiggsiae	494249647	---------S---S-G------LT --IF-G--LL-H-V-IN-----
Other Bacteria 2>500	Brevibacterium senegalense	517989007	I-------L-VLCLEGTPR-VLLV V --TAAVLGVVFRIV--------
	Actinomadura atramentaria	518463682	I-------F-YLILDGMARALVL- V V-GGALAGVLFR-L-VG-----
	Arsenicicoccus bolidensis	518117097	F---S---L-LL-LEGRSRV-LL- L I---AIAGVLFRTL-ID-----
	Arthrobacter sanguinis	651429769	--------LSVT-L-SDKAS-LLW V I-SA-IAG-TFRLL-HH-----
	Balneola vulgaris	516845357	V-------ICTVVLEGNWRLWMLI G I-SFGL-GVFKKIF-MG----F
	Cellulomonas flavigena	502881211	I-------L-VL-L-TSTARLLLV I V-SGALLGLLARIF-LN----V
	Dietzia cinnamea	494906367	--------MTV-ALEAPAS--LL- I V--GALLG-VFRLF-LS-----
	Glycomyces tenuis	652537694	I---------ALALEGAAR-A-LL A V-VGA-AG-AFRTL-LS-----
	Humibacter albus	652546473	--------LGVLAL-PQKGA-LLT I V-GGALLG-AFH-F-I------
	Isoptericola variabilis	503605486	I-------L-VM-LDPRTA--LL- I V-GGAVVGLLAR-L-MD----V
	Kitasatospora setae	503903847	I-------FTIL-LDGA-QQVLLW L V-GGALAG-AFR-F-VG-----
	Kribbella flavida	502687636	--------LGMV-LHGSERVLLL- M V-GGALLG-LFRIF-VG-----
	Microbacterium yannicii	518011008	--------I-TLAL-PGKGALLLV V V-SGAL-G-LFR-F-IN-----
	Nocardioides insulae	655248845	----S---FSLM-LDGVAQ-VML- V A-GGAILGMLFRIF--E-----
	Promicromonospora sukumoe	518861117	I-------L-VM-L-RDQG--LLA L V-SGALLGLLARIF-LN----V
	Rathayibacter toxicus	653813876	--------L-VLAL-PAQGSLLLT L V-SGALLG-GFR-F-IA-----
	Streptomyces albidoflavus	663306923	I-------L-VL-L-TGQQKVLL- L V-GGA-VGMAFR-L-IN-----
	Thermobifida fusca	499610575	I-------FVLLVLDGTQR-AMLA L I-G-A-AGVLFTLV-LH-----
	Trueperella pyogenes	640529910	--------LSVM-L-TSTA-LVLG I V-SGAIVGTLVHIFRMN----F

Supplementary figure S29

Partial sequence alignment of hemolysin III showing a 1 aa deletion that is mainly restricted to the order *Bifidobacteriales*. The only exceptions observed are *Janibacter hoylei* and *Phycicoccus jejuensis.* Although not shown, this CSI is present in all *Bifidobacterium* species and subspecies.

Group	Species	Accession	56	Insertion	95
Bifidobacteriales	Bifidobacterium adolescentis	547071034	KTYIATLEGRISGQVCRRL	V	TTGVKLDDGWIKLDRCAILD
	Bifidobacterium angulatum	489923074	-------------T-----	-	----Q-------------I-
	Bifidobacterium animalis	490328510	---------HM--T-----	-	R---Q---------H-S-I-
	Bifidobacterium asteroides	504834703	-------S-H-G-N-----	-	-Q--E----L-R--H---I-
	Bifidobacterium bifidum	545408814	------ID-K-G-N-----	-	-Q--Q----LV---H---I-
	Bifidobacterium boum	651391080	-------Q-----N-----	-	SQ--Q------R--H---I-
	Bifidobacterium breve	489926031	-------------T-----	-	----Q---------H---I-
	Bifidobacterium catenulatum	489931440	-------------------	-	----Q---------------
	Bifidobacterium choerinum	639201993	----------M--N-----	-	R---Q---------H-S-I-
	Bifidobacterium dentium	489936813	-------D-----T-----	-	----Q---------------
	Bifidobacterium gallicum	493337636	---VV--D-HL--N-----	-	R---Q---------Q-S-I-
	Bifidobacterium longum	501573101	---V-----K---T-----	-	----Q---------H---I-
	Bifidobacterium magnum	551239040	---VV----KLN-N-----	-	R---Q---------K-V--
	Bifidobacterium minimum	651887726	------ID-H-G-N--H--	-	-Q--Q-E--H-SF-----I-
	Bifidobacterium moukalabense	575770119	-------------T-----	-	----Q---------------
	Bifidobacterium pseudolongum	551237049	---------HM--N-----	-	R---Q---------H-S-I-
	Bifidobacterium ruminantium	651886304	-------------------	-	--------------------
	Bifidobacterium subtile	639065929	---------K-G-N-----	-	SQ--Q----L-M--H---I-
	Bifidobacterium thermacidophilum	657872153	-------Q-----N-----	-	SQ--Q------R--H---I-
	Bifidobacterium thermophilum	505263467	-------Q-----N-----	-	SQ--Q------R--H---I-
	Bifidobacterium tsurumiense	651882125	-------D-K---TI----	-	-Q--Q---------H---I-
	Bifidobacterium actinocoloniiforme	705421193	---M--VR-KLG-N-----	-	-Q--L-N--L----H---V-
	Bifidobacterium aesculapii	943596755	------I------T-----	-	K---Q---------H---I-
	Bifidobacterium biavatii	705392834	-V-------KL--T-----	-	----Q---------H---I-
	Bifidobacterium bohemicum	705453717	------V----G-NI----	-	-Q--N----L-R------I-
	Bifidobacterium bombi	763214112	---------K-G-N-----	-	-Q--N----L-R--H---M-
	Bifidobacterium callitrichos	759442175	---V-----K---T-----	-	----Q---------H---I-
	Bifidobacterium crudilactis	736120703	---------K-G-N-----	-	-Q--R----Q-T--H---I-
	Bifidobacterium cuniculi	705444634	----V--Q-KM--T-----	I	R---Q---------H-S-I-
	Bifidobacterium gallinarum	705423871	-------------T-----	-	SQ--Q---------H-----
	Bifidobacterium kashiwanohense	705412022	-------------------	-	----Q---------------
	Bifidobacterium merycicum	705458668	---------K---T-----	-	----N-------------I-
	Bifidobacterium mongoliense	705436855	-----------G-N-----	-	SQ--R----L----H---I-
	Bifidobacterium pseudocatenulatum	490332743	-------------------	-	----Q---------------
	Bifidobacterium psychraerophilum	705400347	---------K-G-N-----	-	-Q--H----Q-T--H---I-
	Bifidobacterium pullorum	705442415	---------K-G-N-----	-	SQ--Q---------H-----
	Bifidobacterium reuteri	763215726	------V---L--T-----	-	K---Q---------H---I-
	Bifidobacterium saguini	727803624	------V--KL--T-----	-	K---Q---------H---I-
	Bifidobacterium stellenboschense	736512271	------V--KL--T-----	-	K---Q---------H---I-
	Bifidobacterium coryneforme	671340920	---MV--K-H-G-N-----	-	SQ--E----L-R--H---V-
	Bifidobacterium indicum	655535220	---MV--K-H-G-N-----	-	SQ--E----L-R--H---V-
	Bifidobacterium saeculare	672991779	-------------T-----	-	SQ--Q---------H-----
	Bifidobacterium scardovii	673000057	------VS-----T-----	-	----Q---------H---I-
	Bifidobacterium stercoris	673003399	-------------------	-	--------------------
	Gardnerella vaginalis	490208154	------V--K-N-M-----	-	SQ--N-E-----------I-
	Alloscardovia omnicolens	545375687	----V-V--H-G-NIA---	-	R---Q-------F-Q-TV--
	Alloscardovia criceti	516878256	----V-V--H-G-N-A---	-	R---Q---------Q-T---
	Scardovia inopinata	493336120	------V--H-G-G--H--	-	R---Q---------Q-S-I
	Scardovia wiggsiae	494249083	---------H-G-N-----	-	R--IQ---------H-S-V
	Parascardovia denticolens	493332093	------V--H-G-S--H--	-	R---Q---------Q-S-I-
Other Bacteria Indel (-) 1/250	Actinomyces viscosus	490701457	---V-IV--QVEPW-P-K-		RR-IE-E--EA-A--VTVK-
	Blastococcus saxobsidens	504189161	---L-QVS-SVPRDLA---		R--ID----PV-V-SFRVV-
	Cellulomonas fimi	503536960	---L-HV---VA-SLAG--		AR--E-E--VVTV--FQVVQ
	Corynebacterium variabile	663103705	------IR-PVQRD-GK--		RE--E-E--PV-V-SFR-V-
	Dermatophilus congolensis	654874256	---V-SVP-P-PRDLG---		RE--E-EE-LVQV-SFKLI-
	Gordonia terrae	498818381	---L---K-EVPRGIA--M		RE--D----PVTV-KFTVIE
	Intrasporangium calvum	503258003	---L--VR-PVGRD-GKQ-		RE--E-E--PVTV-SFKVV-
	Janibacter hoylei	495202290	-----EIP-PVPKDLGK--		RD--E-E--PVRV-SFKVV-
	Kineosphaera limosa	493641010	------VP-PVGRD-G-T-		RA--E-E--PA-V-SFKLV-
	Longispora albida	648520919	---L-EVV-P-PQGFKK-I		M---E-E---A-A-SFKVV-
	Microbacterium gubbeenense	656182024	-V---KVR-QVT---VAK-		-R-IE----P-AA-KARV--
	Mycobacterium vanbaalenii	500104472	-S-V--VL-SVPKGLG-K-		RE--E-E--PVQV-DF-VV-
	Nocardiopsis gilva	648433685	-----KVP-PVPRE-VKQV		RD--E-E--LV-V-SFRVV-
	Oerskovia turbata	663111381	---LVSV---VA-N-ANK-		LH-IE-E--PA-V--YRVV-
	Ornithinimicrobium pekingense	551301704	---V-QVH-VVGRD-G---		RD--E-E--PA-V-SFKLV-
	Propionibacterium thoenii	653568312	-S-L-EV--HVDDK-I---		AK-LT----PV-P-SVRLVQ
	Pseudonocardia spinosispora	655588260	---L-EVP-P-PRDLG--M		RD--Q-I--VV-V-SFKVI-
	Rhodococcus rhodnii	498787450	---L--VS-HLAKG-SKK-		RD-IE-E--PA-V-AFTL--
	Saccharomonospora glauca	491607860	---L-EV--TVARGLGK--		RA-IQ-E--PV-V-EFR-K-
	Thermobifida fusca	499610919	---L-KVP-PVPRD-I-QI		RK--E----FV-V-SFRVI-

Supplementary figure S30

Partial sequence alignment of pseudouridine synthase showing a 1 aa insertion that is specific for the order *Bifidobacteriales*. The only exception observed is *Nocardia otitidiscaviarum.* Although not shown, this CSI is present in all *Bifidobacterium* species and subspecies.

Group	Species	Accession	85	Insertion	124
Bifidobacteriales	Bifidobacterium adolescentis	500063064	VLEIDLQGARTVKRRAK	ELGL	EVLTVFIEPPSFEELKRRL
	Bifidobacterium angulatum	489922462	L---------R-RE--T	----	D-V----A-----------
	Bifidobacterium animalis	490329465	I---------R--E---	----	D-VY---A----D---K--
	Bifidobacterium asteroides	504835031	I--------HR-RQ--G	----	--FYI-LA----QD-VT--
	Bifidobacterium bifidum	489910403	I---------R--Q---	----	--VY---A----D--V---
	Bifidobacterium boum	651390368	I---------R--QQ-A	AM--	--VY---A---YD--V---
	Bifidobacterium breve	489925359	I---------R--Q--S	--D-	--VY---A--------C--
	Bifidobacterium catenulatum	489933162	I----I----S--Q--G	----	D-M----A-------E---
	Bifidobacterium choerinum	658449953	L---------R-RE---	----	-IV----A-----------
	Bifidobacterium dentium	489937531	-----I----R--E--E	----	D-M----A-----------
	Bifidobacterium gallicum	493337911	I---------R--E--Q	----	D--Y--LA--------H--
	Bifidobacterium longum	501572716	I---------R--Q--A	--D-	--VY---A-------E---
	Bifidobacterium magnum	651390051	I---------R--E--A	----	D-EY--LA-------VH--
	Bifidobacterium minimum	651887676	I---------R--EK-L	A---	Q-VY--LA----DD-AT--
	Bifidobacterium moukalabense	575769662	-----I----H--E--E	----	D-M----A-----------
	Bifidobacterium pseudocatenulatum	490330089	I----I----S--Q--G	---I	--M----A-------E---
	Bifidobacterium pseudolongum	651888316	----------R--E--R	----	D-VY--LA----D---K--
	Bifidobacterium ruminantium	651886731	----------S------	----	-------------------
	Bifidobacterium subtile	639063711	I---------R--D--G	DM--	D-VY---A---YD--V---
	Bifidobacterium thermacidophilum	657872565	I---------R--QK-S	QM--	D-VY---A---Y---V---
	Bifidobacterium thermophilum	505263587	I---------R--QE-S	QM--	D-VY---A---Y---V---
	Bifidobacterium tsurumiense	651882421	I---------R--EK-S	QM--	D-VY---A----D--V---
	Bifidobacterium actinocoloniiforme	917298597	I--------HR-RQC-E	G---	D-VY--LA----DD-IK--
	Bifidobacterium asteroides	797160369	I--------HR-RE---	----	--FY--LA----QD-VT--
	Bifidobacterium biavatii DSM 23969	672957106	I---------R--ET--	D---	---S---A----D--V---
	Bifidobacterium bohemicum	705455527	I---------R--Q--A	--N-	-IVS---A---YD--V---
	Bifidobacterium bombi	763215307	I---------R-RQ--A	----	-MVS---A---YD--VK--
	Bifidobacterium callitrichos DSM 23973	672960906	I---------R--QK-A	----	--M----A----D--V---
	Bifidobacterium coryneforme	705403203	---------QR-RQ--E	----	D-VY--LA--T-DD-VI--
	Bifidobacterium crudilactis	736120407	I---------R--E--A	----	D-VY--LA-----D-IA--
	Bifidobacterium cuniculi	705444509	L---------R--E--A	----	--VY--LA----D--VA--
	Bifidobacterium gallinarum	672965335	I---------R--E--A	---M	--V---LA----D--V---
	Bifidobacterium kashiwanohense	746132153	I----I----S--Q--G	----	D-V----A-------E---
	Bifidobacterium merycicum	705456948	L---------R-RE--A	----	--VY---A-----------
	Bifidobacterium mongoliense	705437002	I---------R-RHE-E	RM--	D-VY--LA---YD--V---
	Bifidobacterium psychraerophilum	672987213	I---------R--E--E	----	D-VY--LA-----D-VA--
	Bifidobacterium pullorum	672988254	I---------R--E--A	---M	--V---LA----D--V---
	Bifidobacterium reuteri DSM 23975	672992051	L---------RI-E--A	---V	D-VY---A----D--V---
	Bifidobacterium saguini	727802209	I---------R--Q--A	----	--VY---A-----------
	Bifidobacterium scardovii	672996212	I---------R--E--S	----	--VS---A----D--V---
	Bifidobacterium stellenboschense	673003780	I---------R--EK--	----	--V----A----D--V---
	Bifidobacterium indicum	655535491	I--------QR-RQ--E	----	D-VY--LA--T-DD-VI--
	Bifidobacterium saeculare	672994110	I---------R--E--A	---M	--V---LA----D--V---
	Bifidobacterium stercoris	673001654	-----------------	----	-------------------
	Gardnerella vaginalis	490207977	L---------R--QE-A	R---	--VY---A-----------
	Alloscardovia omnicolens	545373274	M----V--VHK-RE--A	-V--	NPIF--LA-------VA--
	Alloscardovia criceti	516877465	M----V--VHK--A--A	----	NPIF--LA----D--ES--
	Parascardovia denticolens	493331669	M----V--VHQ--V-SA	----	DPIY--LA-----D-RK--
	Scardovia inopinata	493335715	L----V--VHR--A---	----	NPVY--LA----DD--K--
	Scardovia wiggsiae	494250446	M----V--VHR-RA--Q	----	DPVY--VA----DD-----
Other Bacteria 2>500	Brachyspira innocens	518848556	I-D--I---LYI-EKGI		DANYI--T---ID---K--
	Cellulomonas massiliensis	517966923	L---------Q-RDTMP		GAFF--LA----D--V---
	Dermacoccus nishinomiyaensis	664688355	L---------Q--QN-P		DAFF--LA---WD--V---
	Jonesia denitrificans	506251827	L---------Q--ASMP		DAQFI-LA---WD--V---
	Kineosphaera limosa	493641148	L---------Q-RETMP		-A-F--LA---W---E---
	Kitasatospora cheerisanensis	641396826	L---------Q--ESMP		-AQL--LA---WD--V---
	Kribbella catacumbae	521058734	L---------Q-RETMP		-AHF--LA---WD--V---
	Kytococcus sedentarius	506259474	L---------Q-REAMP		-A-F--LA---WD--V---
	Microbispora rosea	663732795	L---------Q-RTTMP		DA-L--LA---W---E---
	Mobilicoccus pelagius	497134043	L-----A---Q-RQSMP		DA-F--LA---WD--V---
	Nicotiana tabacum	10047455	I-D--V----S-RASSL		DAIFI--S-------EK--
	Arthrobacter arilaitensis	503114335	L---------Q--ESMP		-AKF--LA---WD--V---
	Nocardiopsis xinjiangensis	516205353	L---E-----QIRETMP		-A-H--LA---WD--V---
	Oerskovia turbata	663110263	L---------Q-RASMP		-ARF--LA---W---E---
	Phycicoccus jejuensis	663752655	L---------Q-RESMP		DAFF--LA---WD--V---
	Rothia dentocariosa	490102857	I---------QIRESMP		-AQLI-LA---WD--VS--
	Streptosporangium amethystogenes	664383336	L---------Q-RATMP		-A-L--LA--TW---EK--
	Thermobispora bispora	502896635	L---------Q-RAAMP		DA-L--LA---W---E---
	Xylanimonas cellulosilytica	502642059	L---------Q-RESMP		-AHF--LA----A--E---

Supplementary figure S31

Partial sequence alignment of guanylate kinase showing a 4 aa insertion that is mainly found in *Bifidobacteriales*. Additional species which contain a similar CSI are *Nocardioides* sp. Iso805N and *Promicromonospora sukumoe*. Although not shown, this CSI is present in all *Bifidobacterium* species and subspecies.

Group	Species	Accession	202	Insert	244
Bifidobacteriales	Scardovia inopinata	493336643	FVKPSRAGSSFGVTKVDFSQD	KSLLMNR	LSAAVFEAANHDWRV
	Scardovia wiggsiae	656313899	------S--------I-YDD-	SQARTRK	-VE--Y--SQ-----
	Parascardovia denticolens	493333610	-----------------YKA-	ATERQAD	-A---H---R-----
	Alloscardovia omnicolens	545371295	--------------R--WVT-	AAEQEQL	-Q---E---S-----
	Alloscardovia criceti	516876342	--------------R--REE-	AQKQ-HN	-EL--A---E-----
	Bifidobacterium gallicum	493337986	-----------------RAG-	AVSGAAQ	-A---Y---Q-----
	Bifidobacterium longum	494113994	----------------EKAD-	RETQQD-	-A--IAT-GE---K-
	Bifidobacterium breve	489928113	-----------------KAE-	LETQQD-	VA--IAT-GE---K-
	Bifidobacterium angulatum	489924067	----------------EQAD-	EQAQIAA	-E---A--GK---K-
	Bifidobacterium aesculapii	943597546	----------------EQDAT	GDRAAQA	SVL-AAI-EAGKHDWKV
	Bifidobacterium bohemicum	705456116	---------------LENPR-	EAE-AAA	-LE -SR---K-
	Bifidobacterium callitrichos	759442642	----------------EEART	REEQIEK	-GS-IA--GK---K-
	Bifidobacterium merycicum	705459949	----------------EQVE-	EQAQIAA	-G---A--GK---K-
	Bifidobacterium reuteri	763216933	----------------EQAE-	SETQQDL	-A---AT-GE---K-
	Bifidobacterium stellenboschense	736511328	----------------EEADS	PEAQTSQ	-G--IA--GR---K-
	Bifidobacterium saguini	727803346	----------------EKAE-	RETQQDK	-A---AT-GE---K-
	Bifidobacterium adolescentis	547082002	----------------EHEG-	AAE	-A---Y--SR----I
	Bifidobacterium animalis	490388869	----------------EQIG-	AAA	-A------SR-----
	Bifidobacterium bifidum	489908315	----------------EHAG-	AAE	-A------SH----I
	Bifidobacterium boum	651391547	----------------EHEG-	AAE	-A------SH---K-
	Bifidobacterium catenulatum	489932955	----------------EHEG-	AAE	-A------SH-----
	Bifidobacterium choerinum	658450309	----------------ERID-	PEA	-A---Y--SL-----
	Bifidobacterium dentium	489934313	----------------EREG-	AAE	-A------SH-----
	Bifidobacterium magnum	551239529	----------------ERAD-	AEE	-A------SK-----
	Bifidobacterium moukalabense	575769256	----------------EHEG-	AAE	-A------SH-----
	Bifidobacterium pseudocatenulatum	656094433	----------------EHEG-	AAE	-A------SH-----
	Bifidobacterium pseudolongum	551238411	----------------ERVG-	AEE	-A---Y--SL-----
	Bifidobacterium ruminantium	651887370	----------------EHEG-	AAE	-A------SH----I
	Bifidobacterium subtile	639065077	----------------EREG-	AAE	-A---R--SR---K-
	Bifidobacterium thermacidophilum	657872279	----------------EHEG-	AAE	-A------SR---K-
	Bifidobacterium thermophilum	505262704	----------------EHEG-	AAE	-A------SH---K-
	Bifidobacterium tsurumiense	651883056	----------------EHEG-	AEE	-A------SH----I
	Bifidobacterium biavatii	705395628	----------------EHAG-	AAE	-A---YA-YP----I
	Bifidobacterium bombi	763215128	-------------S-LERED-	ERE	-A--L---SQ----I
	Bifidobacterium crudilactis	736121557	---------------IEHDG-	RDE	-L--LL--SS---K-
	Bifidobacterium cuniculi	705448269	----------------ERAD-	PEA	-A---Y--SL-----
	Bifidobacterium gallinarum	705427744	----------------EREG-	AAE	-A------SH-----
	Bifidobacterium kashiwanohense	705411109	----------------EHEG-	AAE	-A------SH-----
	Bifidobacterium mongoliense	705437955	------G------H--ESEG-	G-A	-A---L--SH---K-
	Bifidobacterium psychraerophilum	705399783	-I--------------EHAG-	SKE	-IG-IL---G---K-
	Bifidobacterium pullorum	759447870	----------------EREG-	AAE	-A------SH-----
	Bifidobacterium saeculare	705429707	----------------EREG-	AAE	-A------SH-----
	Bifidobacterium scardovii	705450028	----------------EHEG-	AAE	-A---Y--SH-----
	Bifidobacterium stercoris	673001301	----------------EHEG-	AAE	-A---Y--SH----I
	Bifidobacterium actinocoloniiforme	705421086	----------------EHENA	-D	-AR-L---SR-----
	Bifidobacterium minimum	551240866	--------------R--SPDE	-V	----LA--SR---K-
	Bifidobacterium asteroides	504834239	-------------V--EEP-A	EI	-V---A---K-----
	Bifidobacterium coryneforme	671340521	-------------V--EETGA	DA	-A---D--SG----I
	Bifidobacterium indicum	705390715	-------------V--EETGA	DA	-A---D--SG----I
	Bifidobacterium minimum	551240866	--------------R--SPDE	-V	----LA--SR---K-
	Gardnerella vaginalis	490210715	----------------ESRDA	-A	-AE-I---SE-----
Other Bacteria	Nocardiopsis baichengensis	516126610	----A-----V-I---SDGS-	TDA	VV--IET-RE--PK-
	Actinokineospora enzanensis	517513126	----A-S---------TRKEE		-AE-LAT-REF-TK-
	Brevibacillus borstelensis	489483166	----A-------I---EREDG		-FK-AVN-LEY-PK-
	Desulfitobacterium hafniense	349602227	Y---A-S---------CRQEE		-LS--AI-SQY-SK-
	Enterococcus faecalis	11345396	----A-S---------NGPEE		-N--IEA-GQY-GKI
	Kocuria rhizophila	501366539	----A-------I-R--EPSQ		-D--IAT-RE--LKL
	Lachnospiraceae bacterium	496550687	----A-------I---EEPGV		-AE--TA-LA--SK-
	Nocardia seriolae	586592612	----A-G---I-I----EWD-		-D--IAV-RQ--PK-
	Paenibacillus popilliae	493328360	----A-S------N--NNEDE		-D--IET-RQY-SK-
	Peptoclostridium difficile	545044101	-I--V-------I---IEK-E		-D--IEL-FE--TE-
	Rothia dentocariosa	490103038	----T-------I----KFEE		-VP-IE--RR--AKI
	Ruminococcus bromii	505336871	----V-------I---TEQAE		INT-IES-FE--TE-
	Staphylococcus epidermidis	62083577	----A-S-----L---NGTEE		-N--IEA-GQY-GKI

Supplementary figure S32

Partial sequence alignment of the protein D-alanine--D-alanine ligase showing a 2 to 7 aa insert that is specific for the order *Bifidobacteriales*. The insert length is longer in *Scardovia*-related species and some *Bifidobacterium* spp. Although not shown, this CSI is present in all *Bifidobacterium* species and subspecies.

Group	Species	ID	118		160
Bifidobacterium-Gardnerella clade	Bifidobacterium adolescentis	547081721	ATQIGVDQVIPWQADRSIAKW		RAGRTDKKWRQVLESATEQSRR
	Bifidobacterium angulatum	489923127	-------T-V-----------		KN----R--LSA-QA-------
	Bifidobacterium animalis	490328390	-------E-T---------RF		K-----R--M-T-RA-------
	Bifidobacterium asteroides	504834780	---------------------		-P--N-RH-QAT--A-------
	Bifidobacterium bifidum	489913494	-------E------N------		K-----R------DA-------
	Bifidobacterium boum	651391136	---------------------		K-----R--A-T-DA-------
	Bifidobacterium breve	489925920	---------V-----------		K--------NA--DA----A--
	Bifidobacterium catenulatum	489931773	-------E-------------		KV--------------------
	Bifidobacterium choerinum	639202982	S------D-Y---SE-A--R-		KP----R---AT-MA-------
	Bifidobacterium dentium	489937016	-------E-------------		K-----R------VA-------
	Bifidobacterium gallicum	493337100	-----A-EI---------RF		KQ----R--Q-T-VA-------
	Bifidobacterium longum	658454148	---------V-----------		KV-------NS--DA-------
	Bifidobacterium longum subsp. longum	665772244	---------V-----------		KV-------NS--DA-------
	Bifidobacterium longum subsp. infantis	665782268	---------V-----------		KV-------NS--DA-------
	Bifidobacterium magnum	551239111	-------S-V---------R-		K-----R--QA--HA-------
	Bifidobacterium moukalabense	575769998	-------E-------------		K-----R-------A-------
	Bifidobacterium pseudocatenula	656093744	-------E-------------		K------------DA-------
	Bifidobacterium ruminantium	651886209	-------L-V-----------		KV----R--K-T----------
	Bifidobacterium sp. 7101	658453313	---------M-----------		-P--N-RH-QAT-DA-------
	Bifidobacterium sp. A11	639449903	---------M-----------		-P--N-RH-HAA-DA-------
	Bifidobacterium subtile	639061773	-------A------E------		KE--S-R----T-A--------
	Bifidobacterium thermacidophilum	657872212	-------T-------------		KR--S-R--S-TIDA-------
	Bifidobacterium thermophilum	505263401	-------T-------------		KR--S-R--S-TIDA-------
	Bifidobacterium tsurumiense	651882291	-------T-V-----------		KQ----R--G---DA-------
	Gardnerella vaginalis	518091554	-------E----C-N---S--		KP-----R-N----A-------
Other *Bifidobacteriales*	Alloscardovia omnicolens	545375335	--E----S-M---SQ---VQ-	KGN	K-EKALR--KD--V--------
	Metascardovia criceti	648490216	--E----K-----SQ---VQ-	KGN	K-QKALN--QDLVTG-S-----
	Parascardovia denticolens	493332022	--E----R------Q---VL-	KGS	K--KGRE---DQ-IA-------
	Scardovia inopinata	493336060	--E----FIL--------VQ-	KGP	K-A-GLR--QDQ-IM-------
	Scardovia wiggsiae	494249210	-VE--A-A----------VQ-	KGP	--AKGRE--HDR-VM-A-----
Other Bacteria	Blastococcus saxobsidens	504188315	--EL---RI-----S-CVTR-	RDD	-VAKGVA---SAAR--AK----
	Brachybacterium squillarum	498220588	--EL---A----I-G--VSV-	RGE	KLRKGRAR-ESAVRA-VK----
	Brevibacterium album	656050642	--E----RIV----A----R-	PAK	KEQ-AHAR--SL-DA-AQ----
	Catenulispora acidiphila	506275839	M-E----A-V--A-S---VQ-	KGE	-GEKALN---ATARE-AK----
	Cellulomonas flavigena	502882457	--EV---V-V----E---VV-	RGE	--QKSRAR-IGTVRT--K-A--
	Cellulosimicrobium cellulans	640258030	--E----V-V----E--VVV-	RGE	--AKSRAR-LATVRT--K-A--
	Clavibacter michiganensis	505303143	--EL---A------Q--VSR-	EGA	KVAKGRER--AIVRE-VK--I-
	Corynebacterium durum	492955973	---A-A-VIV----S-CV---	QGA	KREKGVA---AMAV--AK----
	Dermatophilus congolensis	654875752	--EL---EI----SR-A-VQ-	KGP	--EKALR--QSQV-A-AK----
	Dietzia alimentaria	498226327	--EA-A-KI-------CVSR-	TGP	KIDKGRT--INAARA-GK----
	Intrasporangium calvum	503258306	--EL---E-V--A-E---VV-	RGE	--AKSLA--MT-VTR-AK----
	Isoptericola variabilis	503605030	-VEV---A-V----E---VV-	RGP	--AKSHAR-VATVRA-VK-A--
	Janibacter hoylei	495201504	--EL---T-V--------VR-	KGP	KVDKGLR--AN-V-R-AK----
	Jonesia denitrificans	506252049	--EV---R----------VV-	RGA	--AKSHAR-VATVR--MK-A--
	Kytococcus sedentarius	506259380	SVE----H-L--E-E---SR-	SGP	K-AKGRA--AG-VRA--K----
	Leifsonia aquatica	545658391	--EL---R-V--S-S--VSR-	EGA	KIAKGRDR-STIVRE-AK--I-
	Ornithinimicrobium pekingense	551300767	--ELD--E-V--------VR-	KGE	-GEKARG--E-TARA--K----
	Phycicoccus jejuensis	663753660	--EC---E-V----A---VQ-	RGE	-GEKARR--DA--VA--K----
	Promicromonospora sukumoe	518858037	--EV-A-AI-----E--VVV-	RGD	--AKSRAR-VSTVRA--K-A--
	Rhodococcus wratislaviensis	589263243	--EA---AIV---SS-CV-R-	EGP	KVAKGVAR--SAALA-AK----
	Turicella otitidis	490738030	---A--GEIV----E-CV-R-	SGP	K-KKGRE--LATARE-AK----
	Varibaculum cambriense	551244706	-VEL-ISR-R--A-G---VQ-	KGA	KVQ-GA---ESL-QA-AK----
	Actinomyces viscosus	490701626	--EV--GL-L---SE-CVSV-	QGA	KQAKGRQR-QATALQ-AK-A--
	Actinotalea ferrariae	601042671	--EV---E-V----R--VVV-	RGE	--A-SRA--VATVRQ--K----
	Arsenicicoccus bolidensis	656266779	--EL---E-V--G-A---VQ-	RGD	--AKAHR--E--VTA--K----
	Arthrobacter aurescens	500098911	--EL-I-A-----SE-A-VR-	KGE	--AKAHA--QS-VTA-AK-A--

Supplementary figure S33

Partial sequence alignment of ribosomal RNA small subunit methyltransferase E showing a 1 aa insert that is specific for the *Bifidobacterium-Gardnerella* clade of the order *Bifidobacteriales*.

Group	Organism	ID	309	Insert	354
Bifidobacterium-Gardnerella clade	Bifidobacterium adolescentis	547055080	DFEKGFIKADIVSYDDFVAA	E	GSMTKIKEEGKLRQEGRDYVMQDGD
	Bifidobacterium angulatum	489923263	--------------------	D	---VT--------------------
	Bifidobacterium animalis	490328214	---------EV-------E-	Y	---A---------L--K--------
	Bifidobacterium asteroides	504834899	---------EV---N-----	D	--YA-VR----M-L--K--------
	Bifidobacterium bifidum	489910184	---R----------------	-	---V---------------------
	Bifidobacterium boum	651390721	---R-----E----------	N	---NT--------L-----------
	Bifidobacterium breve	489925740	--------------------	D	---A------------------A---
	Bifidobacterium choerinum	639202807	---------EV---------	D	---P---------L--K--------
	Bifidobacterium dentium	489937396	--------------------	-	-------------------------
	Bifidobacterium gallicum	493336959	----------V---------	N	--IPA--------I--K--------
	Bifidobacterium longum	494111296	--------------------	D	---A------------------A---
	Bifidobacterium magnum	551238756	----------V-------G-	Q	---P---D-----I--K--------
	Bifidobacterium minimum	651887894	---------EV---------	K	---AA---Q-R--L--------P--
	Bifidobacterium moukalabense	575769740	--------------------	-	---A---------------------
	Bifidobacterium pseudocatenulatum	656093871	--------------------	-	---V---------------------
	Bifidobacterium pseudolongum	551238204	---------EV---------	D	---A---------L--K--------
	Bifidobacterium ruminantium	651886067	--------------------	D	-------------------------
	Bifidobacterium sp. 7101	658453021	---------EV---------	D	--YA-VR----M-L--K--------
	Bifidobacterium sp. A11	639449247	---------EV---------	D	--YA-VR----M-L--K--------
	Bifidobacterium sp. AGR2158	651885385	---------EV---------	D	---P---------L--K--------
	Bifidobacterium sp. MSTE12	570844168	---T----------------	-	-------------------------
	Bifidobacterium subtile	639061423	---------E----------	-	-------DA----L-----I-----
	Bifidobacterium thermacidophilum	657871838	---R-----E----------	N	---NT--------L-----------
	Bifidobacterium thermophilum	505263227	---R-----E----------	N	---NT--------L-----------
	Bifidobacterium tsurumiense	651882898	---------SV---------	H	---NA--------L-----------
	Gardnerella vaginalis	523600424	---R-----EV-------E-	Q	---A---------L-----------
	Bifidobacterium psychraerophilum	705398485	---------E------LL--		--YAKV-------L--KE-------
	Bifidobacterium crudilactis	736120967	---R-----E---FN-L-E-		--YAKV-----M-L--K--I-H---
Other *Bifidobacteriales*	Alloscardovia omnicolens	545373955	---R-----A---FE-L-E-		--YA-V-----M-L--K----R---
	Metascardovia criceti	516878091	---R-----E---F--L-E-		--YA-V-------L--K--------
	Parascardovia denticolens	493331913	---R-----EV-----L---		--Y--V-----M-L--K----R---
	Scardovia inopinata	493335938	---R-----EV-----L---		--YA-V-----M-L--K--------
	Scardovia wiggsiae	494249657	---R-----EV-----L---		--YP-VR----M-L--K--I-----
Other Bacteria	Acaricomes phytoseiuli	648482722	--QR-----EV--F--L-D-		---AEA-SR--V-I--K----H---
	Actinopolymorpha alba	522069078	--QR-----EV-----LIE-		---HEA-AR--V-L--K----A---
	Aeromicrobium marinum	494137303	---R-----E---F--L-E-		---AAA-SV--V-M--K----A---
	Amycolatopsis thermoflava	654463709	---R-----EV--F--L-E-		---AAARSA--V-M--K--I-A---
	Capnocytophaga sputigena	488754920	-------R-EVIA----ITY		--EA-V--A--M-V--KE-IVK---
	Cellulophaga algicola	503318224	-------R-EVIA---Y--H		--EA-V--A--M-V--KE-IVK---
	Clavibacter michiganensis	505303758	---------EVI----L-ET		--IAEARSK--A-I--KE-------
	Desulfotomaculum reducens	500207417	---R---R-EV-----L-T-		---A-AR-A----L--K---V----
	Kocuria palustris	493246086	---R-----E---F--L---		---ADA-AA--V-M--K--------
	Kribbella flavida	502687600	--QR-----E---F--L-E-		---AAA-SA--V-M--K----A---
	Lactobacillus plantarum	489738334	---R---R-EVM---ALDE-		--EA-V--N----L--K--------
	Microlunatus phosphovorus	503631132	--Q------EV--F--L-E-		---QAA-AK--V-L--K----A---
	Mobilicoccus pelagius	497129562	--Q------E------L---		---AEA-SA--V-M--KE-------
	Mycobacterium triplex	620041625	---------E------LID-		---VAA-AA--V-M--K----A---
	Nocardioides alkalitolerans	655023748	--Q------E------L---		---A-A--A--V-M--K--I-A---
	Ornithinimicrobium pekingense	551300283	---R-----EV--F--LD--		-T-Q-A--A-RV-I--K--------
	Paenibacillus taiwanensis	655096106	---R---R-EV--F--L--G		---NVV--Q----L--KE--V----
	Propionimicrobium lymphophilum	512610324	--Q------EVM----L--L		--EQEV-AA--M-L--K--------
	Pseudonocardia asaccharolytica	655577688	---R-----EV--F--L-E-		---AAA-SA--V-M--K----V---
	Rhodococcus hoagii	491657888	---R-----E---F--L-E-		---NAA-SA--V-M--K--I-----
	Sanguibacter keddieii	502630994	---R-----EV--F--L-E-		--VAAARSA--A-I--K--------
	Spirillospora albida	663124325	--QR-----E---F--L-E-		---ADARSA--V-M--KE-------
	Streptomyces sclerotialus	664084913	--Q------EVI----L-EC		--VAEARSA--A-M--K--I-----
	Symbiobacterium thermophilum	499510424	-M-R---R-EV-----L-T-		---AAAR-A--V-L--K--------

Supplementary figure S34

Partial sequence alignment of the GTP binding protein YchF showing a 1 aa insert that is specific for the *Bifidobacterium-Gardnerella* clade of the order *Bifidobacteriales*. A similar insert in this protein is also present in *Ruminococcus* sp. CAG:254

Group	Species	ID	730		765
Bifidobacterales except *Scardovia*-related species	Bifidobacterium adolescentis	500062679	AGAPPTDWTLYDTHYTERYLG		LDPTTYERNSIVADA
	Bifidobacterium angulatum	489921825	---------------------		---VV------I---
	Bifidobacterium animalis	490328922	-------------C----F--		---QV-AD----L--
	Bifidobacterium asteroides	504834022	---------------------		---AV-R--G-ID--
	Bifidobacterium bifidum	489912744	---------------------		---AA------I---
	Bifidobacterium boum	651391206	---------------------		---E--RA---IE--
	Bifidobacterium breve	644611216	---------------------		---DV-Y--G--Q--
	Bifidobacterium catenulatum	489930297	---------------------		---AV------IS--
	Bifidobacterium choerinum	639202051	-------------C----F--		--EAD-V----ID--
	Bifidobacterium dentium	489934738	---------------------		---VV----G-I---
	Bifidobacterium gallicum	493338609	---------------------		---AV------I---
	Bifidobacterium longum	501572122	---------------------		---DV-R--G--Q--
	Bifidobacterium magnum	651390279	---------------------		-EER---D---IK--
	Bifidobacterium moukalabense	575769035	---------------------		---AV----G-I---
	Bifidobacterium pseudocatenulatum	547836833	---------------------		--SAV------I---
	Bifidobacterium pseudolongum	651884092	-------------C-------		---EV-A----I---
	Bifidobacterium ruminantium	651887039	-----A---------------		-------H---I---
	Bifidobacterium sp. 7101	658453597	---------------------		---AV-R--G-ID--
	Bifidobacterium sp. A11	639449485	---------------------		---AV-R--G-ID--
	Bifidobacterium sp. AGR2158	651885207	-------------C-------		I-EAD-V-----D--
	Bifidobacterium sp. MSTE12	570842730	---------------------		---VV----G-I-G-
	Bifidobacterium subtile	639064094	---------------------		---AV------I---
	Bifidobacterium thermacidophilum	657871651	---------------------		---E--RA---IE--
	Bifidobacterium thermophilum	505262462	---------------------		---E--RA---IE--
Other Bacteria	Actinokineospora enzanensis	517516953	----V---RI-----------	HPD	TH-EV-D----ID--
	Actinomadura atramentaria	518459877	----V---R------------	HPD	EE-EA-AAQ-LIDR-
	Actinopolymorpha alba	522069057	----V---R----C-------	HPE	ER-EV-----LL-S-
	Actinopolyspora halophila	648460581	----VC--R------------	DPN	TS-E--AA--LLS--
	Actinosynnema mirum	506284582	----V---R------------	DPA	ER-EV-DA--LID--
	Amycolatopsis alba	651355529	----V---S------------	LPQ	K-AAS--H--LI-G-
	Kitasatospora setae	503905775	-------FR----A-------	LPQ	EN-EG-AADCLID--
	Kutzneria albida	644678312	----V---R------------	HPQ	ER-EV-DA--LIS--
	Nocardiopsis alba	504725030	----VI--A------------	TPQ	EQKEV---L-L-S--
	Pseudonocardia acaciae	655571746	----V---R------------	DPA	-Q-EA---S-LL---
	Saccharomonospora azurea	491581794	----V---S----A-------	HPA	EH-DV--H--LI---
	Saccharopolyspora erythraea	497628205	----VC--R------------	DPN	DV-EV--A--LMR--
	Saccharothrix espanaensis	504917027	----V---R------------	HPD	EK-EV-DT--LI---
	Sciscionella marina	648505053	V---V-E-E------------	TPE	EN-QV-H---VTGE-
	Smaragdicoccus niigatensis	516906162	S---V---A------------	DPG	QQ-DV-----LI---
	Streptomyces acidiscabies	498036394	V---V--LR------Q-----	HPA	EQ-DV-R---L-D--
	Streptosporangium roseum	502655592	-------FRW---A-------	LPE	ENASG-DGD-LI---
	Thermobifida fusca	499610198	----V---R------------	HPD	D--EC---E-LLV--
	Thermocrispum agreste	655463312	----V---K------------	HPD	EN-EA-RA--LID--
	Thermomonospora curvata	502614566	----V---R------------	HPD	EE-DN-R---LIE--

Supplementary Figure S35

Partial sequence alignment of cytochrome c showing a 3 aa deletion that is specifically found in the *Bifidobacterium* species. The homologs of this protein were not found *Scardovia*-related genera.

Group	Organism	Accession	37	Insert	67
Scardovia clade	Scardovia inopinata	493335662	IGRKPIRPLP	Q	EEGRERLDIVDDTKSVSKRH
	Scardovia wiggsiae	494250531	----------	E	----T----I----------
	Parascardovia denticolens	493334112	L---------	A	----T----E-S-R-M----
	Alloscardovia omnicolens	551236833	-----M--VE	V	PDNAT-F-VP-L---M--N-
Other *Bifidobacteriales*	Bifidobacterium adolescentis	489904441	V----L---S		DD-FA----M-PG--M----
	Bifidobacterium animalis	490389059	-----L----		DD-KR-VE-L--SR------
	Bifidobacterium asteroides	504835298	-----L----		DD--R--EVP-Q---M----
	Bifidobacterium bifidum	489911020	-----L----		DD-T---E-A-G-R-M----
	Bifidobacterium breve	489926499	-----L---A		DD-MQ---V--Q---M----
	Bifidobacterium catenulatum	489932743	-----L---V		DD-FA----M-AS--M----
	Bifidobacterium choerinum	658450028	-----L--V-		SG-MR-IE-L---R-M----
	Bifidobacterium dentium	489934116	L----L---A		DD-YS----A-SN--M----
	Bifidobacterium gallicum	493338357	---------A		DD-TV-VE-P-A-R-M----
	Bifidobacterium longum	494113000	-----L---A		DD-NT---VA-Q---M----
	Bifidobacterium magnum	651390239	----------		QGLYR-VE-T---R-M--K-
	Bifidobacterium minimum	551240453	---R----IA		DSSMT-VE---PAR-M----
	Bifidobacterium moukalabense	575769355	L----L---A		DD-YS----T-AN--M----
	Bifidobacterium pseudocatenulatum	490330642	-----L---V		DD-FA----M-AN--M----
	Bifidobacterium pseudolongum	651888579	-----L---S		DD-TT-VE-L---R-M----
	Bifidobacterium ruminantium	651886886	-----L----		-D-FS----Q-PN--M----
	Bifidobacterium sp. 12_1_47BFAA	496058900	-----L---A		DD-NT---VA-Q---M----
	Bifidobacterium sp. 7101	658452647	-----L---A		DD--R--EVP-K---M----
	Bifidobacterium sp. A11	639448752	-----L---A		DD--R--EVP-Q---M----
	Bifidobacterium sp. MSTE12	570844656	L----L---A		DD-YS----A-SN--M----
	Bifidobacterium subtile	639065323	-----L----		DD-F--ME-E-SSR-M----
	Bifidobacterium thermacidophilum	657871220	-----L---A		DD-NR--EVP-SER-M----
	Bifidobacterium thermophilum	505263898	-----L---A		DD-NR--EVP-SER-M----
	Bifidobacterium tsurumiense	651881836	---------A		DD-FT-IE-E-S-R-M----
	Bifidobacterium animalis	757775334	-----L----		DD-KR-VE-L--SR------
	Bifidobacterium reuteri	763216701	-----L---A		AN--T---V--Q---M----
	Bifidobacterium actinocoloniiforme	917298769	-----L----		DD-MV--EVQ-K---M----
	Bifidobacterium cuniculi	672968277	-----L--V-		D--IR-IE-L---R-M----
	Bifidobacterium longum	924458288	-----L---A		DD-NT---V--Q---M----
	Bifidobacterium stellenboschense	736511123	-----L----		D--VA--E-D-A---M----
	Bifidobacterium longum	695823851	-----L---A		DD-NT---VA-Q---M----
	Bifidobacterium psychraerophilum	917315015	-----M--IA		NDEGK---VP-QS--M----
	Bifidobacterium aesculapii	943597381	-----L----		D--IA--E-D-A---M----
	Bifidobacterium saguini	727803282	-----L---A		AD-N----VA-Q---M----
	Bifidobacterium sp. 12_1_47BFAA	496058900	-----L---A		DD-NT---VA-Q---M----
	Bifidobacterium merycicum	672974012	-----L----		DD-TK-ME-P-P---M----
	Bifidobacterium pullorum	917516368	-----L---A		DD-MS--E-P---R-M----
	Bifidobacterium sp. AGR2158	916591281	-----L--V-		SG-MR-IE-L---R-M----
	Bifidobacterium angulatum	672945166	-----L----		DD-TT-MEVE-S---M----
	Bifidobacterium indicum	705389108	-----L---G		DD--R--EVP-G---M----
	Bifidobacterium coryneforme	705402502	-----L---G		DD--R--EVP-G---M----
	Bifidobacterium kashiwanohense	672968189	-----L---V		DD-FA----M-AN--M----
	Bifidobacterium callitrichos	672959667	-----L----		DT-VA--EVE-SA--M----
	Bifidobacterium crudilactis	736119783	-----M--IA		GDDVR---VP-S---M----
	Bifidobacterium stercoris	673001402	-----L---S		DD-FV----M-PG--M----
	Gardnerella vaginalis	490207155	---------R		-D-FA-V----NKR-M----

Supplementary figure S36

Partial sequence alignment of FHA domain protein showing a 1 aa insert that is specific for the *Scardovia* clade.

Group	Species	ID	23		67
Scardovia clade	Metascardovia criceti	648490110	SAAQFAHELERLGHEVRLVGVDAP	DK	RYRAREQYIPVVTHFAKPH
	Scardovia inopinata	493336366	--N-L-A--T----Q------Q--	EP	S---E-LHL--A-AL-HQQ
	Scardovia wiggsiae	494248709	--Y-L-R--TGR-NT---I--G--	-A	Q------H---A-W--HRQ
	Alloscardovia omnicolens	545373074	--V---A----Q--Q-----IG--	VE	E-K-E-R-VFGA-E--R--
Other *Bifidobacteriales*	Gardnerella vaginalis	896210747	--V---A----Q--Q-----IG--	CD	D-K-Q-R-VFGA-E--R--
	Bifidobacterium adolescentis	547081098	--L-W-Q----Q--H-----IG--		E-P--VNHV-L-SWV--KQ
	Bifidobacterium angulatum	489922423	--L-W-Q----Q--T-----IG--		V-A--VHHV-L-SWV-AKQ
	Bifidobacterium animalis	490329449	--L-W-Q----Q--K-----IN--		E-P---NH--L-SWISAKQ
	Bifidobacterium asteroides	504835059	--I-Y-QG--A---R------GST		D-P--VHR--L-SAV-ARQ
	Bifidobacterium bifidum	489910475	--L-Y------Q--H-----IG--		E-P--VNKV-L-SWV-AKQ
	Bifidobacterium breve	489925308	--L-W-A----Q--H------G--		E-P--VNKV-L-SWV-AKQ
	Bifidobacterium catenulatum	489933136	--L-W-E--K-Q--T-----IG--		E-P--VNHV-L-SWV--QQ
	Bifidobacterium choerinum	658449950	--T-W-A--Q-Q--------IG--		E-P---NH--L-SWISAKQ
	Bifidobacterium dentium	489933540	--L-W-Q----Q--H-----IG--		DFP--VNHV-L-SWV--QQ
	Bifidobacterium gallicum	493338038	--L-W-QQ-K-E--H-----IG--		E-P-Q-NL--F-SWISRKQ
	Bifidobacterium longum	494110424	--L-W-A----Q--H-----IG--		E-P--VNRV-L-SWV-AKQ
	Bifidobacterium magnum	551239253	--L-W-Q--R-Q--T-----IG--		Q-S-K-NHV-L-SWI-SKQ
	Bifidobacterium moukalabense	575769650	--L-W-Q----Q--H-----IG--		DFP--VNHV-L-SWV--QQ
	Bifidobacterium pseudocatenulatum	490330099	--L-W-E--K-Q--T-----IG--		E-P--VNHV-L-SWV--KQ
	Bifidobacterium pseudolongum	651883673	--L-W-Q----Q--H-----IN--		E-P-KVNRV-L-SWV-AKQ
	Bifidobacterium ruminantium	651886721	--L-W-E----Q--H-----IG--		E-P--VNHV-L-SWV-RKQ
	Bifidobacterium sp. 7101	658453177	--I-Y-QG--A---Q------GST		D-P--VHR--L-SAV-ARQ
	Bifidobacterium sp. AGR2158	651884801	--T-W-A--R-Q--------IG--		E-P---NR--L-SWISAKQ
	Bifidobacterium sp. MSTE12	570844491	--L-W-Q----Q--H-----IG--		DFP--VNHV-L-SWV--QQ
	Bifidobacterium thermacidophilum	657871433	--L-Y-K--R-Q--T-----IGS-		E-PI-VRH--L-SWI-AKQ
	Bifidobacterium thermophilum	505263608	--L-Y-K--R-Q--T-----IGS-		E-PI-VRH--L-SWI-AKQ
	Bifidobacterium tsurumiense	651882790	--L-Y-Q----Q--H-----IGSQ		E-P-KVNR--L-S-I-ARQ

Supplementary figure S37

Partial sequence alignment of glycosyl transferase showing a 2 aa insert that is specific for the *Scardovia* clade. Additionally, this insert is also present in *Gardenella*.

Group	Species	Accession	30		77
Scardovia clade	Scardovia inopinata	294458767	NDAGQAASGTVRHLLQAL	EV	TVQEVDHICCSTYYDYQYTRPLMCTVDG
	Scardovia wiggsiae	494249115	---A---T-AL----K--	-I	ASY--GR-RS-SFF--RS---VI-C-E-
	Alloscardovia omnicolens	545375611	----L--TQALHF--R-Y	SQ	SAH-------GSF---TDY--VIVS-E-
	Metascardovia criceti	516878295	-----V-THALN--ISTY	AS	QSR-------GSF---TS---V-VN---
	Parascardovia denticolens	493332070	---------AI----K--	--	ESRQ--R-S--PFF---F---M------
Other *Bifidobacteriales*	Gardnerella vaginalis	896227146	----L--TQALHF--R-Y	SQ	SAH-------GSF---TDN--VIVS-EG
	Bifidobacterium adolescentis	547055586	---S---TNV----VSRY		ES---R--DNEGF----VA--MI-S-Q-
	Bifidobacterium angulatum	489922952	---C---TNAIHQ-ID-Y		DAR--Q--S-EG-----VS--TL-R-N-
	Bifidobacterium animalis	490328484	---C-S-TDVI---VSRY		RSL-IG--NKDG-----SA--MQ--IQ-
	Bifidobacterium asteroides	504834721	---CS-STNVI----NVY		ESH-IGS-PGDGF----MS--ML-H-Q-
	Bifidobacterium bifidum	489909828	---C---TNVI---VSRY		DSR-IR--R-DG-----VA--ML-K-T-
	Bifidobacterium boum	651391065	---S----EVIHY-ISHY		ESRL-YE-NSDG-----S---ML-HIT-
	Bifidobacterium breve	489926015	---C----NV--Y-VKHY		ESR-IR--D-DAF----VS--ML-H-S-
	Bifidobacterium catenulatum	489931467	---C---TNVI---VSRY		DS---C--DNEGF----VA--MI-S-Q-
	Bifidobacterium choerinum	639202910	-----S-TNV----VSRS		PML--G--NRDG-----TA--MQ-VIQ-
	Bifidobacterium dentium	489936843	---C---TNVI---VSRY		DS--IR--NDEGF----VA--MI-S-Q-
	Bifidobacterium gallicum	493337653	---D-C-TDVI-Q-VSHY		PSV--G--NRDG-----VS--IQ-SIQ-
	Bifidobacterium longum	494112071	---C---TNA----VKRY		ESR-IR--R-DDF----VA--ML-H-S-
	Bifidobacterium minimum	551240276	---C-T-TNV--RMVAHY		ESR--R--S-DG-----TA--M--H-T-
	Bifidobacterium moukalabense	575770095	---C---TNVI---VSRY		DS---R--NDEGF----VA--MIYS-Q-
	Bifidobacterium pseudocatenulatum	490332701	---C---TNVI---VSRY		DS---H--DNEGF----VA--MI-S-Q-
	Bifidobacterium pseudolongum	651883359	---C-S-TNVI---VSKY		RSL--G--SRDG-----TS--MQ-VIQ-
	Bifidobacterium ruminantium	651886389	---N---TNVI---VSRY		ES---R--DNEDF----VA--MI-S-Q-
	Bifidobacterium sp. 12_1_47BFAA	496059016	---C---TNV----VKRY		ESR-IR--K-DDF----VA--ML-H-S-
	Bifidobacterium sp. 7101	658453381	---CS-STNVI----NVY		ESH-IGS-PGDGF----MS--ML-H-Q-
	Bifidobacterium sp. A11	639450066	---CS-STNVI----NVY		ESH-IGS-PGDGF----MS--ML-H-Q-
	Bifidobacterium sp. AGR2158	651885663	--V--S-TNV----VSKF		HAL--G--SRDG-----TA--IQ-VIQ-
	Bifidobacterium subtile	639063942	---C---TNV----IGRY		DAI--R--R-DA-----V---MS-M-S-
	Bifidobacterium thermacidophilum	657872139	---S----EVIHY-IGHY		ESKLIHE-NSDG-----A---ML-HIT-
	Bifidobacterium thermophilum	505263486	---S----EVIHY-IGHY		ESKLIHE-NSDG-----A---ML-HIT-
	Bifidobacterium tsurumiense	651882142	---C--ST-VI---MSHY		ESR--R--R-DGF----S---MV-H-T-
Other Bacteria Indel (-)	Brachybacterium muris	516433463	----E----AIE---SVW		PSRLAGAVDAEEFI-F-VN--ELR-TE
	Catelliglobosispora koreensis	522010859	---AD--TSAIE--E-LW		DAR-ITA-DPEEF--F-VN--QITL---
	Gordonia sputi	491344981	----D---SA-E--ALTW		DA-PLVD-DADD---F-VN--TVKQI--
	Leifsonia aquatica	545654142	----E----A-KT-KEQ-		D-VPIAEVDPEL-F-F-FN--VVEEL--
	Nocardia brasiliensis	659854659	----D----A-E--ELIW		DAEPLAELDSED-----VN--TVRQ---
	Nocardioides alkalitolerans	655024208	----D----V-D--ME-W		SARV-GA-DPEDF--F-MH--VVG-
	Nocardiopsis xinjiangensis	516202738	----E---LA-E--SSQW		EAHRLCALTPDD---F-VA--QLSV---
	Actinoalloteichus spitiensis	515068645	----D---AAIE--QLSW		DASPLTELDPEE---FTV---TLSL---
	Actinokineospora enzanensis	517510596	----D---TAIE--QLTW		DASPLLE-DPDD---F-V---TVRM---
	Actinomadura atramentaria	518463632	----E----V----AA-W		ESVP-AEVDPED---F-V---TVELT--
	Humibacter albus	551279171	----E----A--T-KD--		D-VP-AGVDPEL---F-FN--TIASD-
	Lechevalieria aerocolonigenes	663694443	----D---TAIE--QLTW		DATPLAE-DPDD---F-V---TVRM---
	Actinomycetospora chiangmaiens	517144693	----D-S-TA-E--ALNW		DAT-LGA-DPED---F-VS--TVRL---
	Actinosynnema mirum	506281266	----D---TAIE--QLTW		EAKPLAE-DPDE---F-V---TVRM---
	Agromyces subbeticus	651369673	----E--T-AAKL-VER-		GLV--AAVDPEL-F---F---TIVVG-
	Amycolatopsis balhimycina	522127077	----D---RA-E--QLNW		DATQLAELEPDD---F-VS--TVRM---
	Pseudonocardia dioxanivorans	503440776	----E---TALE--ELSW		DATPL-S-DPED---F-V----VRLS--
	Rubrobacter xylanophilus	499883327	---AE---MALSA-GN-W		G-RRFGRFDPEEFF---S---QVKL---
	Saccharopolyspora erythraea	497631743	----D---SAIE--QLTW		DATPLSE-DPDP---F-VS--TVKL---
	Serinicoccus marinus	551304026	----EC--SV-E--A-VW		DAEV-AA-DPEDF--F-VN--QVL----
	Spirillospora albida	663126904	----E----VI---ETTW		DAVPIAELDPDD---F-V----VEM---
	Thermocrispum agreste	655463113	----E---AGIE--HRCW		QATK-AELDPDD---F-V---TVHM---
	Thermomonospora curvata	502615723	----E----VI---EA-W		QAAP-AELDPED---F-V---TIELT-
	Tsukamurella paurometabola	502891800	----D----A-E--ELIW		DA-PLTELDSDDF--F-VN--TIKQI--

Supplementary figure S38

Partial sequence alignment of a PAC2 family protein showing a 2 aa insert that is largely specific for the *Scardovia* clade of *Bifiodbacteriaceae* family. In addition to the *Scardovia* clade, a similar insert is also present in *Gardnenella vaginalis* and *Catenulispora acidiphila*.

			167		206
Scardovia clade	Scardovia inopinata	493336671	IFTGSITRWNDPAIKKENPS	LA	AKLPDISVTPVWRSDKSG
	Alloscardovia criceti	516877756	V-Q-K--M----E-QA----	I-	-Q--ELEI----------
	Alloscardovia omnicolens	545371909	--N---S-------IA----	I-	----NLAI----------
	Parascardovia denticolens	493332593	--S-A-------E-ASQ---	--	SR--ALDI--I-------
Other *Bifidobacteriales*	Bifidobacterium adolescentis	500062874	--D-K--K---D----Q--K		VD---LDI-V-H------
	Bifidobacterium angulatum	489924226	--D-K--K---A---SQ--K		LN---TDI-V-H------
	Bifidobacterium animalis	504510161	V-D-R------A-LTAQ--G		LS---LPI-V-H------
	Bifidobacterium bifidum	489913155	--D-K-VK------ANQ-KD		LA---TQI-V-H------
	Bifidobacterium bifidum	503129229	--D-K-VK------ANQ-KD		LA---TQI-V-H------
	Bifidobacterium boum	651390604	--D-K--K---D---QQ--K		VD---TAI-V-H------
	Bifidobacterium breve	489926490	--D-K--K------ADQ-KD		LT---TAI-V-H------
	Bifidobacterium catenulatum	489932755	--D-K--K---D---QQ--K		VD---LDI-V-H------
	Bifidobacterium choerinum	639202605	--D-K------A-LARQ--D		LE---LPI-V-H------
	Bifidobacterium dentium	489934137	--D-K--K---D---QQ--K		VD---LDI-V-H------
	Bifidobacterium gallicum	493338370	--N--------A-LQRL--N		IT---LPI-V-H------
	Bifidobacterium longum	501572381	--D-K--K------AEQ-KD		L----TDI-V-H------
	Bifidobacterium magnum	651390238	--N----Q------A-Q--D		VT--HLPI-I-H------
	Bifidobacterium moukalabense	575769346	--D-K--K---D---QQ--K		VD---LDI-V-H------
	Bifidobacterium pseudocatenulatum	490330651	--D-K--K---D---QQ--K		VD---LDI-V-H------
	Bifidobacterium pseudolongum	551238553	V-N-E---------TEQ--G		LD--NLTI-V-H------
	Bifidobacterium ruminantium	651886894	--D-K--K---N---SQ--K		VD---LDI-V-H------
	Bifidobacterium sp. AGR2158	651885002	V-A-R------ER-AAQ--D		L----LAI-V-H------
	Bifidobacterium thermacidophilum	657871213	--D-K--K---D----Q--K		VD---TAI-V-H------
	Bifidobacterium thermophilum	505263908	--D-K--K---D---QQ--K		VN---TAI-V-H------
	Gardnerella vaginalis	490207178	--D-K------DD---Q--K		L----TPI-V-H------
Other Bacteria	Agromyces subbeticus	651370097	--A-T-SN------AAT--D		-T---LAI---H---D--
	Arthrobacter globiformis	489895958	--R-E-AK------AAL--D		-----LA----S---D--
	Brachybacterium faecium	506256772	--S-D--N------AEH--D		-D---LEIV--H---D--
	Brevibacterium casei	496836776	--K-E--N---A---AD--D		-T---LKI-A-H-A-D--
	Campylobacter fetus	488937116	--S-K--K---EI---D-AN		L----ALI---V---S--
	Dermatophilus congolensis	654875195	--S-N--K----K-IAD--G		KTM--LTI---H---E--
	Draconibacterium orientale	610422643	--M-E--N-------AN-EG		LA---MEI-F-H---G--
	Frankia alni	499926841	--A-K--K-------A--SA		-S---ATIAS-H---G--
	Geodermatophilus obscurus	502714467	V-D-R--T------AAD--G		-S---LQI---N---E--
	Gordonia alkanivorans	493402579	--N-K--K------AAL--G		VQ--GGDIV--Y---S--
	Janibacter hoylei	495202508	--S-K-KT------A---EG		VD---TKI---N---E--
	Jiangella gansuensis	652479950	--G-T--N------AAD--E		-T---LAI---H---E--
	Kineosphaera limosa	493640683	--D-K--T------AAD--G		VT--ATNI---H------
	Kocuria palustris	493242673	--S-E--T------AQQ--D		-D---TNI-V-H---E--
	Kutzneria albida	644697850	--S-Q--N---A----L---		-S---LKIAV-Y---E--
	Lamprocystis purpurea	648507880	--S-T--K------AAT--G		VA---KAI-V-T---S--
	Leifsonia aquatica	545658321	----V--N-K--Q-A-D--G		LAM--KAIV--V---G--
	Leucobacter chironomi	636831446	--N----K------AGQ--D		-T---LNI-A-H---E--
	Longispora albida	517160007	----V--S-D-----ED--Q		LA--KRDII--F---G--
	Microbacterium testaceum	503351062	--A-Q-AK------QAD--A		LAM--V-I---Y---G--
	Nocardiopsis dassonvillei	502918323	--NQE-------E-AED--D		-D---MAI---N---D--
	Acidiphilium angustum	657195469	-Y--K--K------E-L--G		L----HQII--H-T-G--
	Actinobaculum urinale	551246223	--A-Q--Q-D-----ES--K		----SAKII-IN-AE---
	Actinomyces neuii	636805502	--K-E-KK------T-S--D		-N---LDI---H------
	Actinoplanes globisporus	521999705	---NV--K-------AD--G		LA--ARKIV--V---G--
	Porphyromonas uenonis	647608117	-YL-K---------AAI--D		E----KEIS--Y---G--
	Propionibacterium jensenii	655293072	--A-D--K-D-E-------G		----STNISVFY---E--
	Propionicicella superfundia	655299736	----D--N---A--QTD--A		LAM-NKKIV--I---G--
	Propionimicrobium lymphophilum	512613088	----Q--K------AEL-SK		-T---LAI---H-G-G--
	Pseudonocardia spinosispora	655591235	--D----K------AAQ-TG		-Q---LPISVIY---E--
	Rhodococcus pyridinivorans	493599101	--N----T------AAL-EG		-E---ANI--IV---S--
	Saccharomonospora azurea	491584514	--NQK--N----E-AES--G		VD---LAI---N---E--
	Streptomyces acidiscabies	498041574	--K-E--K------V-L-AG		-----VKIQ-YH---E--
	Thermobifida fusca	510815158	L-S-D---------AET--D		VD---LP-V--S---E--

Supplementary figure S39

Partial sequence alignment of phosphate ABC transporter substrate binding protein showing a 2 aa insert in species belonging to Clade II. A CSI in this position is also present in *Thermogladius cellulolyticus.*

			159		190
B. longum clade	Bifidobacterium breve	489926631	ELHHRTFDLALSDEI		TRQQTVDIVLLGRFLER
	Bifidobacterium sp. 12_1_47BFAA	316916379	N---Q--------D-		-----------------
	Bifidobacterium reuteri	763216497	D---K--E------V		-----T-----------
	Bifidobacterium saguini	727802416	D---K--E-----DV		-----------------
	B. longum subsp. longum	291516406	---Q--------D-		-----------------
	B. longum subsp. infantis	213524465	---Q--------D-		-----------------
	B. longum subsp. suis	672978332	---Q--------D-		-----------------
Other *Bifidobacteriales*	Bifidobacterium angulatum	489923856	---KQ--E---D-NV	EM	----II-----------
	Bifidobacterium merycicum	705457652	--QQ------NEDV	EM	----II-L---------
	Bifidobacterium callitrichos	759445745	---N--E---DENT	DL	----II-V--I------
	Bifidobacterium stellenboschense	736509908	---K--EF--DESV	EI	S---LI-V---------
	Bifidobacterium aesculapii	943597121	---K--EF--D-SV	EL	----LI-V---------
	Bifidobacterium adolescentis	500062942	A--KK--E--Q-EDW	KG	-N--LI-V--I---M--
	Bifidobacterium angulatum	489923856	---KQ--E---D-NV	EM	----II-----------
	Bifidobacterium animalis	490389254	D--LKIMQY-D-A-W	EG	-NE-LINV--IA--M--
	Bifidobacterium asteroides	504835182	K--QQ--GY----DW	KG	----LI-L--VA--M--
	Bifidobacterium bifidum	489911259	A---K--------KW	TG	S---LI-V------M--
	Bifidobacterium boum	651390508	Q---K---I--G-TW	TG	----LI-V--I------
	Bifidobacterium catenulatum	489932263	---KK--E--Q-ENW	AG	-N--LI-V--I---M--
	Bifidobacterium choerinum	639202513	D--R--LQ--Q--DF	PG	-N--LINV--I---M--
	Bifidobacterium dentium	489939269	A--KK--E--Q-EDW	QG	-N--LI-V--I---M--
	Bifidobacterium gallicum	493338232	NI-KQ--E---AEDW	EG	----V-----I---M--
	Bifidobacterium magnum	551239725	---LK-MRM-Q--DW	QV	-H--IM-V--I---M--
	Bifidobacterium minimum	551240546	A---K----V-A-DW	TG	S--DLINV---A--M--
	Bifidobacterium moukalabense	575769473	A--KK--E--Q--DW	QG	-N--LI-V--I---M--
	Bifidobacterium pseudocatenulatum	490330499	---KK--E--Q-ADW	SG	-N--LI-V--I---M--
	Bifidobacterium pseudolongum	551237836	D--KK-LEF-Q-ADF	PG	-N--LINV--I---M--
	Bifidobacterium ruminantium	651886814	A--KK--E--Q-ETW	SG	-N--LI-V--I---M--
	Bifidobacterium sp. 7101	658453299	K--QQ--GY----DW	KG	----LI-L--VA--M--
	Bifidobacterium sp. A11	639448662	K--QQ--SY----DW	KG	----LI-L--VA--M--
	Bifidobacterium sp. AGR2158	651884935	---RK-LM--Q-EDF	QG	-N--LINV--I---M--
	Bifidobacterium sp. MSTE12	570844655	A--KK--E--Q-EDW	QG	-N--LI-V--I---M--
	Bifidobacterium subtile	639065238	N---Q--SMV-D-SW	TG	-K--LI-V--I---M--
	Bifidobacterium thermacidophilum	657871287	Q---Q--E-V---QW	NG	-K--LI-V--I------
	Bifidobacterium thermophilum	505263787	Q---Q--E-V---QW	NG	-K--LI-VI-I------
	Bifidobacterium tsurumiense	651881761	D--KQM-T-VQ--DW	NS	-KE-LI-V---------
	Gardnerella vaginalis	490206795	K--EKI-E-VEGENW	KG	--R-LI-V---S--I–
	Parascardovia denticolens	493331738	A--EK--E--T--AW	PA	S---II-L-----YY--
	Scardovia inopinata	493335827	N--KQ---I-T--SW	EG	S---II-L-----YY--
	Alloscardovia omnicolens	545371579	K-FESVYTI-R--AW	NA	-VE--I-T---S-YY–
Other Bacteria	Cellulomonas massiliensis	517965771	A--ED--AAM-GGAW	DG	SP---I-VT----YY--
	Actinokineospora enzanensis	648625246	D--RHL-TILMNK-W	EH	GVASA--VT-----Y--
	Actinomadura flavalba	517486862	R--RKV--IL--PKW	KH	GVEPA---T-A--YF--
	Actinomyces gerencseriae	651274210	D--RQS-QMI-DPSN	EL	S---V--A--M------
	Actinomycetospora chiangmaiens	517141782	D--RHL-TVMMTK-W	PH	GVAPA--VT-----Y--
	Actinopolyspora halophila	516602012	D--RHL-SVVMGS-W	SH	GVSAA---T-----Y--
	Arthrobacter sp. 31Y	640200792	D--LSV-KAIA-P-W	SE	SPST---VA-AS-YF--
	Bacillus sp. EGD-AK10	545120237	K--QD--SAL-DGTW	TG	-P-E---VT-V--YY--
	Cellulosimicrobium cellulans	640260767	S--QD--TAL-DGTW	TG	-P-E---VT-V--YY--
	Corynebacterium vitaeruminis	643729555	D--SHLMAVLT-KDW	RY	STREA--MA-MA--Y--
	Dietzia alimentaria	498227221	D--SSLMRQISAPDW	PH	GSVAA--LA--A-YY--
	Gryllotalpicola ginsengisoli	551262312	A--RSV--NV-GAGW	SG	EAE----VT-AS--F--
	Jonesia quinghaiensis	656029013	AF-NDV-TTL-APNW	QG	ST---I-AT--S-YF--
	Kineosphaera limosa	493640661	R--RDM-V-LVDEGW	EG	GTESAI-VT--A-YF--
	Microbacterium yannicii	518009997	---LSV-EKV---SW	QG	EASA---AT-AS-YH--
	Nocardiopsis valliformis	516150730	R-RRKLLQRI-APGW	EY	GVEA-M-VT-V---Y--
	Promicromonospora sukumoe	518862069	---QE--NTL-APGW	QG	-V-EA--VT-V--YY--
	Pseudonocardia dioxanivorans	503438013	R--AEV-TVLMDPGW	QH	GVEAA--LT--A-----
	Rhodococcus wratislaviensis	589260185	D--R-LLT-IEDPSW	TG	SIPAA--VT-----Y--
	Ruania albidiflava	551298414	A--Q---TTT-AEDW	AG	SVP----VT--A--Y--
	Saccharomonospora saliphila	518655599	-I-RHL-SVIMDK-W	EH	GVSTA---T-----Y--
	Sanguibacter keddieii	502631550	Q--QD--QALF-ESW	NG	-T-E---VT-V--YY–
	Streptomyces roseochromogenes	559034423	---RTI-QHLMDEQW	HH	GVETA--AT-V--YY--
	Xylanimonas cellulosilytica	502643316	N--AQ--ATI-GP-W	DG	-T-EA---T-V--Y---

Supplementary Figure S40

Partial sequence alignment of PhoU family transcriptional regulator showing a 2 aa deletion that is specific for *B. longum* clade of the *Bifidobacteriaceae* family.

Group	Species	Accession	262		302
B. longum clade	B. longum subsp. longum	494112910	RVKRHSANALKVAEWLESQPS	DV	IERVWYPGLESHPGHEIA
	Bifidobacterium sp. 12_1_47BFAA	316915951	---------------------	--	------------------
	Bifidobacterium breve	644560294	--------------------A	--	---------------D—
	Bifidobacterium reuteri	763217538	---------------------	--	------------------
	Bifidobacterium saguini	727802036	--------------------A	--	------------------
	Bifidobacterium stellenboschense	736510789	-------------Q-------	--	------------------
	Bifidobacterium aesculapii	943597066	--R----------Q-------	--	------------------
	Bifidobacterium callitrichos	759444841	--E--------I-Q-------	--	V-----------------
	Bifidobacterium biavatii	705393101	---Q--R------Q----L-A	-L	V--------A--------
	B. longum subsp. infantis	239514332	---------------------	--	------------------
	B. longum subsp. suis	672976540	---------------------	--	------------------
	Bifidobacterium stellenboschense	736510789	-------------Q-------	--	------------------
	Bifidobacterium angulatum	229785028	---Q--R----I-Q---TR-E		V--------------D--
	Bifidobacterium merycicum	705459234	--RQ--R--M---Q---TR-E		V-----------------
Other Bifidobacteriales	Bifidobacterium adolescentis	547071340	---Q--R--M---Q---TR-E		V--------------D--
	Bifidobacterium angulatum	489922364	---Q--R----I-Q---TR-E		V--------------D--
	Bifidobacterium animalis	490329349	--RK------EI-Q---RR-E		------------------
	Bifidobacterium bifidum	547761739	--Q---E----I-Q---TR-E		V-----------------
	Bifidobacterium boum	651390414	---Q--R----I---F-D--Q		------------------
	Bifidobacterium catenulatum	489933039	---Q--R--M---Q---TR-E		V-----------------
	Bifidobacterium choerinum	639202307	--RK--E--MTI-H---GHAG		V---------------V-
	Bifidobacterium dentium	489933640	---Q--R------Q---TR-E		V-----------------
	Bifidobacterium moukalabense	575769598	--RQ--R------Q---TR-E		V-----------------
	Bifidobacterium pseudocatenulatum	656094014	---Q--R--M---Q---TR-E		V-----------------
	Bifidobacterium pseudolongum	551238138	--L---E--MTI-Q---GHSG		V-----------------
	Bifidobacterium ruminantium	651886666	---Q--R----I-Q---TR-E		V-----------------
	Bifidobacterium sp. AGR2158	651884851	--RK--E--MTI-H---GHDG		V-----------------
	Bifidobacterium sp. MSTE12	570844691	---Q--R------Q---TR-E		V-----------------
	Bifidobacterium subtile	639062917	---Q--K---AI-Q---QHSE		V-H------------D--
	Bifidobacterium thermacidophilum	657871396	---Q--R----I---F-GK-Q		------------------
	Bifidobacterium thermophilum	505263659	---Q--R--------F-GK-Q		------------------
	Bifidobacterium tsurumiense	651882111	---K--E---RL-Q---GHDE		--H------Q--------
Other Bacteria	Agromyces subbeticus	551271405	-ME---S---AI-RR--EHAG		----F-----------L-
	Cardiobacterium hominis	490243038	--RA--E---H--R---A--A		VA--Y--------Q-AL-
	Chromobacterium violaceum	499448808	-MEK------EL-R---A--N		V---Y--------Q--L-
	Citrobacter koseri	501081991	-IE---G---TL-Q---KH-A		V-H------A---Q-AL-
	Cronobacter helveticus	639216181	-MEK-------I-R---Q--Q		V-KLFF-------Q-QL-
	Desulfovibrio bastinii	652925044	--EKQNE--MEI-KF--NH-G		V---F-----N----DV-
	Elizabethkingia anophelis	496376418	-MQ---E-GI---QF--KH-A		--H-F--------QNDL-
	Enterobacter aerogenes	505807605	--E---S---LL-----Q--Q		V-K--F-W-A---H-DL-
	Fervidicella metallireducens	599130598	-MD--C---M-I-KY--AH-M		V-K-Y--------Q--L-
	Fusobacterium nucleatum	492579222	-ME--C---K--V-F-NKH-K		--K-Y-----T---Y---
	Halalkalicoccus jeotgali	495692883	-MD--CE--R-I-S--DEHDA		V---Y-------------
	Halobiforma nitratireducens	493722829	-MD--CK--SEI-S---DH-D		V---Y----D------L-
	Klebsiella pneumoniae	490252894	-ME-------QL-----Q--E		V----F-W-A---H-QL-
	Magnetospirillum gryphiswaldense	144898185	-IE--C-------TF---R-E		-A--L----T---Q-DL-
	Methylomicrobium buryatense	516450148	-MEK-C----DL-R---K--G		V---H--------Q--L-
	Natrialba magadii	490327500	-MD--CE--SAI----DDH-D		VD--Y-------------
	Polysphondylium pallidum	281206784	-MQ--ND----I-QF--NH-K		V---F--------Q--L-
	Pontibacter actiniarum	646889914	-MRQ-CS---EI--Y--RH-K		V-K-------E--Q--L-
	Pseudogulbenkiania ferrooxidans	545113144	-MEK------EL-R---A--N		V---Y--------Q--L-
	Pseudomonas fluorescens	489284031	-ME--CS---DL-T---Q--Q		VS--H--------Q-DL-
	Renibacterium salmoninarum	501201317	-MD---S---QI----QGR-G		V---L----QD-----L-
	Serratia marcescens	491077129	-MQ---DS--RI-Q------Q		V-N-Y----P---Q--L-
	Trichomonas vaginalis	2330885	-M-AE-E--M----Y-K-H-A		V-K-Y---F-D-E--D--

Supplementary Figure S41

Partial sequence alignment of cystathionine gamma-synthase showing a 2 aa insertion that is mainly specific for the *B. longum* clade of the order *Bifidobacteriales.* This insert is also present in *B. biavatii*, which branches with the *B. bifidum.*

Group	Species	Accession	234		274
B. longum, B. adolescentis and B. bifidum cluster	Bifidobacterium adolescentis	489905793	YKEDVEGLADTIAKARAAA	P	NQPKLIKVDTLIAWPTPGKTN
	Bifidobacterium kashiwanohense	746133033	----------------E--	-	---------------------
	Bifidobacterium merycicum	705458795	---------------QK--	-	---------------------
	Bifidobacterium stellenboschense	736510073	----------V----E---	-	D--------------------
	Bifidobacterium biavatii	705392774	----------V----E---	-	D--------------------
	Bifidobacterium callitrichos	759441679	----I-----V----E---	-	D-------HS-----------
	Bifidobacterium reuteri	763215747	----I-----V----E---	-	D-------HS-----------
	Bifidobacterium saguini	727803652	---------EV----E---	-	D-------HS-----------
	Bifidobacterium bifidum	489909839	----I----EV----EK--	-	D-------HS-----------
	Bifidobacterium breve	489926010	----------V----E---	-	D--------------------
	Bifidobacterium catenulatum	489931475	----------------E--	-	---------------------
	Bifidobacterium dentium	502666208	-----D---E-----Q---	-	---------------------
	Bifidobacterium longum	494112011	----I-----V----EK--	-	D-------HS-----------
	Bifidobacterium moukalabense	575770090	-----D---E-----Q---	-	---------------------
	Bifidobacterium ruminantium	651886384	-------------------	-	---------------------
	Bifidobacterium sp. 12_1_47BFAA	496059043	----I-----V----EK--	-	D-------HS-----------
	B. longum subsp. longum	540723742	----I-----V----E---	-	D-------HS-----------
	B. longum subsp. suis	672970798	----I-----V----E---	-	D-------HS-----------
	B. longum subsp. infantis	665780624	----I-----V----EK--	-	D-------HS-----------
	Bifidobacterium scardovii	757810757	----------V----EK--	-	D-------HS-----------
	Bifidobacterium stercoris	673003380	-------------------	-	---------------------
	Bifidobacterium aesculapii	943597266	---------EV----E---	-	D-------HS-----------
	Bifidobacterium pseudocatenulatum	490332689	----------------E--	-	---------------------
	Bifidobacterium sp. MSTE12	570842597	-----D---E-----Q---	-	---------------------
	Bifidobacterium angulatum	489922943	---------------QK--	-	-------------C-------
	Bifidobacterium tsurumiense	651882154	-----D-F-AVVE--QKE-	-	---------------------
Other *Bifidobacteriales*	Bifidobacterium psychraerophilum	705400262	-N--A--F-KVLE--EQVT		DR-------S-----------
	Bifidobacterium pullorum	705442372	----I----AVLE--EQVT		DR--F----------------
	Bifidobacterium crudilactis	736120754	----A--F-KVLE--EQVT		DR-------S-----------
	Bifidobacterium cuniculi	705445558	----I----KV-E--EQVT		DK-HF----------------
	Bifidobacterium gallinarum	705423812	----I---TKVLE--EQVT		DR--F----------------
	Bifidobacterium animalis	490328476	----I---SKV-E--EQVT		DR-HF----------------
	Bifidobacterium asteroides	504834728	---------AVLE--EQVT		DR--F------M---------
	Bifidobacterium boum	651391059	------A--AA-D--E-VT		DR-HF----------------
	Bifidobacterium choerinum	639202915	----I---TKVLE--EQVT		DR-HF----------------
	Bifidobacterium gallicum	493337659	--------TAVLE--QQVT		DK-HF----------------
	Bifidobacterium magnum	551239015	--------TAVLE--QQVT		DK-HF----------------
	Bifidobacterium minimum	551240281	-----QA--AALD--EKVT		DR--F----------------
	Bifidobacterium pseudolongum	551237083	----I---TKVLE--EQVT		DR-HF----------------
	Bifidobacterium thermacidophilum	657872133	------A--AA-D--ETVT		DR-HF----------------
	Bifidobacterium thermophilum	505263492	------A--AA-D--ETVT		DR-HF----------------
	Gardnerella vaginalis	532642991	-----QA--AA-E--QEVT		DK-HF---N-----------D
	Parascardovia denticolens	493332066	-------F-KVLE--EQVT		DR--F----------------
	Scardovia wiggsiae	494249125	----TA-F-EVLER-EKVT		DR--F------M---------
	Metascardovia criceti	516878306	----T-A--AALH--EEVT		DR-HF----------------
	Alloscardovia omnicolens	545375595	----T-A--AALH--EQVT		DR-HF----------------
Other Bacteria	Acaricomes phytoseiuli	516862039	-V------YRALVE-KNET		GR-SI-ALR-I-G--S-T-Q-
	Actinomyces urogenitalis	566244511	-L--L-A-HAALEE---ET		TR-T--RLH-I----S-T-QG
	Arthrobacter crystallopoietes	491408890	------E-FNALKA-Q-ET		SR-SI-SLR-I-G--A-K-Q-
	Cellulomonas fimi	503536953	-R---DA-HAA-EA-K-VT		DK-SF-GLR--------N---
	Jonesia quinghaiensis	656030480	-A---N--YNA-RE-NKVT		-K-SI-ALR-I-G--S-T-Q-
	Leifsonia aquatica	545657351	-T---QA-N-A-VA-QGVT		DK-S--ILK-I-G--S-K-Q-
	Leucobacter salsicius	516404821	-V--IAE-NSA-EA-K-ET		GK-S--ILK-V-G--S---Q-
	Micrococcus luteus	488944564	-T----A-Y-ALVA-K-ET		GR-S---LR-V-G--S-T-Q-
	Mobiluncus curtisii	490108765	-V-NL-A-FAAFEN-K-ET		RK-SI--LR-I-G--S-N-Q-
	Nocardioides alkalitolerans	655024026	-E---PA-Y-AVQA--GVT		D--S--VL--I----A-NAQG
	Propionibacterium acidifaciens	546156557	-E---QA-H-A-EA-KRVT		DR-SI--L--I-G--L-T-QG
	Rothia dentocariosa	490102755	-V---QE-YNA-ER-KTVT		DK-SF-E-R-I-GY-A-N-Q-
	Thermobifida fusca	510815355	-H---AA-Y-A-RA-Q-ET		ER-SF-RLR-I-G--S-N-Q-
	Trueperella pyogenes	644254012	-R----A-F-A-EA-KKVT		DK-SI-RLS-I----S-T-KG

Supplementary Figure S42

A 1 aa conserved signature insert in the transketolase protein that is specific for the *B. longum, B. bifidum,* and *B. adolescentis* cluster of the *Bifidobacteriaceae* family. The only exception observed is the presence of this insertion in *B. tsurumiense.*

Group	Organism	ID	264		301
B. asteroides *Cluster* II	Bifidobacterium sp. 7101	658453363	DVVYDPRPTPLMKAFCSR	P	GARAISGERMLLYQAVRQV
	Bifidobacterium indicum	705387988	--A-D--PSR-TQAWKN-	T	-G-----D-----------
	Bifidobacterium coryneforme	705401513	--A-E--PSR-TQAWKN-	T	-G-----D-----------
	Bifidobacterium sp. A11	639450010	----------------R-	-	-------------------
	Bifidobacterium asteroides	504834738	------------R--RRQ	-	---V---------------
Other *Bifidobacteriales*	Gardnerella vaginalis	490207969	--------SM-LSVWQQY		GC--G--E---Q--LA--
	Bifidobacterium adolescentis	547074621	---------K--Q-WR-C		-GT--G--E------MI--
	Bifidobacterium angulatum	489922918	---------D----WKGH		-GAI-G--E------MI--
	Bifidobacterium animalis	490328462	---------E--V-WRD-		-GLT-G------H---A--
	Bifidobacterium bifidum	489909866	--------SA-IR-WRAK		-GL--G--E---W--IL--
	Bifidobacterium boum	651391050	--A---H-SE-NH-WSQA		-GIS-G--E------IG--
	Bifidobacterium breve	489925998	----N----K--Q-WRAH		-G---G--E------LI--
	Bifidobacterium catenulatum	489931495	---------K--Q-WRQ-		EGT--G--E------MV--
	Bifidobacterium choerinum	639202927	---------E---VWERL		-GV-LG--E-------A--
	Bifidobacterium dentium	489936869	---------K--Q-WRRQ		-GY--G--E------MI--
	Bifidobacterium gallicum	493337178	----A--V-K--DVVGNA		-GTV---TE------IA--
	Bifidobacterium longum	513040083	--I------K--E-WRAH		-GH--G--E------LV--
	Bifidobacterium moukalabense	575770081	---------K--Q-WRRQ		-GYS-G--E-------I--
	Bifidobacterium pseudocatenulatum	656094997	---------K--Q-WRQQ		-GI--G--Q------MV--
	Bifidobacterium pseudolongum	651888265	---------A--R-WRE-		-GV--G--E-------A--
	Bifidobacterium ruminantium	651886376	---------K--L-WR-H		-GT--G--E------MV--
	Bifidobacterium sp. AGR2158	651885679	---------E--RMWGRL		-GV-LG--E-------A--
	Bifidobacterium sp. MSTE12	570841385	---------K--Q-WRRQ		-GY--G--E------MI--
	Bifidobacterium subtile	639063905	--A-----SS-QL-WSQA		-GI--G--E------IV--
	Bifidobacterium thermacidophilum	657872126	--A-----SA-NR-WSQA		-GLS-G--D------IG--
	Bifidobacterium thermophilum	505263501	--A-----SA-NR-WSQA		-GLS-G--D------IG--
	Alloscardovia omnicolens	545375560	--I------Q--QSLSA-		E-IVRG--H---R--LA--
	Metascardovia criceti	516878322	--I------DF-HSMELK		--HVRG--H---R--IA--
Other Bacteria	Cellulomonas fimi	503537088	-AA------A-SV-WSAA		-GI-VP------H---E--
	Cellulosimicrobium cellulans	640254973	---------A-SA-WARA		-GTVVG------H--AE--
	Actinomadura madurae	640498596	----A-W--A-AA-VERA		-GTVVG-FA---H------
	Actinomyces naeslundii	489878713	------W--A-AE-WARG		-GAVAP-WL---H---P--
	Actinotalea ferrariae	601041959	--A----Q-A-GT-WRAA		DG--VG------H--TE--
	Arthrobacter sanguinis	651431480	--A---W-SE-AR--AQA		-GSVV--KE------IE--
	Geodermatophilus obscurus	502714114	--L---W----AT-VTAA		-GSVVG-LE--FW--TA--
	Gordonia amicalis	639156001	--I---W----AR-VEAA		-GAVVG-LV---N--F---
	Jonesia quinghaiensis	656030546	--A---Q--A-IA-WAH-		AGVTVT------H--GE--
	Leptospira kirschneri	523624290	-I--N-LE---V--AQKA		-TKI-P-SE------MK-F
	Microbispora rosea	663728733	----A-W----AT-AGR-		-RTV-G-LP---H------
	Mycobacterium tuberculosis	489508326	-AI---W----AA-VG-A		-G-V---LQ---H--FA--
	Nesterenkonia sp. AN1	588270495	--A---W-SA-ASSWEAS		-G-VV--LV---H---K--
	Oerskovia turbata	663110286	---------A-SR-WAAA		--TVVG------H--AE--
	Paraoerskovia marina	656321465	---------E-SR-WSRA		-GVVVG------H--AE--
	Promicromonospora sukumoe	518859547	---------A-QS-WTAL		--H-VG------H--AE--
	Propionibacterium jensenii	655293474	------W----AQ-GRA-		-CEV-D-LDL-AG---G--
	Renibacterium salmoninarum	501202361	--A---W-SS-AQ--TQ-		-GVVV--LE--M----Q--
	Rhodococcus sp. R1101	515501012	-AI---W----A--AEA-		-G-VV--LH---N--FG--
	Rothia dentocariosa	490102872	--A---W-SQ-AQIWER-		-G-VV--LE------LD--
	Saccharomonospora halophila	518640455	--I---W----AR-VAEA		-G-IAT-LD---H-SFG--
	Spirillospora albida	663132879	----A-W--A-AT-VREA		-GT-VG-FP---H------
	Streptomyces scopuliridis	663356921	--L-E-W----AA-WAE-		DGAV---LDL-VH---L--
	Thermobispora bispora	502897312	----A-W----AR-VQEA		-RTVVG-FA---H------
	Xylanimonas cellulosilytica	502642037	---------A-AR-WEDL		-GVVVG------H--A---

Supplementary figure S43

Partial sequence alignment of the protein shikimate dehydrogenase showing a 1 aa insert that is specific for *B.asteroides* cluster consisting of *B. asteroides. B. sp. A11, B. sp. 7101, B. indicum, and B. coryneforme* within the *Bifidobacteriaceae*.

Group	Species	Accession	336		369
B. asteroides *Cluster* II	Bifidobacterium asteroides	504834759	LLHVPYTVADEDGLDAD	T	VRPHFAFALEKLGELH
	Bifidobacterium indicum	705388029	-----F--RN-E----K	-	--------V---D---
	Bifidobacterium coryneforme	799123342	-----F--RN-E----K	-	--------V---D---
	Bifidobacterium sp. 7101	658453333	-----------------	-	----------------
	Bifidobacterium sp. A11	639449924	-----------------	-	----------------
Other *Bifidobacteriales*	Bifidobacterium actinocoloniiforme	705421273	-----FSLDG-----EP		--R-----V------K
	Bifidobacterium adolescentis	489905949	-----FSTEG----AD-		--K-----VQ--D---
	Bifidobacterium angulatum	489922865	-----FSTEG-T--AD-		--K-----V---D---
	Bifidobacterium bifidum	489909675	-----FSTEG-TA--PA		-LK-----V---T--K
	Bifidobacterium boum	651391033	-----FST-G-N--EPQ		-LK-----V-------
	Bifidobacterium breve	489925969	-----FSTEG-T-IP-E		DLK-----VQ--D--K
	Bifidobacterium catenulatum	489931634	-----FSTEG----AD-		--K-----V-------
	Bifidobacterium dentium	489936940	-----FSTEG----ADE		--R-----V---D---
	Bifidobacterium gallicum	493337143	-----FST-S-TQ-GD-		-LR-----V------R
	Bifidobacterium longum	658454668	-----FSTEG-T-ISSE		DLK-----V---T--K
	Bifidobacterium longum subsp. longum	665771283	-----FSTEG-T-IP-E		DLK-----VQ--D--K
	Bifidobacterium longum subsp. infantis	665782312	-----FSTEG-T-IP-E		DLK-----VQ-----K
	Bifidobacterium minimum	551240311	-------TQG--A-GE-		-LR-----Q---V--R
	Bifidobacterium moukalabense	575770040	-----FSTEG----A--		--K-----V---D---
	Bifidobacterium pseudocatenulatum	490332454	-----FSTEG----AD-		--K-----V-------
	Bifidobacterium pseudolongum	551237128	-----FSTQG-EA-GE-		-LK-----V---D-VR
	Bifidobacterium ruminantium	651886245	-----FSTEG-T--AD-		--K-----V-------
	Bifidobacterium sp. 12_1_47BFAA	496058969	-----FSTEG-T-IP-E		DLK-----VQ--D--K
	Bifidobacterium sp. AGR2158	651885714	-----FSTKG-EA-GG-		-LR-----V---N-VR
	Bifidobacterium sp. MSTE12	570844907	-----FSTEG----ADE		-HR-----V---D---
	Bifidobacterium subtile	639064424	-----FST-G-S--SDE		--R-----V------E
	Bifidobacterium thermacidophilum	657872108	-----FSTEG-T--EPQ		-LK-----V-------
	Bifidobacterium thermophilum	505263520	-----FSTEG-T--EPQ		-LK-----V-------
	Bifidobacterium tsurumiense	651882186	-----FST-G-EA-GQN		-LK-----V-----VK
	Gardnerella vaginalis	490208017	-----FSTVG--A-G--		-LQ-----V---Q-IR
	Alloscardovia omnicolens	545376807	-----FST-G-EA-GD-		-LK-----S-----VR
Other Bacteria	Burkholderia fungorum	654737699	-----VDL-S-QK---E		--SWL----Q-----K
	Butyrivibrio hungatei	652955204	-------LES-HKISDE		YKK-----K---S--K
	Clostridium butyricum	488645134	-------LEN-TK-SN-		IIK--S--K---N--N
	Conexibacter woesei	502697840	-------A-R---I-PE		--GWL---A---D--R
	Corynebacterium glucuronolyticans	491532760	---------A-TD--PA		YVA-L---V------V
	Desulfotomaculum reducens	500207873	------SLKH-NH--K-		IKSWMS--R---D--S
	Eubacterium eligens	547479501	-Q-----LKH-NK-SDE		YLNY----E---V--K
	Firmicutes bacterium	547972125	-------LKH--K-SQA		YLSY----E---S---
	Jonesia quinghaiensis	656028651	-Q---H-----PE----		L-SWL---D--I--IV
	Lactobacillus helveticus	644143613	-------AE--TK-PG-		-KK-L----Q--D-IK
	Leptotrichia wadei	545622582	-------LRN-KK-SEN		ILR--S--E---S--K
	Rhodococcus hoagii	503180334	-------LSG-----RA		L-SWL--GS--I--VA
	Ruania albidiflava	551297477	-----H-T-V----SDQ		L-SWL---H--VT-VR
	Ruminococcus albus	488936640	-----C-L-N-SK-P-N		-KK---Y-E---S--A
	Syntrophobotulus glycolicus	503391289	------SLEN--NM-QK		YKN-----R---Q--G
	Syntrophomonas wolfei	499959619	-------IKN-GF-TDE		FKK-----E--M---A
	Thermobacillus composti	505068211	------S-KH-TR-PQ-		YTR-----E---R--A
	Treponema succinifaciens	503465867	-------IRN-IK-SS-		ILK--S--R---F--T
	Variovorax paradoxus	502272156	-----FSL-A--K---E		L-SWL---V------R
	Actinobaculum urinale	551245734	-------T---TD-A-E		-TQ-----V------R
	Aerococcus urinae	503434257	------SLDN-SA-PEK		YQK-----E------N
	Anaerococcus hydrogenalis	490953474	-I----R-DF--K--EK		YKK-----Y---E--N
	Anaerovorax odorimutans	653150499	-----F--EN-KK--NS		YVKY-S--A---N--E
	Arthrobacter sanguinis	651430925	-------LE--QA--PE		L-SWL---D--VA-VV
	Bacillus ginsengihumi	647285984	-----V-IKN-EK--PT		LKAGL---D---Q--Q
	Bacteroides pectinophilus	547961135	-Q-----L-N-SI-GHE		YTS-----V---D--R

Supplementary figure S44

Partial sequence alignment for 5-methyltetrahydropteroyltriglutamate--homocysteine methyltransferase showing a 1 aa insert that is specific for a subclade within the *B.asteroides* cluster consisting of *B. asteroides. B. sp. A11, B. sp. 7101, B. indicum, and B. coryneforme*.

Group	Species	Accession	253		286
B. asteroides Cluster II	Bifidobacterium asteroides	504835116	KDTDIVLQGAALKKGSG		MAEATQAALQKLIDDGV
	Bifidobacterium indicum	655535572	--------------D--		--------M-------T
	Bifidobacterium coryneforme	671341294	--------------D--		--------M-------T
	Bifidobacterium sp. A11	639448605	-----------------		----------------T
	Bifidobacterium sp. 7101	658453234	------------R----		-----------------
Other Bifidobacteriales	Bifidobacterium actinocoloniiforme	705422085	--S-V-V--I-----DT	Q	-V----K-M-------T
	Bifidobacterium adolescentis CAG:119	547079006	DVEGVAK--I-I---DQ	Q	LD--V-K-V---M---T
	Bifidobacterium angulatum	489922655	-A-GMAKEAV-V---DT	A	T-Q-V-K---------T
	Bifidobacterium animalis	490329321	A-EGVTEE-IIV---DK	Q	-D--V-K-V---M---T
	Bifidobacterium boum	651390436	--EGS-K--I-IA-NDD	Q	TTK-V-K-V---M---T
	Bifidobacterium tsurumiense	651882095	E-EGVAPM-M-IA--DE	A	TVQ-MK---D---A--T
	Bifidobacterium breve	489925140	E-VGVTPEAV-V---DS	K	T---V-K-I---M---T
	Bifidobacterium catenulatum	489932426	DVEGVAK--I-V---NT	Q	L---V-K-V---M---T
	Bifidobacterium choerinum	639201875	SAEGV-K--I-I---DT	E	L-K-V--GM-H-M---D
	Bifidobacterium dentium	489933692	DVEGVAK--I-IE--NE	K	LD--V-K-----M---T
	Bifidobacterium gallicum	493338097	ENEGVAV--VVV---DE	A	-DK-V---I---M---T
	Bifidobacterium longum	494110509	--F-E-PNAI-I---DS	Q	TT--V-K-M---M---T
	Bifidobacterium magnum	651390143	SIQ-TT---I-I---DT	Q	----V---V-H-M---T
	Bifidobacterium minimum	551241177	DE-GVAPEAV-V---DD	A	T-Q-V-K-V-------T
	Bifidobacterium moukalabense DSM 27321	575769579	E-VGVTKEAV-I---DS	D	T-K-V-------M---T
	Bifidobacterium pseudocatenulatum	490330272	DVEGVAK--I-V---NT	Q	L---V-K-V---M---T
	Bifidobacterium pseudolongum	551238123	SAEGV-K--I-I---DT	E	L-K-V---M-H-M---T
	Bifidobacterium ruminantium	651886648	DVEGVAK--I-I---DE	Q	LD--V-K-V---M---T
	Bifidobacterium sp. 12_1_47BFAA	496058643	E-AGG-PEAV-I---DT	K	T---V-K-I---M---T
	Bifidobacterium sp. AGR2158	651884864	SAEGV-K--I-I---DT	E	L-K-V--GM-H-M---D
	Bifidobacterium sp. MSTE12	570844644	E-VGVTKEAV-I---DS	D	T-K-V-------M---T
	Bifidobacterium subtile	639062975	--V--APE-IVV---DA	A	TVQ-V-K-M---M---T
	Bifidobacterium thermacidophilum	657871370	E-EGVAPE---I---DS	A	TD--V-K-----M---T
	Bifidobacterium thermophilum	651891080	E-EGVAPE---I---DS	A	TD--V-K-----M---T
	Gardnerella vaginalis	490232308	-SR-KA-M-IIAQ--DA	Q	LV--IK-GIE---QT-A
	Metascardovia criceti	648490119	EPY-VAP--IVIN-KDE	A	T-K-V-G-V-----S--
	Parascardovia denticolens	493331821	RPQEVAPM-IVIA-DNM	K	LTK-V---------S--
	Alloscardovia omnicolens	545372878	-PY--AP--IVV---DD	K	T-H-I-G---E---S--
Other Bacteria Indel (-)	Actinomyces viscosus	490701294	EIE-AAP--VVTA-ADP	Q	FTA-I---V-Y-M-H-I
	Actinopolymorpha alba	522064587	EPYGNAPY-ITVN-DED	K	F---I-K-T-AI----T
	Amycolatopsis benzoatilytica	654457880	APY-TAPY-IVVA---E	Q	FDK-L-G-V-----S--
	Anoxybacillus tepidamans	653160923	AHK-ASQS-LMFR--ND	S	LVK-VNK---DMMK--T
	Bacillus sp. SG-1	493892244	TEENAAQS-LMFR--NE	K	LV-EVNK--EEMME--T
	Burkholderia oxyphila	654766087	EPFATSPE-I-FA-ANT	Q	LRD-VHG--E--MA--T
	Clostridium kluyveri	501049685	EKLTSEPM-IGFR--DK	E	LE--V-R-VNE-KE--T
	Corynebacterium kroppenstedtii	502206774	-VV-STPKA-IVA-NDT	Q	L-D-IH--I-------T
	Halobacillus kuroshimensis	654485838	A-EA SES-LLFR--NE	E	LV--VNE--KEMKE--T
	Kutzneria albida	578013792	G-EGVAPY-I-VP-NG-	T	LK--V---V-Q--T--T
	Leptotrichia wadei	517308947	DNYGEEEY-I-A---NT	K	LV--INK-IEE-KA--T
	Nakamurella multipartita	506229658	G-EGAAPY-I-IP-EPA	E	LTP-I---M-H---T-A
	Propionibacterium acnes	488479962	GI--ASKF-VVTA-ND-	G	LSK-I-V-T-------T
	Pseudomonas syringae group	492155934	AAFSRQES-I--R--EP	E	LLA-IDK-ID--RA--T
	Pseudonocardia spinosispora	655587074	QQYGTTPY-I-IA--K-	D	FGK-I-G-V-A--A--T

Supplementary figure S45

Partial sequence alignment for ABC transporter substrate-binding protein showing a 1 aa insert that is specific for a subclade within the *B.asteroides* cluster consisting of *B. asteroides. B. sp. A11, B. sp. 7101, B. indicum, and B. coryneforme*. A CSI in this position is also found in *Streptomyces pyridomyceticus.*

(A)

Group	Species	Accession	76		127
B. asteroides *Cluster* I	Bifidobacterium asteroides	504834913	LAGLDTVTSGHVYMAA	HLLHSKSGDRHHPPIDQAGR	VDLTRLDDRQLTLIRR
	Bifidobacterium sp. 7101	658453010	------A------LNG	ADAAGVK-K-V-RQGGLR-V	----K-N-N----L--
	Bifidobacterium sp. A11	658450483	-------------LNG	TDAAGVK-R-ARRQGGRR-V	----K-N-N----L--
Other *Bifidobacteriales*	Bifidobacterium adolescentis	547083920	-----SA-----MFDG		K----MN-D----L--
	Bifidobacterium angulatum	489923277	-----SA-G--IIFNG		D----MN-N----L--
	Bifidobacterium animalis	490328206	MS---SA---S-VFQG		R---K-N-K----L--
	Bifidobacterium bifidum	504573278	-----SA----IVFDG		A-I-TMN-N---ML--
	Bifidobacterium boum	651390726	-----SA----ILLDG		A-I-TMN-K----L--
	Bifidobacterium catenulatum	489933292	-----SA-G--IIFNG		D---HMN-N----L--
	Bifidobacterium dentium	489937407	-----SA-G--IVFDG		D-----N-N----L--
	Bifidobacterium longum	494111290	-----SA----ILFGG		A----M--K----L--
	Bifidobacterium magnum	651390022	-S--------T-LVDG		Q-I-AMN-KE---L--
	Bifidobacterium moukalabense	575769734	-----SA-A--IVFDG		D---Q-N-N----L--
	Bifidobacterium pseudolongum	651883528	-S---S-----IMFQG		----T-N-K---ML--
	Bifidobacterium subtile	639061384	-----SASG-KIIFDG		A---AMN-S----L--
	Bifidobacterium thermacidophilum	657871850	-----SA----ILLDG		A-I-TMN-K----L--
	Bifidobacterium thermophilum	505263216	-----S-----ILLDG		A-I-TMN-K----L--
	Gardnerella vaginalis	490234497	-----S-N--K-LVDG		L-I-GMS-N----M--
	Scardovia inopinata	493336728	-----S--G--IFLDG		Q-I-VMS-KE---L--
Other Bacteria	Corynebacterium terpenotabidum	521255747	M-----P-A--T-LGT		T--AT-N-KA--KV--
	Frankia alni	499923276	--------R-R-FIGD		---S--S-KE--RL--
	Gordonia aichiensis	491320344	-----VA---R--IGD		T---G-S-KKM--L--
	Jonesia quinghaiensis	656029190	-----SAS---A-LGS		TEV-A-N-D---ML--
	Leifsonia aquatica	661251547	M---------R-FLGD		TEI-S-P-TE---L--
	Nocardia farcinica	499524550	-----SA---T-RIGD		T---E-T-K-M-ML--

(B)

Group	Species	Accession	34		74
B. asteroides *Cluster* I	Bifidobacterium asteroides	504834965	VTGHGSAGRLLGEFSPNLPE	S	LASMQIQDTEPETLAGHDLV
	Bifidobacterium sp. 7101	658452975	---RRT---Q---------N	-	---L---A-D-KV-------
	Bifidobacterium sp. A11	639448454	--------------------	-	---LR----K--I-------
Other *Bifidobacteriales*	Bifidobacterium adolescentis	489906871	-A--S-V-DK--KHM-HI-Q		--DLVVE--T--V-N---VI
	Bifidobacterium angulatum	489923522	-A--S-V-ESMAKHM-HI-Q		--DLVV---T--V-N---VI
	Bifidobacterium animalis	490328626	-A--S-I-HR---YQ-HI-Q		--DLIVE--T-AV-D---VI
	Bifidobacterium bifidum	489910115	-A--S-V-QK--RHM-HI-Q		--DLTVE-ITA-V-N---VI
	Bifidobacterium boum	651391446	-A--S-V-DR--KHM-HI-Q		--DLTVE--T--V-N---VI
	Bifidobacterium catenulatum	489931298	-A--S-V-DK--KHM-HI-Q		--DLVVE--T--V-N---VI
	Bifidobacterium choerinum	639202464	-A--S-V-EK--VYQ-HI-Q		--DLT-V--TADA-N---VI
	Bifidobacterium dentium	489936572	-A--S-V-DK--KHM-HI-Q		--DLVVE--T--V-N---VI
	Bifidobacterium gallicum	493337776	-A--S-V-HR---YL-HI-Q		--DLL-E-ST--V-N---VI
	Bifidobacterium longum	494110388	-A--S-V-ESMAKHM-HI-Q		--NLVVE--T--V-N---VI
	Bifidobacterium magnum	551238932	-A--S---SRF-DLM-HI-T		--D-VV---T----N---V-
	Bifidobacterium minimum	551240152	----S-V-ER--S-L-HI-Q		Y-DLRV---TADV-RD--V-
	Bifidobacterium moukalabense	575770559	-A--S-V-DR--KHM-HI-Q		--DLVVE--T--V-N---VI
	Bifidobacterium pseudolongum	651883579	-A--S-V-QP--A-Q-HI-Q		--HLIVE--T--A-N---VI
	Bifidobacterium ruminantium	651886317	-A--S-V-DK--KHM-HI-Q		--DLVVE--T--V-N---VI
	Bifidobacterium subtile	639064500	----A---ERF-RLM-HI-Q		--DLV-EE-T--V-NHY-V-
	Bifidobacterium thermophilum	505264178	-A--S-V-DR--KHM-HI-Q		--DLTV---T--V-N---VI
	Bifidobacterium tsurumiense	651882205	-A--S-V-DR--KHM-HI-Q		--DAVV---T--V-N---VI
	Alloscardovia omnicolens	545373635	-A--S---KR----T-HI-Q		-S-LT-E--T--V-S---V-
	Metascardovia criceti	516877329	-A-RS-V-HKF--YA-HI-Q		-SELVV---T--H-N---V-
Other Bacteria	Brevibacterium casei	496839181	-C--S-V-EK--RHQ-H--R		Y-DFV-A-STA-V-----V-
	Gordonia aichiensis	491320775	L-AG-N--TT--SHH---LP		--ERELLE-T--------V-
	Kocuria atrinae	515563268	----ST--QR--SLQ-H-HA		--G-EVVE-T--N-S---I-
	Leifsonia rubra	546167837	--AFSN--QR-IDVQ-H-RS		--HLEL-P---SV-----V-
	Streptomyces albulus	636565854	----SN--QP--ALQ-H-GP		--DRELAP-T--A-----V-

Supplementary figure S46

Conserved signature indels in the proteins (A) peptide ABC transporter ATP-binding protein and (B) N-acetyl-gamma-glutamyl-phosphate reductase, which are specific for a smaller subclade with the *B. asteroides* cluster consisting of *B. asteroides*, *B. sp.* A11, and *B. sp.* 7101.
